# Global, national, and regional burden of acute myeloid leukemia among 60–89 years-old individuals: insights from a study covering the period 1990 to 2019

**DOI:** 10.3389/fpubh.2023.1329529

**Published:** 2024-01-11

**Authors:** Pengyin Chen, Xinling Liu, Yao Zhao, Yuyuan Hu, Jiaxin Guo, Haiying Wang

**Affiliations:** ^1^School of Clinical Medicine, Weifang Medical University, Weifang, China; ^2^Department of Hematology, Affiliated Hospital of Weifang Medical University, Weifang, China; ^3^Clinical Research Center, Affiliated Hospital of Weifang Medical University, Weifang, China

**Keywords:** AML, burden, incidence, mortality, DALYs

## Abstract

**Background:**

Our study examined the global, national, and regional trends in the incidence, mortality, and disability-adjusted life years (DALYs) associated with older people’s acute myeloid leukemia (AML) over a 30 years period. AML, which predominantly affects individuals aged 60–89, is known for its severity and unfavorable prognosis. By providing insights into the growing burden of AML, our research highlights the urgent need for effective interventions and support at various levels.

**Methods:**

In this study, we analyzed older people with AML aged 60–89 using the Global Burden of Disease (GBD) database for 2019. Our goal was to assess trends and characteristics by examining the incidence rate, mortality rate, DALYs, and estimated annual percentage change (EAPC). We aimed to provide a comprehensive understanding of the disease’s trajectory and development.

**Results:**

In 2019, the older age group of 60 to 89 years reported 61,559 new cases of AML, with the corresponding number of deaths being 53,620, and the estimated DALYs standing at 990,656. Over the last 30 years, the incidence rate of AML in this age bracket increased by 1.67 per 100,000 people, the mortality rate rose by 1.57 per 100,000 people, and the rate of DALYs, indicative of disease burden, climbed by 1.42 per 100,000 people. High Socio-demographic Index (SDI) regions, particularly high-income North America and Australia, had the highest incidence rates. Germany had the highest incidence rate among the 204 countries analyzed, while Monaco reported the highest mortality and DALY rates. Smoking, high body mass index, occupational exposure to benzene, and formaldehyde were identified as significant risk factors associated with mortality from older people with AML in 2019.

**Conclusion:**

Our study showed that the incidence, mortality, and DALY rates of AML in the older population were strongly correlated with the SDI, and these rates have been steadily increasing. This had become an increasingly serious global health issue, particularly in areas with a high SDI. We highlighted the urgency to focus more on this disease and called for the prompt implementation of appropriate preventive and control measures.

## Introduction

AML is a type of bone marrow cancer characterized by the uncontrolled proliferation of bone marrow stem cells, leading to symptoms such as infections, anemia, and bleeding (1). While the exact mechanisms underlying AML development are not fully understood, one of the contributing factors is thought to be the oncogenic transformation of hematopoietic stem or progenitor cells. These cells acquire specific mutations during early stages, providing them with a clonal advantage for expansion. These mutations can disrupt normal hematopoiesis and ultimately drive the development of AML (2, 3). AML is a prevalent form of leukemia and tends to have a higher incidence in older adults (4). Approximately 80% of new cases occur in individuals aged 60 and above (5), and among different types of leukemia, AML has the highest mortality rate. It is estimated that in 2020, there were 19,940 new cases of AML diagnosed in the United States, with 11,180 resulting in death (6–8). This underscores the significant impact of AML as a severe illness. Compared to younger patients, older people with AML often face more complications and challenges in treatment, significantly impacting their prognosis and reducing their life expectancy (9, 10). Notably, survival rates among younger patients have shown significant improvement over the years, while survival rates for older patients have seen only marginal progress (11, 12). Consequently, it is imperative to study the epidemiological trends and characteristics of older people with AML to better understand and address this pressing healthcare issue.

Research has demonstrated that the age-standardized incidence and mortality rates of AML were often higher in developed countries compared to developing regions from 1990 to 2017. Men and older people have a higher likelihood of developing AML (13). The majority of older people are concentrated within the age range of 60–89 years. However, the complete understanding of AML incidence, mortality, DALYs rate, and corresponding EAPC in this age group remains unclear. The Global Burden of Disease Database has recently updated the incidence, mortality, and DALYs rates for 369 diseases and injuries in 204 countries and regions from 1990 to 2019 (14). Data related to AML is also encompassed within this database. Hence, we primarily utilize this database for a comprehensive analysis of the global burden of AML within the 60–89 age group, contributing to the formulation of appropriate preventive and therapeutic measures against this disease. Therefore, we selected AML patients aged 60 to 89 years from the GBD database and analyzed the incidence, mortality, DALYs, and EAPC for AML across 204 countries, 21 geographic regions, 5 SDI regions, and 6 age groups. We also identified four risk factors contributing to AML-related deaths in older adults. These analyses are essential for policymakers and clinicians to develop appropriate prevention and management strategies. Assessing the epidemiology of AML in older populations globally enables a better understanding of the disease’s distribution and trends, providing insights for improving patient care, resource allocation, and public health measures.

## Methods

### Data sources

We gathered data pertaining to older people with AML from the Global Health Data Exchange query tool^1^, encompassing a wide range of information including global, regional, and national incidence rates, incidence counts, mortality rates, mortality counts, DALYs rates, DALYs counts, and changes in case counts from 1990 to 2019. This information was sourced from vital registration data, verbal autopsy reports, surveillance data from hospitals and clinics, scientific literature reports, and expert consultations. The Bayesian meta-regression tool DisMod-MR 2.1 was used to synthesize the data mentioned above and to generate internally consistent estimates of incidence and mortality, providing a 95% uncertainty interval (95% UI). The rates of incidence, mortality, and DALYs were not age-standardized. DALYs, a measure of disease burden, comprise two components: Years of Life Lost (YLLs) and Years Lived with Disability (YLDs). YLLs are calculated by summing the years of expected life lost in each death case. YLDs are calculated by multiplying the number of cases of each disease or injury by the health loss weight attributed to that disease or injury. The total DALYs are then obtained by adding YLLs and YLDs together. The older people with AML were divided into six age groups: 60–64 years, 65–69 years, 70–74 years, 75–79 years, 80–84 years, and 85–89 years. To better understand the risk factors associated with AML, four distinct groups were established: smoking, high body mass index, occupational exposure to benzene, and occupational exposure to formaldehyde. Moreover, the 204 countries and regions included in the study were grouped into five categories based on their SDI, ranging from low SDI to high SDI (15, 16). To ensure comprehensive analysis, the global map was divided into 21 regions according to geographical location.

### Case definition

The incidence and mortality of AML were identified using the International Classification of Diseases, Ninth Revision (ICD-9) and Tenth Revision (ICD-10). Specifically, AML incidence was identified through the ICD-9 codes C92.0-C92.02, C92.3-C92.62, C93.0-C93.02, C94.0-C94.02, C94.2-C94.22, C94.4-C94.5 or ICD-10 codes 205.0–205.02, 205.2–205.32, 206.0–206.02, 207.0–207.02, 207.2–207.82. Mortality was identified through the ICD-9 codes C92.0, C92.3-C92.6, C93.0, C94.0, C94.2, C94.4-C94.5 or ICD-10 codes 205.0, 205.2–205.3, 206.0, 207.0, 207.2–207.8.

### Risk factor

In the 2019 GBD study, six steps are employed to estimate risk factors. The specific steps are as follows: first, risk-outcome pairs are established based on convincing evidence, indicating a correlation between a specific risk factor and a particular health outcome. Only these validated risk-outcome pairs are included in the GBD study’s assessment. Second, the relationship between each risk-outcome pair’s exposure level and relative risk (RR) is evaluated. This involves determining the correlation between the degree of a specific risk factor and the risk of contracting a particular disease. Third, the exposure distribution of each risk factor across different ages, genders, locations, and years is determined. This helps understand the extent to which different populations are affected by these risk factors. Fourth, the theoretical minimum risk exposure level (TMREL) for each risk factor is ascertained. TMREL refers to the minimum exposure level with the least health impact in the absence of any exposure. Fifth, the population attributable fraction (PAF) for each risk-outcome pair is estimated using their RR, exposure levels, and TMREL. PAF indicates the proportion of health issues in the entire population caused by a specific risk factor. This information is then used to simulate PAFs, determining the number of deaths attributed to that risk factor. Finally, the combined PAF and attributable burden for multiple risk factors are estimated, considering the interactions and cumulative effects between different factors (17).

### Statistical analysis

Linear regression can effectively estimate the trends of indicators over time in public health and epidemiological research, even though the actual time-series data may be more complex. Therefore, we utilized linear regression to estimate the annual average percentage change and its corresponding 95% uncertainty interval (18, 19). The specific calculation formula is: log (*y*) = *α* + *β* × *t* + *ϵ*, EAPC = 100 × (exp (*β*) − 1). Here, *y* represents the incidence, mortality, or DALYs rates, *t* represents the year, *α* and *β* are regression coefficients, and *ϵ* is the error term. The EAPC indicates the changing trend in AML rates among the older population. When both the EAPC value and the lower boundary of the 95% confidence interval are greater than 0, it suggests an increasing trend in rates. Conversely, if both the EAPC value and the upper boundary of the 95% confidence interval are less than 0, it indicates a declining trend in rates. The SDI is a composite indicator used to assess the level of socio-economic development of a region or country. It incorporates data on various factors, including *per capita* income, educational attainment, and health status (20). Furthermore, we examined the correlation coefficients between EAPC and SDI, EAPC and incidence rates, and incidence rates and SDI. A higher correlation coefficient suggests a stronger relationship between the variables. All statistical analyses were considered significant at *p* < 0.05. For data processing and visualization, we utilized multiple R packages in R Studio version 4.3.1 including ggplot2, tidyverse, maps, flextable, rgdal, dplyr, and ggmap. Through histograms and bar charts, we presented the temporal trends and quantities of AML incidence, mortality, and DALYs from 1990 to 2019, stratified by gender. The global burden of older people with AML was illustrated using a world map, displaying the disease burden in 204 countries and regions. Additionally, scatter plots with regression curves were used to depict the associations between EAPC and SDI, EAPC and incidence rates, as well as incidence rates and SDI.

## Results

### The global burden of older people with AML

**Figure 1:**
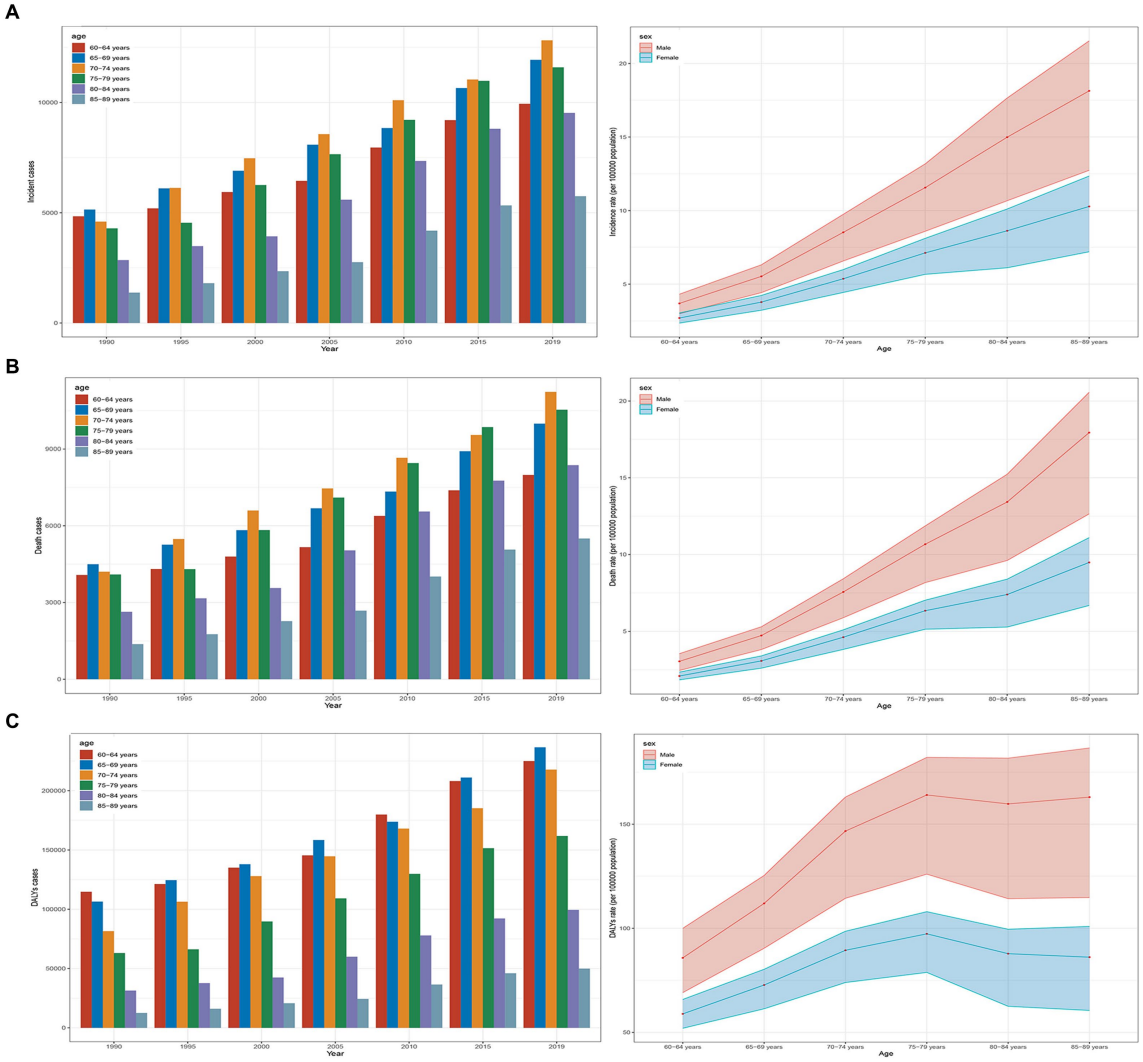
Trends in AML incidence, deaths, and DALYs among older people from 1990 to 2019. **(A)** Trends in incident cases and incidence rate. **(B)** Trends in death cases and death rate. **(C)** Trends in DALYs cases and DALYs rate.

Between 1990 and 2019, there was a significant increase in the incidence, mortality, and DALYs for older AML patients. The number of incidence increased to 61,559 (95% UI, 51,948 to 67,801), deaths to 53,620 (95% UI, 44,932 to 57,550), and DALYs to 990,656 (95% UI, 840,840 to 1,061,273), representing increases of 1.67 (95% UI, 1.18 to 2), 1.57 (95% UI, 1.11 to 1.82), and 1.42 (95% UI, 1.01 to 1.65), respectively. The rates per 100,000 population for incidence, mortality, and DALYs also showed an upward trend, increasing to 6.08 (95% UI, 5.13 to 6.69), 5.29 (95% UI, 4.43 to 5.68), and 97.78 (95% UI, 82.99 to 104.75) respectively (Table 1 and Figures 1A–C). The corresponding EAPCs were 1 (95% CI, 0.87 to 1.13), 0.86 (95% CI, 0.75 to 0.98), and 0.6 (95% CI, 0.51 to 0.69) (Table 1 and Supplementary Figure S1). In different age groups, the highest increases in incidence, mortality, and DALY rates were observed in the 85–89 age group, at 3.18 (95% UI, 2.1 to 3.85), 3.01 (95% UI, 2.03 to 3.51), and 2.99 (95% UI, 2.01 to 3.5), respectively, with the highest EAPC growth rates of 1.63 (95% CI, 1.45 to 1.81), 1.49 (95% CI, 1.32 to 1.67), and 1.47 (95% UI, 1.3 to 1.64). The 60–64 age group exhibited the lowest increases in incidence, mortality, and DALY rates, at 1.05 (95% UI, 0.8 to 1.29), 0.96 (95% UI, 0.74 to 1.16), and 0.96 (95% UI, 0.74 to 1.16), respectively, with the lowest EAPC growth rates of 0.26 (95% CI, 0.13 to 0.38), 0.1 (95% CI, 0.01 to 0.19), and 0.1 (95% CI, 0.01 to 0.19) (Table 1 and Figures 1A–C; Supplementary Figure S1).

**Table 1:** The incidence, death, and DALYs for older people with AML at the global and regional levels between 1990 and 2019.

Characteristics	1990		2019		1990–2019	
	Incident cases	Incidence rate	Incident cases	Incidence rate	Cases change	EAPC
	NO. (95% UI)	NO. (95% UI)	NO. (95% UI)	NO. (95% UI)	NO. (95% UI)	NO. (95% CI)
Global	23098 (21485 to 26410)	4.81 (4.47 to 5.5)	61559 (51948 to 67801)	6.08 (5.13 to 6.69)	1.67 (1.18 to 2)	1 (0.87 to 1.13)
SDI						
High	13600 (12513 to 15642)	10.21 (9.39 to 11.74)	33228 (25741 to 37330)	14.2 (11 to 15.96)	1.44 (0.94 to 1.75)	1.36 (1.24 to 1.48)
High-middle	5214 (4808 to 6423)	3.89 (3.59 to 4.79)	13671 (11227 to 15211)	5.28 (4.33 to 5.87)	1.62 (0.94 to 2.05)	1.24 (1.02 to 1.45)
Middle	2329 (2013 to 2962)	1.96 (1.7 to 2.5)	8513 (7541 to 10035)	2.82 (2.5 to 3.33)	2.54 (1.94 to 3.33)	1.28 (1.18 to 1.38)
Low-middle	1408 (1112 to 1829)	2.08 (1.64 to 2.7)	4658 (4120 to 5725)	2.9 (2.56 to 3.56)	2.29 (1.64 to 3.14)	1.13 (1.07 to 1.18)
Low	534 (382 to 734)	2.02 (1.44 to 2.77)	1457 (1050 to 1843)	2.55 (1.84 to 3.23)	1.6 (1.1 to 2.19)	0.84 (0.83 to 0.85)
Age						
60–64 years	4839 (4397 to 5413)	3.01 (2.74 to 3.37)	9940 (8720 to 10930)	3.18 (2.79 to 3.5)	1.05 (0.8 to 1.29)	0.26 (0.13 to 0.38)
65–69 years	5144 (4774 to 5847)	4.17 (3.87 to 4.73)	11933 (10358 to 13170)	4.61 (4.01 to 5.09)	1.32 (0.95 to 1.61)	0.56 (0.42 to 0.69)
70–74 years	4598 (4237 to 5438)	5.44 (5.01 to 6.43)	12819 (10840 to 14133)	6.85 (5.79 to 7.55)	1.79 (1.27 to 2.16)	0.86 (0.76 to 0.95)
75–79 years	4290 (3932 to 5079)	7 (6.41 to 8.28)	11592 (9438 to 12890)	9.12 (7.43 to 10.15)	1.7 (1.07 to 2.1)	1.1 (1.01 to 1.19)
80–84 years	2850 (2515 to 3465)	8.09 (7.14 to 9.84)	9525 (7276 to 10866)	11.28 (8.62 to 12.87)	2.34 (1.5 to 2.9)	1.42 (1.29 to 1.56)
85–89 years	1377 (1166 to 1685)	9.14 (7.74 to 11.18)	5750 (4314 to 6670)	13.22 (9.92 to 15.34)	3.18 (2.1 to 3.85)	1.63 (1.45 to 1.81)
Regions						
Andean Latin America	69 (54 to 95)	2.93 (2.32 to 4.04)	344 (240 to 434)	5.17 (3.61 to 6.52)	4.02 (2.06 to 6.1)	2.42 (2.22 to 2.62)
Australasia	421 (371 to 459)	13.86 (12.2 to 15.12)	986 (739 to 1245)	15.97 (11.96 to 20.16)	1.34 (0.83 to 1.97)	0.28 (0.2 to 0.37)
Caribbean	104 (91 to 119)	3.27 (2.87 to 3.76)	263 (216 to 311)	4.2 (3.46 to 4.97)	1.53 (1.06 to 2.06)	1.15 (1.03 to 1.27)
Central Asia	145 (123 to 197)	2.61 (2.2 to 3.54)	297 (247 to 350)	3.55 (2.96 to 4.19)	1.04 (0.52 to 1.49)	1.68 (1.38 to 1.98)
Central Europe	1049 (943 to 1469)	5.49 (4.93 to 7.68)	2614 (1866 to 3057)	9.17 (6.55 to 10.73)	1.49 (0.37 to 2.1)	2.24 (1.99 to 2.48)
Central Latin America	265 (243 to 332)	2.78 (2.55 to 3.48)	1273 (1059 to 1494)	4.52 (3.76 to 5.3)	3.8 (2.82 to 4.74)	1.73 (1.6 to 1.86)
Central Sub-Saharan Africa	29 (19 to 43)	1.15 (0.76 to 1.71)	72 (43 to 110)	1.3 (0.78 to 1.99)	1.48 (0.51 to 2.83)	0.43 (0.26 to 0.6)
East Asia	1036 (778 to 1428)	0.99 (0.75 to 1.37)	4377 (3531 to 5273)	1.66 (1.34 to 2)	3.23 (1.89 to 5.06)	2.19 (2.01 to 2.37)
Eastern Europe	1466 (1263 to 1748)	4.03 (3.47 to 4.8)	1676 (1468 to 2040)	3.67 (3.22 to 4.47)	0.14 (−0.05 to 0.34)	−0.61 (−0.79 to −0.43)
Eastern Sub-Saharan Africa	162 (104 to 241)	1.93 (1.24 to 2.88)	418 (252 to 573)	2.39 (1.44 to 3.28)	1.59 (0.91 to 2.42)	0.81 (0.77 to 0.84)
High-income Asia Pacific	1524 (1409 to 1747)	6.07 (5.62 to 6.96)	5026 (3889 to 5949)	9.34 (7.23 to 11.06)	2.3 (1.49 to 2.92)	1.74 (1.49 to 1.99)
High-income North America	5676 (5202 to 6592)	12.58 (11.53 to 14.61)	13221 (10787 to 15271)	16.49 (13.46 to 19.05)	1.33 (0.91 to 1.72)	0.93 (0.75 to 1.11)
North Africa and Middle East	933 (732 to 1173)	4.76 (3.73 to 5.98)	2673 (2244 to 3257)	5.51 (4.63 to 6.71)	1.86 (1.34 to 2.6)	0.53 (0.46 to 0.6)
Oceania	10 (7 to 14)	3.02 (2.08 to 4.42)	25 (18 to 35)	3.37 (2.44 to 4.73)	1.5 (0.91 to 2.33)	0.34 (0.26 to 0.43)
South Asia	1557 (1213 to 1984)	2.49 (1.94 to 3.17)	5362 (4468 to 6613)	3.21 (2.67 to 3.96)	2.44 (1.66 to 3.52)	0.76 (0.67 to 0.85)
Southeast Asia	794 (660 to 1048)	2.76 (2.29 to 3.64)	2768 (2235 to 3550)	3.9 (3.15 to 5)	2.49 (1.75 to 3.56)	1.22 (1.14 to 1.29)
Southern Latin America	272 (235 to 322)	4.67 (4.03 to 5.53)	704 (540 to 889)	6.75 (5.18 to 8.52)	1.59 (0.96 to 2.36)	1.21 (0.96 to 1.45)
Southern Sub-Saharan Africa	14 (10 to 24)	0.44 (0.32 to 0.76)	35 (27 to 47)	0.53 (0.41 to 0.71)	1.49 (0.76 to 2.52)	0.71 (0.59 to 0.83)
Tropical Latin America	472 (424 to 541)	4.43 (3.98 to 5.08)	1843 (1484 to 2013)	6.26 (5.04 to 6.83)	2.91 (2.23 to 3.27)	1.37 (1.22 to 1.52)
Western Europe	6999 (6431 to 8434)	9.39 (8.63 to 11.31)	17298 (12653 to 20155)	15.8 (11.56 to 18.41)	1.47 (0.76 to 1.94)	2.18 (2.04 to 2.32)
Western Sub-Saharan Africa	101 (80 to 136)	1.01 (0.8 to 1.36)	285 (212 to 352)	1.42 (1.06 to 1.75)	1.81 (1.18 to 2.57)	1.4 (1.32 to 1.49)

Characteristics	1990		2019		1990–2019	
	Death cases	Death rate	Death cases	Death rate	Cases change	EAPC
	NO.(95% UI)	NO.(95% UI)	NO.(95% UI)	NO.(95% UI)	NO.(95% UI)	NO.(95% CI)
Global	20879 (19441 to 23932)	4.35 (4.05 to 4.98)	53620 (44932 to 57550)	5.29 (4.43 to 5.68)	1.57 (1.11 to 1.82)	0.86 (0.75 to 0.98)
SDI						
High	11851 (10899 to 13615)	8.9 (8.18 to 10.22)	27975 (21918 to 30162)	11.96 (9.37 to 12.89)	1.36 (0.9 to 1.54)	1.21 (1.11 to 1.32)
High-middle	4960 (4568 to 6146)	3.7 (3.41 to 4.59)	12425 (10143 to 13461)	4.8 (3.92 to 5.2)	1.51 (0.86 to 1.83)	1.1 (0.91 to 1.29)
Middle	2213 (1914 to 2812)	1.87 (1.61 to 2.37)	7669 (6769 to 9133)	2.54 (2.24 to 3.03)	2.46 (1.85 to 3.24)	1.12 (1.04 to 1.19)
Low-middle	1337 (1060 to 1736)	1.98 (1.57 to 2.56)	4206 (3687 to 5323)	2.61 (2.29 to 3.31)	2.15 (1.52 to 2.98)	0.97 (0.94 to 0.99)
Low	507 (362 to 697)	1.92 (1.37 to 2.63)	1318 (951 to 1682)	2.31 (1.67 to 2.95)	1.6 (1.09 to 2.19)	0.68 (0.64 to 0.72)
Age						
60–64 years	4075 (3731 to 4593)	2.54 (2.32 to 2.86)	7986 (7139 to 8731)	2.56 (2.28 to 2.79)	0.96 (0.74 to 1.16)	0.1 (0.01 to 0.19)
65–69 years	4495 (4184 to 5113)	3.64 (3.39 to 4.14)	9992 (8748 to 10761)	3.86 (3.38 to 4.16)	1.22 (0.9 to 1.44)	0.39 (0.3 to 0.49)
70–74 years	4204 (3885 to 4946)	4.97 (4.6 to 5.85)	11234 (9652 to 12057)	6 (5.16 to 6.44)	1.67 (1.2 to 1.94)	0.67 (0.62 to 0.73)
75–79 years	4096 (3779 to 4837)	6.68 (6.16 to 7.89)	10537 (8604 to 11424)	8.29 (6.77 to 8.99)	1.57 (1.03 to 1.85)	0.88 (0.81 to 0.96)
80–84 years	2635 (2346 to 3176)	7.48 (6.66 to 9.02)	8369 (6465 to 9297)	9.91 (7.66 to 11.01)	2.18 (1.4 to 2.57)	1.26 (1.12 to 1.4)
85–89 years	1373 (1168 to 1664)	9.11 (7.75 to 11.04)	5502 (4154 to 6243)	12.65 (9.55 to 14.36)	3.01 (2.03 to 3.51)	1.49 (1.32 to 1.67)
Regions						
Andean Latin America	66 (53 to 92)	2.82 (2.25 to 3.93)	312 (219 to 392)	4.69 (3.29 to 5.89)	3.73 (1.88 to 5.68)	2.23 (2.03 to 2.43)
Australasia	407 (357 to 441)	13.41 (11.75 to 14.5)	938 (739 to 1069)	15.19 (11.97 to 17.32)	1.3 (0.94 to 1.61)	0.23 (0.14 to 0.31)
Caribbean	100 (88 to 115)	3.15 (2.78 to 3.62)	238 (196 to 280)	3.8 (3.14 to 4.47)	1.38 (0.96 to 1.87)	0.95 (0.8 to 1.1)
Central Asia	135 (114 to 182)	2.42 (2.04 to 3.27)	259 (216 to 306)	3.1 (2.58 to 3.66)	0.92 (0.43 to 1.35)	1.51 (1.19 to 1.84)
Central Europe	998 (898 to 1400)	5.22 (4.7 to 7.32)	2365 (1676 to 2767)	8.3 (5.88 to 9.71)	1.37 (0.29 to 1.94)	2.08 (1.85 to 2.31)
Central Latin America	253 (232 to 318)	2.65 (2.43 to 3.34)	1151 (968 to 1354)	4.09 (3.44 to 4.81)	3.55 (2.56 to 4.48)	1.56 (1.45 to 1.66)
Central Sub-Saharan Africa	28 (19 to 41)	1.1 (0.73 to 1.62)	65 (39 to 100)	1.18 (0.7 to 1.8)	1.34 (0.43 to 2.57)	0.26 (0.06 to 0.45)
East Asia	983 (727 to 1364)	0.94 (0.7 to 1.31)	3954 (3181 to 4739)	1.5 (1.21 to 1.8)	3.02 (1.75 to 4.79)	2.03 (1.85 to 2.21)
Eastern Europe	1370 (1174 to 1636)	3.76 (3.23 to 4.5)	1486 (1301 to 1807)	3.26 (2.85 to 3.96)	0.08 (−0.11 to 0.28)	−0.75 (−0.92 to −0.59)
Eastern Sub-Saharan Africa	154 (99 to 229)	1.84 (1.18 to 2.73)	383 (232 to 522)	2.19 (1.33 to 2.99)	1.48 (0.85 to 2.28)	0.68 (0.63 to 0.73)
High-income Asia Pacific	1465 (1355 to 1667)	5.84 (5.4 to 6.64)	4671 (3650 to 5121)	8.68 (6.78 to 9.52)	2.19 (1.45 to 2.48)	1.64 (1.42 to 1.86)
High-income North America	5027 (4609 to 5802)	11.14 (10.21 to 12.86)	11427 (9474 to 12316)	14.26 (11.82 to 15.36)	1.27 (0.91 to 1.47)	0.82 (0.64 to 1)
North Africa and Middle East	882 (693 to 1108)	4.5 (3.53 to 5.65)	2396 (2014 to 2919)	4.94 (4.15 to 6.02)	1.72 (1.21 to 2.46)	0.36 (0.31 to 0.41)
Oceania	9 (6 to 14)	2.87 (1.97 to 4.19)	22 (16 to 31)	3.04 (2.21 to 4.27)	1.37 (0.82 to 2.11)	0.17 (0.11 to 0.23)
South Asia	1476 (1140 to 1880)	2.36 (1.82 to 3)	4838 (4023 to 6063)	2.9 (2.41 to 3.63)	2.28 (1.52 to 3.29)	0.6 (0.53 to 0.67)
Southeast Asia	760 (633 to 996)	2.64 (2.2 to 3.46)	2493 (2020 to 3175)	3.51 (2.85 to 4.47)	2.28 (1.61 to 3.3)	1.03 (0.97 to 1.08)
Southern Latin America	260 (225 to 306)	4.47 (3.86 to 5.26)	639 (553 to 731)	6.13 (5.31 to 7.01)	1.46 (1.08 to 1.91)	1.02 (0.8 to 1.24)
Southern Sub-Saharan Africa	13 (9 to 23)	0.41 (0.29 to 0.72)	31 (24 to 41)	0.48 (0.37 to 0.63)	1.39 (0.68 to 2.35)	0.58 (0.47 to 0.69)
Tropical Latin America	450 (403 to 516)	4.22 (3.78 to 4.84)	1669 (1345 to 1816)	5.66 (4.56 to 6.17)	2.71 (2.07 to 3.06)	1.19 (1.07 to 1.32)
Western Europe	5944 (5486 to 7236)	7.97 (7.36 to 9.71)	14026 (10543 to 15277)	12.81 (9.63 to 13.95)	1.36 (0.73 to 1.61)	1.99 (1.86 to 2.11)
Western Sub-Saharan Africa	97 (77 to 129)	0.97 (0.76 to 1.29)	256 (195 to 316)	1.27 (0.97 to 1.57)	1.64 (1.08 to 2.31)	1.19 (1.1 to 1.29)

Characteristics	1990		2019		1990–2019	
	DALYs cases	DALYs rate	DALYs cases	DALYs rate	Cases change	EAPC
	NO.(95% UI)	NO.(95% UI)	NO.(95% UI)	NO.(95% UI)	NO.(95% UI)	NO.(95% CI)
Global	409665 (384065 to 464967)	85.3 (79.97 to 96.82)	990656 (840840 to 1061273)	97.78 (82.99 to 104.75)	1.42 (1.01 to 1.65)	0.6 (0.51 to 0.69)
SDI						
High SDI	220966 (204335 to 252649)	165.86 (153.37 to 189.64)	487157 (389291 to 520661)	208.21 (166.39 to 222.53)	1.2 (0.81 to 1.36)	0.94 (0.85 to 1.04)
High-middle SDI	102454 (94791 to 124104)	76.45 (70.73 to 92.6)	234244 (193370 to 253794)	90.43 (74.65 to 97.98)	1.29 (0.74 to 1.57)	0.7 (0.55 to 0.84)
Middle SDI	46458 (40111 to 59082)	39.17 (33.82 to 49.81)	154769 (136704 to 185026)	51.29 (45.3 to 61.32)	2.33 (1.74 to 3.08)	0.94 (0.89 to 0.99)
Low-middle SDI	28537 (22596 to 36870)	42.17 (33.39 to 54.48)	86295 (75775 to 108654)	53.64 (47.1 to 67.54)	2.02 (1.42 to 2.82)	0.8 (0.77 to 0.83)
Low SDI	11044 (7860 to 15100)	41.73 (29.7 to 57.06)	27679 (20056 to 35423)	48.52 (35.16 to 62.09)	1.51 (1 to 2.1)	0.55 (0.51 to 0.6)
Age						
60–64 years	114725 (105042 to 129133)	71.42 (65.39 to 80.38)	225008 (201119 to 246077)	71.99 (64.35 to 78.74)	0.96 (0.74 to 1.16)	0.1 (0.01 to 0.19)
65–69 years	106433 (98966 to 121014)	86.19 (80.14 to 98)	236596 (207284 to 255116)	91.5 (80.16 to 98.66)	1.22 (0.9 to 1.44)	0.39 (0.3 to 0.49)
70–74 years	81525 (75339 to 96007)	96.46 (89.14 to 113.6)	217775 (186693 to 234208)	116.4 (99.79 to 125.19)	1.67 (1.2 to 1.94)	0.67 (0.62 to 0.73)
75–79 years	63041 (58053 to 74585)	102.83 (94.69 to 121.65)	161830 (131949 to 175343)	127.37 (103.85 to 138.01)	1.57 (1.02 to 1.85)	0.87 (0.8 to 0.95)
80–84 years	31419 (27884 to 37821)	89.21 (79.17 to 107.39)	99480 (76712 to 110464)	117.84 (90.87 to 130.85)	2.17 (1.39 to 2.55)	1.24 (1.1 to 1.38)
85–89 years	12523 (10635 to 15223)	83.11 (70.57 to 101.03)	49968 (37810 to 56669)	114.92 (86.96 to 130.33)	2.99 (2.01 to 3.5)	1.47 (1.3 to 1.64)
Regions						
Andean Latin America	1309 (1031 to 1795)	55.98 (44.09 to 76.73)	5939 (4164 to 7461)	89.22 (62.55 to 112.08)	3.54 (1.77 to 5.45)	2.03 (1.84 to 2.22)
Australasia	7551 (6620 to 8164)	248.5 (217.83 to 268.65)	16048 (12762 to 18175)	259.95 (206.72 to 294.4)	1.13 (0.8 to 1.4)	−0.02 (−0.12 to 0.07)
Caribbean	1956 (1722 to 2232)	61.67 (54.28 to 70.37)	4635 (3820 to 5464)	74.17 (61.14 to 87.44)	1.37 (0.94 to 1.87)	0.93 (0.8 to 1.06)
Central Asia	3140 (2649 to 4240)	56.41 (47.59 to 76.16)	5954 (4934 to 7025)	71.27 (59.06 to 84.1)	0.9 (0.41 to 1.34)	1.32 (0.99 to 1.66)
Central Europe	20594 (18594 to 28524)	107.73 (97.27 to 149.21)	45664 (32699 to 53518)	160.23 (114.74 to 187.79)	1.22 (0.24 to 1.76)	1.77 (1.59 to 1.95)
Central Latin America	5154 (4760 to 6395)	54.05 (49.91 to 67.05)	22570 (18940 to 26753)	80.13 (67.24 to 94.98)	3.38 (2.48 to 4.28)	1.4 (1.31 to 1.49)
Central Sub-Saharan Africa	617 (411 to 927)	24.41 (16.27 to 36.66)	1425 (854 to 2204)	25.76 (15.43 to 39.84)	1.31 (0.4 to 2.6)	0.2 (−0.04 to 0.43)
East Asia	20669 (15332 to 28711)	19.8 (14.69 to 27.51)	78266 (62910 to 94292)	29.74 (23.91 to 35.83)	2.79 (1.59 to 4.43)	1.81 (1.65 to 1.97)
Eastern Europe	30578 (25822 to 36609)	84.04 (70.97 to 100.61)	31098 (27110 to 37685)	68.19 (59.45 to 82.64)	0.02 (−0.16 to 0.21)	−1.12 (−1.33 to −0.92)
Eastern Sub-Saharan Africa	3383 (2165 to 5038)	40.41 (25.87 to 60.18)	8091 (4945 to 10877)	46.33 (28.32 to 62.28)	1.39 (0.76 to 2.19)	0.55 (0.49 to 0.6)
High-income Asia Pacific	28689 (26668 to 32233)	114.31 (106.26 to 128.44)	79077 (63404 to 85790)	146.99 (117.86 to 159.47)	1.76 (1.18 to 1.99)	1.1 (0.88 to 1.32)
High-income North America	92630 (85143 to 107663)	205.3 (188.71 to 238.62)	203805 (172674 to 218281)	254.26 (215.42 to 272.32)	1.2 (0.87 to 1.38)	0.71 (0.58 to 0.84)
North Africa and Middle East	19093 (15035 to 23942)	97.31 (76.63 to 122.02)	49743 (41494 to 59893)	102.52 (85.52 to 123.44)	1.61 (1.1 to 2.3)	0.14 (0.12 to 0.17)
Oceania	198 (135 to 291)	60.46 (41.15 to 89.16)	465 (329 to 657)	63.57 (44.93 to 89.75)	1.35 (0.76 to 2.17)	0.19 (0.14 to 0.23)
South Asia	31794 (24579 to 40480)	50.79 (39.26 to 64.66)	99916 (83740 to 125711)	59.81 (50.13 to 75.25)	2.14 (1.41 to 3.12)	0.43 (0.35 to 0.51)
Southeast Asia	15572 (12835 to 20746)	54.06 (44.56 to 72.03)	50347 (40543 to 64919)	70.94 (57.12 to 91.47)	2.23 (1.56 to 3.24)	0.92 (0.87 to 0.97)
Southern Latin America	5201 (4448 to 6065)	89.3 (76.39 to 104.15)	12158 (10498 to 13947)	116.59 (100.67 to 133.74)	1.34 (0.97 to 1.79)	0.85 (0.66 to 1.04)
Southern Sub-Saharan Africa	292 (206 to 509)	9.14 (6.46 to 15.95)	704 (540 to 921)	10.69 (8.2 to 13.99)	1.41 (0.71 to 2.42)	0.65 (0.54 to 0.75)
Tropical Latin America	9225 (8269 to 10471)	86.61 (77.64 to 98.31)	32269 (26316 to 35108)	109.52 (89.32 to 119.15)	2.5 (1.93 to 2.83)	0.97 (0.87 to 1.06)
Western Europe	109962 (102507 to 133299)	147.48 (137.48 to 178.78)	237146 (181545 to 256266)	216.58 (165.8 to 234.04)	1.16 (0.62 to 1.37)	1.61 (1.49 to 1.74)
Western Sub-Saharan Africa	2057 (1609 to 2754)	20.53 (16.06 to 27.49)	5337 (4060 to 6601)	26.53 (20.18 to 32.81)	1.59 (1.05 to 2.29)	1.11 (1.01 to 1.21)

CI, confidence interval; EAPC, estimated annual percentage change; UI, uncertainty interval.

In 1990, the highest number of incidence and deaths in all age groups was in the 65–69 age group, with 5,144 (95% UI, 4,774 to 5,847) cases and 4,495 (95% UI, 4,184 to 5,113) cases, respectively (Figures 1A,B and Table 1). The highest DALYs in the 60–64 age group were 114,725 (95% UI, 105,042 to 129,133) cases (Figure 1C and Table 1). In 2019, the highest number of incidence and deaths among all age groups was in the 70–74 age group, with 12,819 (95% UI, 10,840 to 14,133) cases and 11,234 (95% UI, 9,652 to 12,057) cases, respectively, while the 65–69 age group had the highest number of DALYs, at 236,596 (95% UI, 207,284 to 255,116) cases. The 85–89 age group had the lowest numbers for incidence, deaths, and DALYs, at 5,750 (95% UI, 4,314 to 6,670), 5,502 (95% UI, 4,154 to 6,243), and 49,968 (95% UI, 37,810 to 56,669) cases, respectively, but had the highest rates per 100,000 population for incidence, mortality, and DALYs, at 13.22 (95% UI, 9.92 to 15.34), 12.65 (95% UI, 9.55 to 14.36), and 127.37 (95% UI, 103.85 to 138.01) cases, respectively. Across all age groups, men had higher rates of incidence, mortality, and DALYs than women (Table 1 and Figures 1A–C).

### Burden of older people with AML in 5 SDI regions

**Figure 2:**
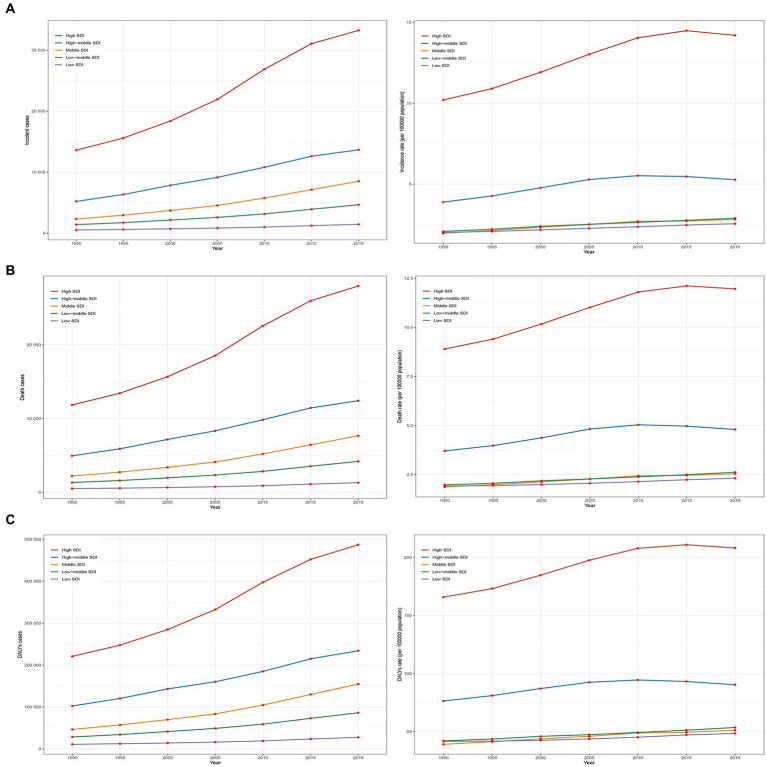
Epidemiologic trends of incidence, death, and DALYs rates in 5 SDI regions of AML in older peoples from 1990 to 2019. **(A)** Trends in incident cases and incidence rate. **(B)** Trends in death cases and mortality rate. **(C)** Trends in DALYs cases and DALYs rate. SDI, socio-demographic index.

From 1990 to 2019, the incidence, incidence rate, mortality, mortality rate, DALYs, and DALYs rate of older people with AML in high SDI regions were significantly higher than in the other four SDI regions (Figures 2A–C). The incidence rate in high SDI regions showed a substantial annual increase with an EAPC of 1.36 (95% CI, 1.24 to 1.48), while the mortality rate exhibited an upward trend with an EAPC of 1.12 (95% CI, 1.04 to 1.19), and the DALYs rate showed a moderate rise with an EAPC of 0.94 (95% CI, 0.85 to 1.04) (Table 1 and Supplementary Figure S2). Among the SDI regions, the middle SDI region demonstrated the highest increases in incidence, mortality, and DALYs compared to the other four regions, with significant growth rates. The incidence saw an impressive increase of 2.54 (95% UI, 1.94 to 3.33), the mortality experienced a substantial rise of 2.46 (95% UI, 1.85 to 3.24), and the DALYs showed a notable increase of 2.33 (95% UI, 1.74 to 3.08) (Table 1). In 2019, the low SDI region had the lowest incidence, incidence rate, mortality, mortality rate, DALYs, and DALYs rate compared to the other four regions (Figures 2A–C). The incidence stood at 1457 (95% UI, 1050 to 1843) cases, with an incidence rate of 2.55 (95% UI, 1.84 to 3.23) per 100,000 population. The mortality accounted for 1318 (95% UI, 951 to 1682) deaths, with a mortality rate of 2.31 (95% UI, 1.67 to 2.95) per 100,000 population. The DALYs totaled 27679 (95% UI, 20056 to 35423), with a DALYs rate of 48.52 (95% UI, 35.16 to 62.09) per 100,000 population (Table 1).

### Burden of older people with AML in 21 geographic regions

**Figure 3:**
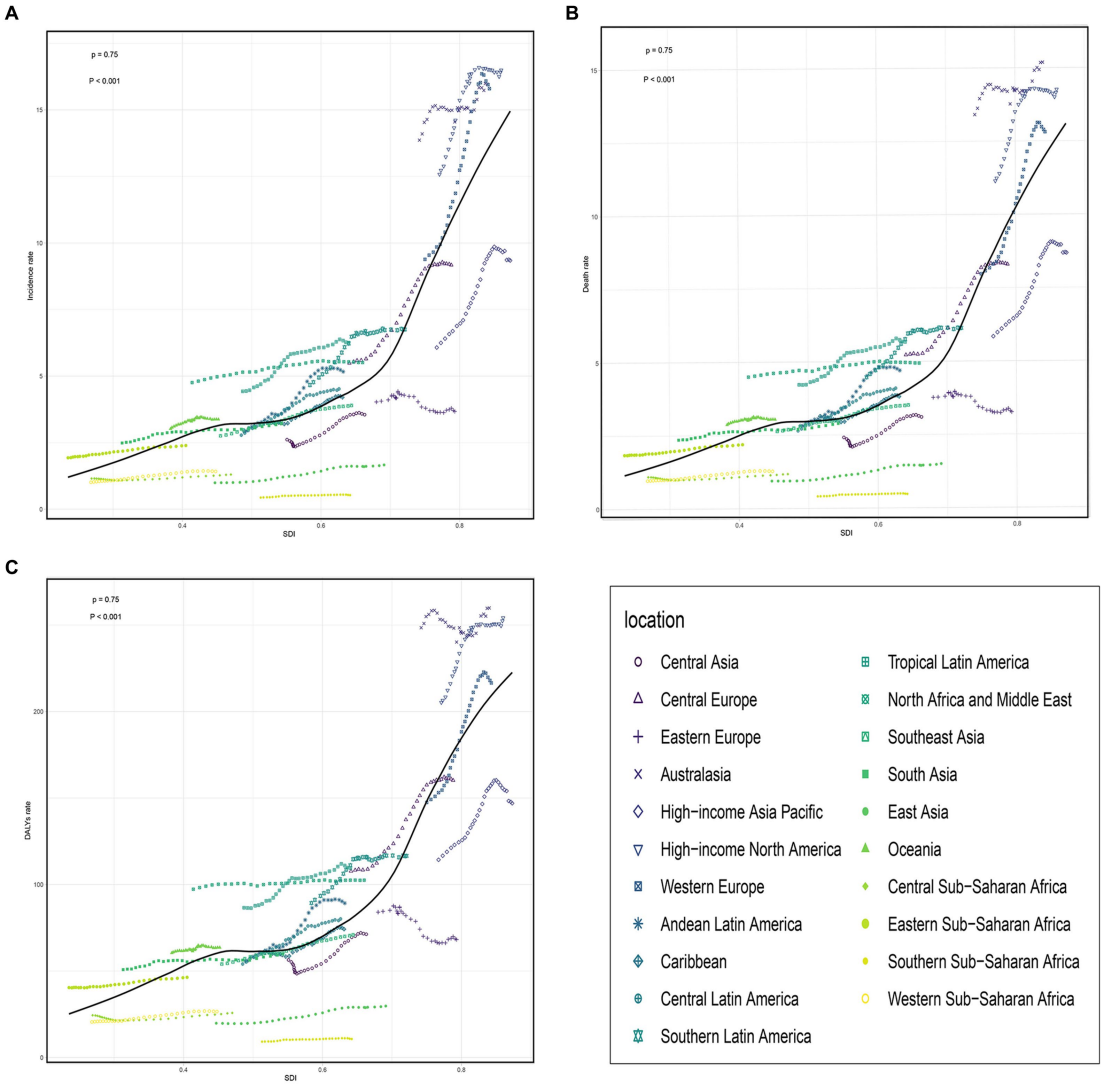
The change trends and correlation analyses of incidence rate and SDI from 1990 to 2019. **(A)** The change trends and correlation of incidence rate and SDI from 1990 to 2019 in 21 regions. **(B)** The change trends and correlation of death rate and SDI from 1990 to 2019 in 21 regions. **(C)** The change trends and correlation of DALYs rate and SDI from 1990 to 2019 in 21 regions. SDI, socio-demographic index.

In 2019, among 21 geographical regions, Western Europe had the highest number of incidence, deaths, and DALYs due to older AML with 17,298 (95% UI, 12,653 to 20,155) cases, 14,026 (95% UI, 10,543 to 15,277) deaths, and 237,146 (95% UI, 181,545 to 256,266) DALYs. Conversely, Oceania had the lowest numbers for incidence, deaths, and DALYs attributed to older AML in 2019, with 25 (95% UI, 18 to 35) cases, 22 (95% UI, 16 to 31) deaths, and 465 (95% UI, 329 to 657) DALYs. The highest incidence rate was observed in high-income North America, with 16.49 (95% UI, 13.46 to 19.05) cases per 100,000 population. Australia had the highest death rate and DALYs rate, with 15.19 (95% UI, 11.97 to 17.32) deaths per 100,000 population and 259.95 (95% UI, 206.72 to 294.4) DALYs per 100,000 population, respectively. In contrast, Sub-Saharan Africa (Southern region) had the lowest incidence rate, death rate, and DALYs rate, with 0.53 (95% UI, 0.41 to 0.71) cases per 100,000 population, 0.48(95% UI, 0.37 to 0.63) deaths per 100,000 population, and 10.69 (95% UI, 8.2 to 13.99) DALYs per 100,000 population (Table 1 and Figures 3A–C; Supplementary Figures S3A–C). From 1990 to 2019, the Andean Latin America region saw the most significant increases in incidence rate, death rate, and DALYs rate, with respective increases of 4.02 (95% UI, 2.06 to 6.10), 3.73 (95% UI, 1.88 to 5.68), and 3.54 (95% UI, 1.77 to 5.45). The corresponding EAPC values were also the highest, at 2.42 (95% CI, 2.22 to 2.62), 2.23 (95% CI, 2.03 to 2.43), and 2.03 (95% CI, 1.84 to 2.22).On the other hand, Eastern Europe had the lowest increases in incidence rate, death rate, and DALYs rate, with respective increases of 0.14 (95% UI, −0.05 to 0.34), 0.08 (95% UI, −0.11 to 0.28), and 0.02 (95% UI, −0.16 to 0.21). The corresponding EAPC values were also the lowest, at −0.61 (95% CI, −0.79 to −0.43), −0.75 (95% CI, −0.92 to −0.59), and −1.12 (95% CI, −1.33 to −0.92) (Table 1 and Figures 3A–C; Supplementary Figure S4).

### Burden of older people with AML in 204 countries.

**Figure 4:**
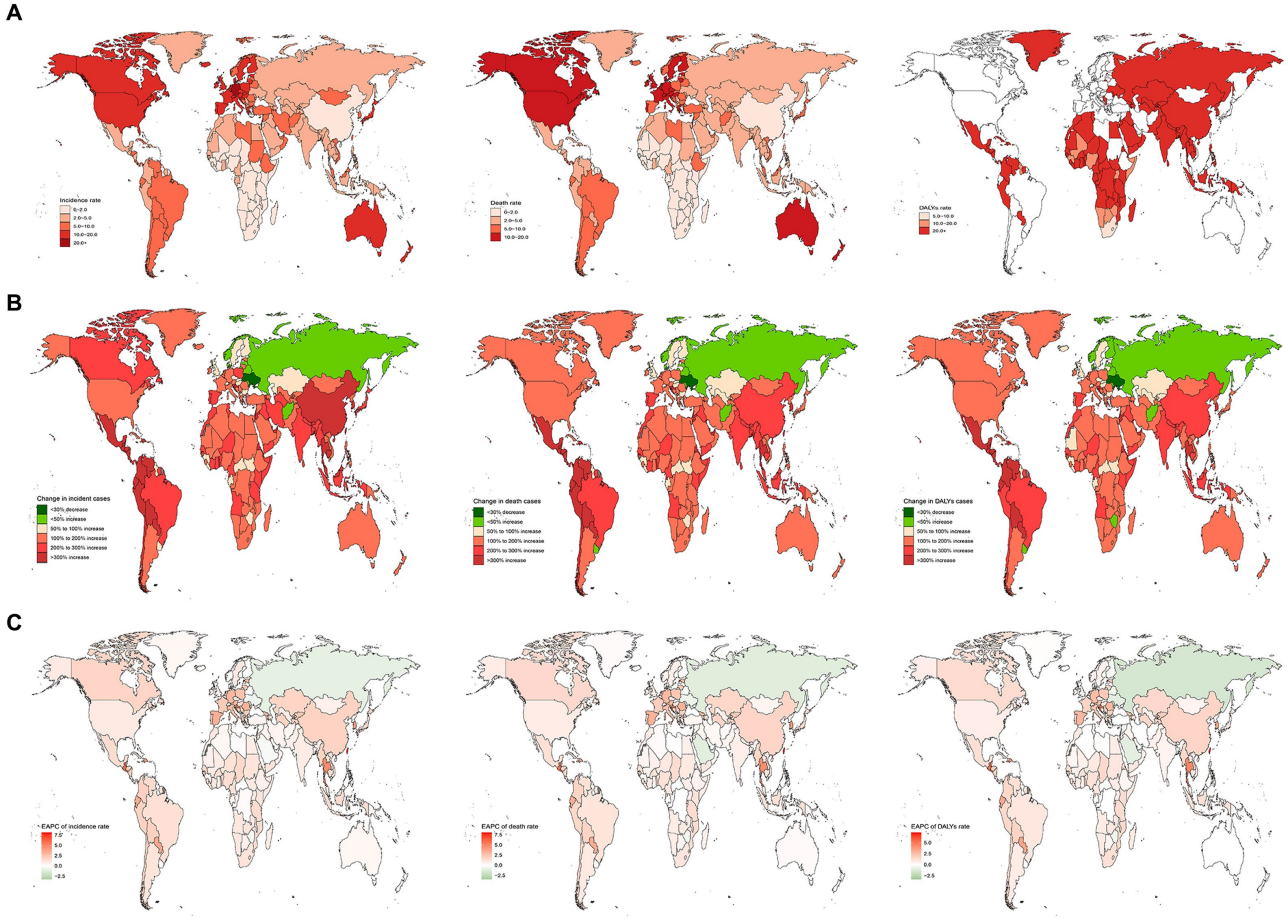
The nation burden of AML in the older people in 204 countries and territories. **(A)** The relative incident cases of AML in the older people between 1990 to 2019. **(B)** The relative changes of AML in the older people between 1990 to 2019. **(C)** The relative EAPCs of AML in the older people between 1990 to 2019. EAPC, estimated annual percentage.

**Figure 5:**
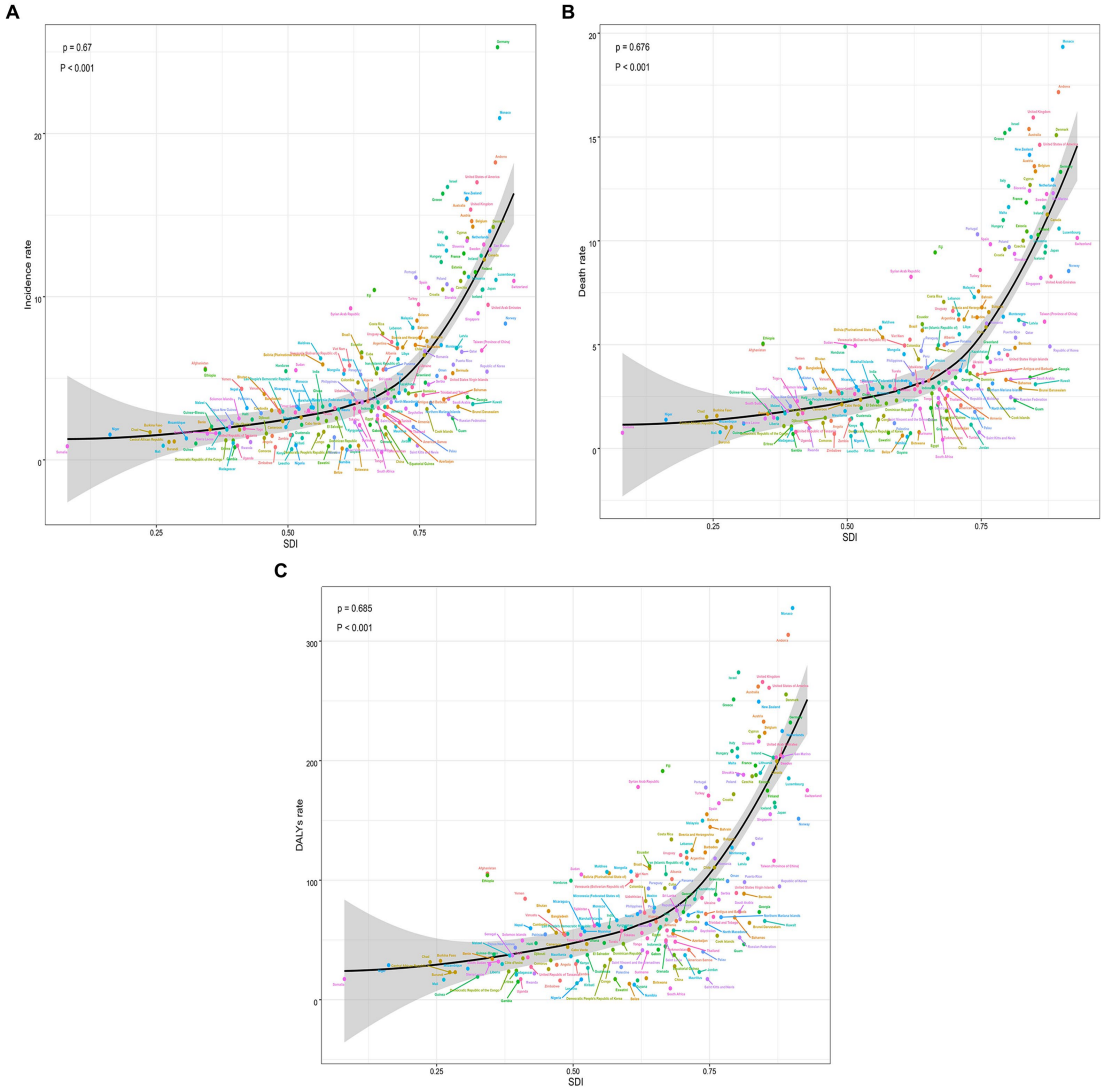
The trends in the change and correlation of incidence rate and SDI from 1990 to 2019 among 204 countries. **(A)** The change trends and correlation of incidence rate and SDI from 1990 to 2019 in 204 countries. **(B)** The change trends and correlation of death rate and SDI from 1990 to 2019 in 204 countries. **(C)** The change trends and correlation of DALYs rate and SDI from 1990 to 2019 in 204 countries. SDI, socio-demographic index.

In 2019, across 204 countries, the United States reported the highest numbers of older AML incidence, deaths, and DALYs in the older population, with 12,147 cases (95% UI, 9,810 to 14,138) cases, 12,147 (95% UI, 9,810 to 14,138) cases, and 186,339 (95% UI, 157,815 to 199,751) DALYs, respectively. Germany exhibited the highest incidence rate, at 25.29 (95% UI, 16.28 to 33.49) cases per 100,000 population. Monaco reported the highest mortality and DALYs rates, with 20.95 (95% UI, 13.06 to 31.27) deaths per 100,000 population and 327.67 (95% UI, 206.48 to 483.6), DALYs per 100,000 population, respectively. Conversely, South Africa had the lowest rates of incidence, mortality, and DALYs, at 0.47 (95% UI, 0.35 to 0.63) cases, 0.47 (95% UI, 0.35 to 0.63) deaths, and 9.47 (95% UI, 6.92 to 12.59) DALYs per 100,000 population, respectively. Between 1990 and 2019, Guatemala exhibited the most significant increases in incidence, mortality, and DALYs rates, with rises of 11.7 (95% UI, 5.39 to 19.87) cases, 11.7 (95% UI, 5.39 to 19.87) deaths, and 9.81 (95% UI, 4.56 to 16.3) DALYs per 100,000 population, respectively. Tokelau showed the most substantial decreases in incidence, mortality, and DALYs rates, with declines of 0.09 (95% UI, −0.4 to 0.41) cases, 0.09 (95% UI, −0.4 to 0.41) deaths, and 0.13 (95% UI, −0.44 to 0.37) DALYs per 100,000 population, respectively. Taiwan, China, demonstrated the highest EAPCs in incidence, mortality, and DALYs rates, at 8.14 (95% CI, 6.98 to 9.32), 8.14 (95% CI, 6.98 to 9.32), and 7.31 (95% CI, 6.23 to 8.4) respectively. The Northern Mariana Islands had the lowest EAPC in incidence rate at −3.29 (95% CI, −3.72 to −2.87), while Guam reported the lowest EAPCs in mortality and DALYs rates, at −3.07 (95% CI, −3.36 to −2.78) and −3.34 (95% CI, −3.55 to −3.13), respectively (Figures 4A–C, 5A–C and Supplementary Table S1).

### Correlation between EAPC and incidence rate/SDI, and relationship between incidence rate, mortality rate, DALYs rate, and SDI

**Figure 6:**
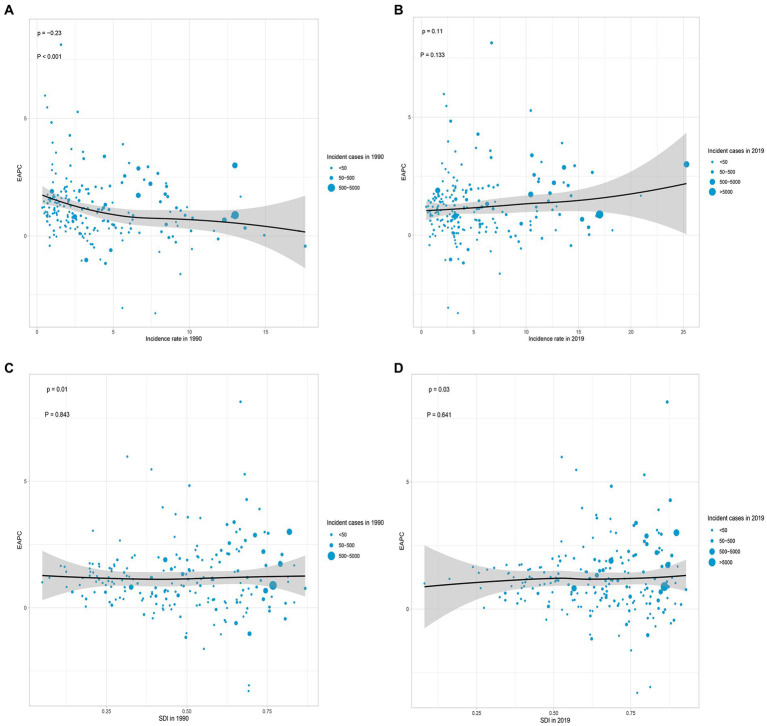
Correlation analyses of EAPCs – Incidence rate (1990) and EAPCs – SDI (2019) in 204 countries or territories. **(A)** Correlation between EAPC of incidence rate and incidence rate of 1990. **(B)** Correlation between EAPC of incidence rate and incidence rate of 2019. **(C)** Correlation between EAPC of incidence rate and SDI of 1990. **(D)** Correlation between EAPC of incidence rate and SDI of 2019. The size of each circle represents the quantity of AML in older people in each country or territory. EAPC, estimated annual percentage; SDI, socio-demographic index.

According to the data, there was a weak negative correlation (−0.23) between EAPC and incidence rate in 1990, indicating a slight inverse relationship (Figure 6A). However, in 2019, no significant correlation was observed between EAPC and incidence rate. Likewise, no significant correlation was found between EAPC and SDI in both 1990 and 2019 (Figures 6B–D). Furthermore, correlation analyses were conducted between SDI and incidence rate, mortality rate, and DALYs rate across 21 global regions. The calculated correlation coefficients were all 0.75, indicating a strong positive correlation between SDI and these health indicators across different regions (Figures 3A–C). Additionally, the correlation between SDI and incidence rate, mortality rate, and DALYs rate in 204 countries worldwide were examined. The correlation coefficients were 0.67, 0.676, and 0.685, respectively (Figures 5A–C). These findings highlight a robust positive association between SDI and the examined health indicators across various countries. In conclusion, the data suggests a strong positive correlation between SDI and incidence rate, mortality rate, and DALYs rate in global regions and countries. However, the correlation between EAPC and incidence rate/SDI was not significant, potentially indicating the influence of other factors.

### Risk factors for AML in older people

**Figure 7:**
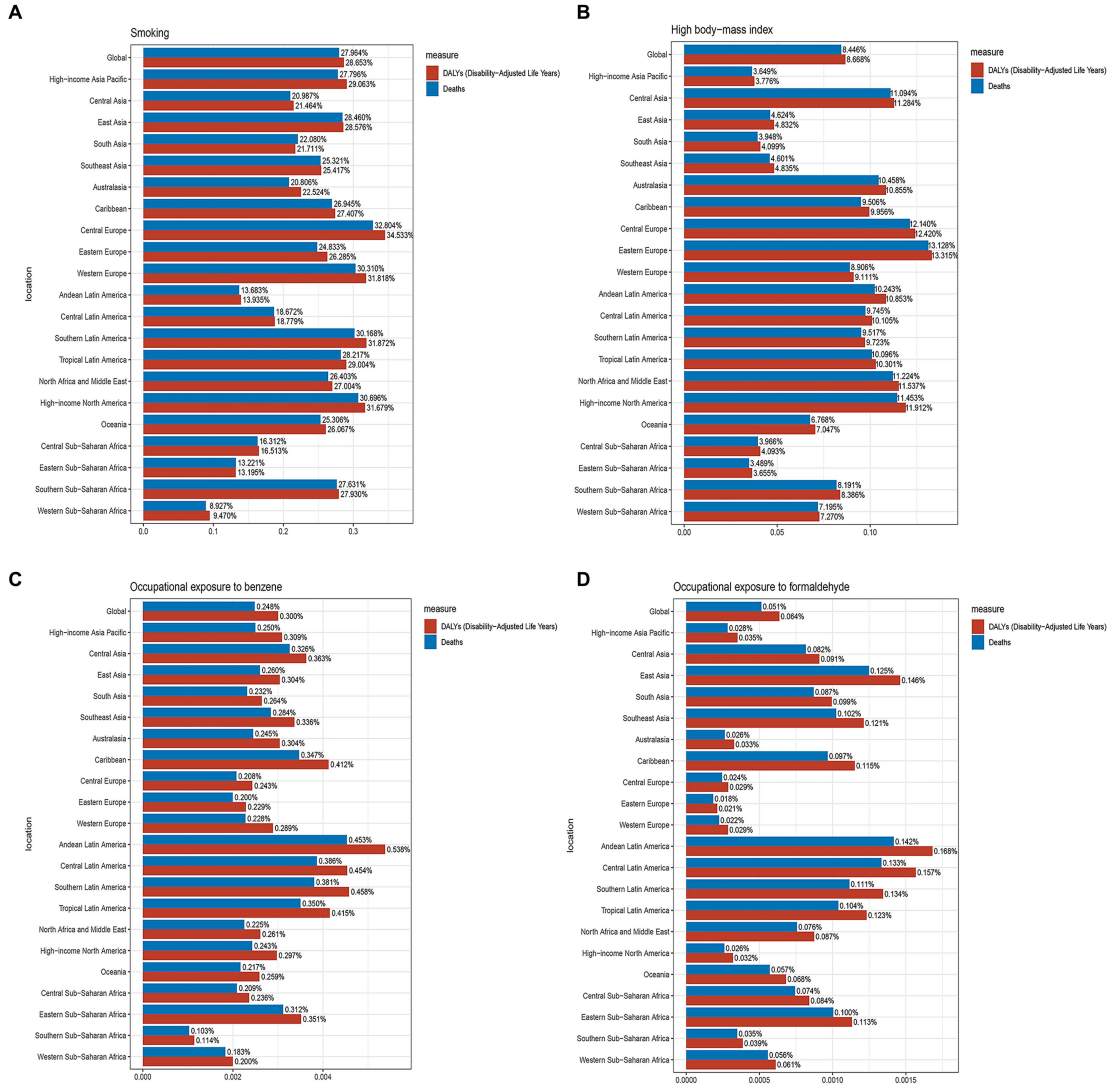
The proportions of four risk factors contributing to older people with AML deaths and DALYs vary across the 21 global regions. **(A)** Proportion of smoking-associated risk. **(B)** Proportion of high body mass index-related risk. **(C)** Proportion of benzene occupational exposure-related risk. **(D)** Proportion of formaldehyde occupational exposure-related risk.

According to data from the GBD database, the risk factors that contributed to mortality from AML in older people were smoking, high body mass index (BMI), occupational exposure to benzene, and formaldehyde. In 2019, smoking was responsible for an estimated 15,003 (95% UI, 8,420 to 21,826) deaths related to AML in older people globally. Among the 21 geographical regions considered, Central Europe had the highest proportion of deaths and DALYs attributed to smoking, accounting for 32.804 and 34.533%, respectively. Conversely, Southern Sub-Saharan Africa had the lowest percentages, with only 8.927% of deaths and 9.470% of DALYs associated with smoking (Figure 7A). High BMI was responsible for approximately 4,525 (95% UI, 2,202 to 7,603) deaths related to AML in older people globally in 2019. Eastern Europe had the highest proportion of deaths and DALYs attributed to high BMI among the geographical regions, accounting for 13.128 and 13.315%, respectively. Conversely, Eastern Sub-Saharan Africa had the lowest percentages, with only 3.489% of deaths and 3.655% of DALYs associated with high BMI (Figure 7B). Occupational exposure to benzene was estimated to contribute to about 133 (95% UI, 38 to 223) deaths related to AML in older people globally. Among the regions considered, Andean Latin America had the highest proportion of deaths and DALYs attributed to occupational exposure to benzene, accounting for 0.453 and 0.538%, respectively. On the other hand, Southern Sub-Saharan Africa had the lowest percentages, with only 0.103% of deaths and 0.114% of DALYs associated with this occupational exposure (Figure 7C). Occupational exposure to formaldehyde was found to contribute to approximately 27 (95% UI, 20 to 36) deaths related to AML in older people globally. Among the geographical regions, Andean Latin America had the highest percentage of deaths and DALYs attributed to occupational exposure to formaldehyde, accounting for 0.142 and 0.168%, respectively. Conversely, Eastern Europe had the lowest percentages, with only 0.018% of deaths and 0.021% of DALYs associated with this occupational exposure (Figure 7D). These findings underscore the importance of addressing and reducing the impact of risk factors such as smoking, high BMI, occupational exposure to benzene, and formaldehyde in order to mitigate the burden of AML in older people.

## Discussion

In this study, it was found that from 1990 to 2019, the incidence, mortality, and DALYs of AML among older adults globally showed a continuous upward trend. This upward trend was largely driven by population aging, highlighting the significant impact of this common older adult disease. With increasing life expectancy and changes in population demographics, the prevalence of diseases among older individuals has continued to rise, posing a significant global challenge. Furthermore, our study also investigated the relationship between the incidence, mortality, and DALYs of AML in older people and the SDI. We found a significant positive correlation between SDI and the incidence, mortality, and DALYs rates of AML in older people, both globally and within different regions and countries. Our findings indicate that as the level of social development improves, there is a corresponding increase in the incidence, mortality, and DALYs rates of AML in older people. This could be attributed to better healthcare measures and diagnostic technologies resulting from improved social development, leading to increased diagnoses and consequently higher incidence and mortality rates among older people. Additionally, higher levels of socio-economic development also imply an increase in the older population, thereby raising the risk of AML in older people. Despite similar SDI levels, there exist variations in the incidence, mortality, and DALYs rates of AML in older people among countries, which may be influenced by multiple factors. Factors such as variations in the distribution of the older population, healthcare resources, and environmental exposures across different regions could contribute to differences in the incidence, mortality, and DALYs rates. Moreover, variations in population structure, health status, nutrition, and the accumulation of chronic diseases among older people in different countries may also impact these rates. Additionally, differences in prevention, screening, diagnosis, and treatment strategies for AML, as well as disparities in the quality of healthcare systems, disease surveillance and management strategies, and the allocation and accessibility of healthcare resources, can influence the incidence, mortality, and DALYs rates. Therefore, these factors need to be considered holistically to develop more effective strategies for the prevention and treatment of AML, aiming to reduce the burden of incidence, mortality, and DALYs.

AML is a complex, multifactorial disease involving the interaction of various factors including genetics, environment, and lifestyle (21, 22). Genetic factors play a crucial role in the development of AML. Certain genetic mutations and abnormalities are associated with an increased risk of AML, such as chromosomal abnormalities (e.g., *t*(8; 21), *t*(15;17)) and gene mutations (e.g., FLT3, NPM1, DNMT3A). These mutations and abnormalities can disrupt the proliferation and differentiation of hematopoietic stem cells, promoting the progression of AML (23–25). Environmental factors are also associated with the risk of AML. Certain substance exposures, such as chemicals, certain medications, ionizing radiation, are considered potential risk factors for AML. Additionally, carcinogenic substances in occupational and living environments may also be linked to an increased risk of AML (26, 27). Lifestyle factors may also play a role in the development of AML. Long-term smoking, high body mass index, unhealthy diet, and lack of physical activity have been associated with an elevated risk of AML (28–30). Chemotherapy is a widely used treatment for leukemia. However, it is important to note that it may also contribute to the development of older AML in certain cases (31). In older patients with AML, the signs and symptoms are often nonspecific. Non-specific signs and symptoms of AML may include fatigue, anemia, easy bruising and bleeding, bone pain, as well as fever and infection (32–34). However, these symptoms do not specifically indicate AML, as they can also be related to many other diseases. Therefore, diagnosing AML correctly can be particularly challenging for older patients, especially those who already have other health issues. Early detection and diagnosis are crucial to initiate treatment promptly and improve patient prognosis.

Our study also had certain limitations. The data on AML in older individuals collected from the GBD database may not have comprehensively covered specific populations such as ethnic minorities, immigrant groups, or certain occupational cohorts. The quality and comprehensiveness of these data may have varied by country, with high SDI regions often associated with higher-quality health data due to better data collection and processing capabilities, while low SDI regions may have faced challenges in data collection and quality control. Additionally, our analyses heavily relied on statistical models and estimation methods, which could have introduced biases or uncertainties, especially in the absence of high-quality data.

In summary, there was a significant positive correlation between the incidence, mortality, and DALYs rates of AML in older individuals and SDI. As the global SDI continued to rise, the importance of AML as a health issue among the older population became increasingly pronounced, especially in high SDI regions. We should have paid closer attention to this disease and promptly implemented preventive and control measures. It was worth noting that, globally in 2019, smoking, high body mass index, occupational exposure to benzene, and formaldehyde were identified as major risk factors for AML-related deaths among older adults. These findings were crucial for the development of targeted prevention strategies and could have guided public health policies and interventions on a global scale.

## Data availability statement

The original contributions presented in the study are included in the article/Supplementary material, further inquiries can be directed to the corresponding author.

## Ethics statement

Ethical approval was not required for the study involving humans in accordance with the local legislation and institutional requirements. Written informed consent to participate in this study was not required from the participants or the participants’ legal guardians/next of kin in accordance with the national legislation and the institutional requirements.

## Author contributions

PC: Writing – original draft, Writing – review & editing. XL: Writing – review & editing. YZ: Formal analysis, Writing – review & editing. YH: Data curation, Writing – review & editing. JG: Formal analysis, Writing – review & editing. HW: Funding acquisition, Writing – review & editing.

## Footnotes

^1^http://ghdx.healthdata.org/gbd-results-tool
