# Supplementary material for: Global, national, and regional burden of acute myeloid leukemia among 60–89 years-old individuals: insights from a study covering the period 1990 to 2019
Source: Front Public Health. 2024 Jan 11;11:1329529. doi: 10.3389/fpubh.2023.1329529 (PMC10808630; doi:10.3389/fpubh.2023.1329529)
Supplement: Supplementary file 1 [file Table_1.DOCX]

**Supplementary Table 1. The incidence, mortality, and disability-adjusted life years (DALYs) in elderly patients with acute myeloid leukemia in 204 countries between 1990 and 2019.**

| location | Incident cases in 1990 | Incidence rate in 1990 | Incident cases in 2019 | Incidence rate in 2019 | Cases change | EAPC |
| --- | --- | --- | --- | --- | --- | --- |
| Afghanistan | 45  (23 to 78) | 5.17  (2.71 to 9.01) | 60  (30 to 100) | 5.59  (2.74 to 9.28) | 0.35  (-0.17 to 1.14) | 0.35  (0.24 to 0.46) |
| Albania | 13  (10 to 21) | 5.51  (4.07 to 8.6) | 31  (20 to 52) | 5.53  (3.48 to 9.25) | 1.38  (0.61 to 2.56) | 0.12  (0.01 to 0.22) |
| Algeria | 48  (32 to 67) | 3.3  (2.21 to 4.56) | 141  (92 to 194) | 3.58  (2.34 to 4.93) | 1.91  (0.94 to 3.58) | 0.35  (0.26 to 0.44) |
| American Samoa | 0  (0 to 0) | 2.93  (2.1 to 3.79) | 0  (0 to 0) | 2.29  (1.69 to 3.09) | 0.75  (0.16 to 1.74) | -1.05  (-1.26 to -0.84) |
| Andorra | 1  (1 to 2) | 17.21  (11.68 to 24.14) | 3  (2 to 4) | 18.23  (12.18 to 24.29) | 1.48  (0.61 to 2.71) | 0.21  (0.1 to 0.32) |
| Angola | 5  (3 to 8) | 1.15  (0.69 to 1.86) | 17  (10 to 26) | 1.47  (0.88 to 2.24) | 2.62  (1.16 to 5.12) | 0.98  (0.89 to 1.07) |
| Antigua and Barbuda | 0  (0 to 0) | 2.44  (1.91 to 3.13) | 0  (0 to 1) | 3.96  (3.01 to 5.12) | 1.96  (1.12 to 3.21) | 1.92  (1.73 to 2.11) |
| Argentina | 194  (160 to 233) | 4.72  (3.91 to 5.66) | 463  (347 to 602) | 6.85  (5.13 to 8.9) | 1.39  (0.76 to 2.24) | 1.12  (0.83 to 1.41) |
| Armenia | 6  (4 to 8) | 1.79  (1.32 to 2.43) | 18  (11 to 24) | 3.25  (1.94 to 4.37) | 2.04  (0.77 to 3.59) | 2  (1.72 to 2.28) |
| Australia | 346  (295 to 378) | 13.65  (11.64 to 14.93) | 829  (591 to 1085) | 15.96  (11.37 to 20.9) | 1.4  (0.81 to 2.12) | 0.34  (0.23 to 0.45) |
| Austria | 111  (99 to 138) | 7.28  (6.46 to 8.99) | 311  (202 to 400) | 14.63  (9.49 to 18.79) | 1.79  (0.68 to 2.67) | 2.94  (2.64 to 3.25) |
| Azerbaijan | 11  (7 to 16) | 1.88  (1.29 to 2.87) | 29  (19 to 45) | 2.74  (1.83 to 4.19) | 1.76  (0.87 to 3.05) | 1.61  (1.45 to 1.76) |
| Bahamas | 1  (0 to 1) | 3.21  (2.74 to 3.99) | 2  (1 to 2) | 3.71  (2.95 to 4.66) | 2.04  (1.32 to 2.95) | 0.76  (0.66 to 0.86) |
| Bahrain | 2  (1 to 2) | 9.41  (5.72 to 13.77) | 8  (5 to 11) | 7.49  (4.97 to 10.34) | 3.64  (1.95 to 6.62) | -1.62  (-2.03 to -1.21) |
| Bangladesh | 147  (93 to 214) | 2.81  (1.78 to 4.1) | 472  (286 to 743) | 3.02  (1.83 to 4.75) | 2.22  (1.01 to 4.03) | 0.1  (0 to 0.21) |
| Barbados | 2  (2 to 2) | 4.96  (4.25 to 6.11) | 4  (4 to 5) | 7.04  (5.68 to 8.48) | 1.41  (0.88 to 2.04) | 1.42  (1.17 to 1.67) |
| Belarus | 145  (108 to 173) | 8.65  (6.43 to 10.31) | 177  (122 to 236) | 8.53  (5.88 to 11.38) | 0.22  (-0.11 to 0.67) | -0.07  (-0.22 to 0.08) |
| Belgium | 172  (147 to 225) | 8.58  (7.35 to 11.27) | 395  (286 to 510) | 14.29  (10.35 to 18.47) | 1.3  (0.48 to 2.15) | 1.67  (1.42 to 1.93) |
| Belize | 0  (0 to 0) | 0.45  (0.33 to 0.64) | 0  (0 to 0) | 0.7  (0.53 to 0.92) | 3.25  (1.95 to 5.33) | 1.47  (1.27 to 1.67) |
| Benin | 3  (2 to 4) | 1.3  (0.75 to 1.86) | 10  (5 to 14) | 1.86  (0.89 to 2.73) | 2.22  (0.98 to 4.26) | 1.39  (1.26 to 1.53) |
| Bermuda | 0  (0 to 1) | 5.61  (4.4 to 7.08) | 1  (1 to 1) | 5.31  (4.14 to 6.68) | 1.03  (0.5 to 1.76) | -0.19  (-0.27 to -0.11) |
| Bhutan | 1  (0 to 1) | 2.58  (1.51 to 4.1) | 3  (2 to 5) | 4.04  (2.43 to 6.84) | 2.91  (1.43 to 5.42) | 1.63  (1.49 to 1.77) |
| Bolivia (Plurinational State of) | 12  (8 to 18) | 3.35  (2.2 to 4.97) | 62  (41 to 88) | 5.85  (3.83 to 8.28) | 4.03  (2.26 to 6.79) | 1.99  (1.86 to 2.13) |
| Bosnia and Herzegovina | 20  (13 to 35) | 4.25  (2.82 to 7.34) | 55  (38 to 73) | 6.89  (4.72 to 9.13) | 1.7  (0.38 to 3.56) | 2.03  (1.77 to 2.29) |
| Botswana | 0  (0 to 1) | 0.66  (0.37 to 1.11) | 1  (1 to 2) | 0.87  (0.42 to 1.51) | 1.99  (0.64 to 4.21) | 0.62  (0.39 to 0.86) |
| Brazil | 466  (417 to 533) | 4.48  (4.02 to 5.13) | 1809  (1452 to 1982) | 6.28  (5.04 to 6.88) | 2.88  (2.22 to 3.25) | 1.33  (1.18 to 1.48) |
| Brunei Darussalam | 0  (0 to 1) | 3.51  (2.15 to 5.08) | 1  (1 to 1) | 3.26  (2.42 to 4.43) | 2.07  (0.95 to 4.28) | -0.35  (-0.62 to -0.07) |
| Bulgaria | 80  (63 to 97) | 4.79  (3.74 to 5.79) | 141  (107 to 182) | 7.29  (5.56 to 9.43) | 0.75  (0.28 to 1.39) | 2.12  (1.83 to 2.42) |
| Burkina Faso | 6  (3 to 10) | 1.2  (0.65 to 1.86) | 17  (8 to 27) | 1.76  (0.79 to 2.69) | 1.83  (0.74 to 3.72) | 1.42  (1.23 to 1.62) |
| Burundi | 2  (1 to 3) | 0.76  (0.47 to 1.28) | 6  (3 to 9) | 1.13  (0.62 to 1.88) | 1.71  (0.32 to 3.79) | 1.55  (1.46 to 1.64) |
| Cabo Verde | 0  (0 to 1) | 1.23  (0.65 to 1.78) | 1  (1 to 2) | 2.59  (1.04 to 3.89) | 2.52  (1.07 to 4.64) | 2.66  (2.09 to 3.23) |
| Cambodia | 10  (6 to 17) | 2.04  (1.2 to 3.43) | 42  (27 to 68) | 3.03  (1.94 to 4.97) | 3.07  (1.63 to 5.32) | 1.44  (1.39 to 1.49) |
| Cameroon | 9  (5 to 13) | 1.77  (1.02 to 2.7) | 30  (13 to 46) | 2.37  (1.04 to 3.61) | 2.43  (0.98 to 4.93) | 1.26  (1.15 to 1.37) |
| Canada | 349  (309 to 456) | 8.41  (7.45 to 10.99) | 1073  (812 to 1400) | 12.28  (9.28 to 16.02) | 2.08  (1.18 to 3.17) | 1.78  (1.55 to 2) |
| Central African Republic | 1  (1 to 2) | 1.11  (0.66 to 1.79) | 2  (1 to 4) | 1.1  (0.58 to 1.95) | 0.7  (-0.06 to 2.08) | 0.05  (-0.01 to 0.11) |
| Chad | 4  (2 to 6) | 1.15  (0.63 to 1.75) | 10  (5 to 15) | 1.69  (0.79 to 2.48) | 1.7  (0.7 to 3.39) | 1.65  (1.52 to 1.79) |
| Chile | 46  (39 to 62) | 3.85  (3.21 to 5.16) | 192  (145 to 250) | 6.42  (4.85 to 8.35) | 3.15  (1.9 to 4.7) | 2.09  (1.87 to 2.31) |
| China | 980  (725 to 1362) | 0.97  (0.72 to 1.35) | 3975  (3180 to 4950) | 1.56  (1.25 to 1.95) | 3.05  (1.8 to 4.95) | 1.9  (1.75 to 2.05) |
| Colombia | 56  (47 to 77) | 2.79  (2.38 to 3.85) | 303  (218 to 399) | 4.74  (3.42 to 6.25) | 4.46  (2.53 to 6.75) | 1.95  (1.75 to 2.14) |
| Comoros | 0  (0 to 0) | 1.03  (0.55 to 1.92) | 1  (0 to 1) | 1.34  (0.84 to 2.08) | 1.78  (0.55 to 4.02) | 0.95  (0.89 to 1) |
| Congo | 2  (1 to 3) | 1.52  (0.96 to 2.44) | 5  (3 to 7) | 1.72  (1.07 to 2.49) | 1.48  (0.49 to 3.4) | 0.36  (0.25 to 0.48) |
| Cook Islands | 0  (0 to 0) | 2.99  (2.19 to 3.93) | 0  (0 to 0) | 3.11  (2.33 to 4.07) | 1.18  (0.51 to 2.21) | -0.01  (-0.07 to 0.05) |
| Costa Rica | 12  (10 to 15) | 6.01  (4.79 to 7.26) | 48  (35 to 65) | 7.74  (5.68 to 10.36) | 2.93  (1.8 to 4.45) | 0.99  (0.85 to 1.12) |
| C么te d'Ivoire | 6  (3 to 9) | 1.4  (0.81 to 2.16) | 20  (10 to 31) | 1.85  (0.88 to 2.82) | 2.48  (1.14 to 4.95) | 1.06  (1 to 1.12) |
| Croatia | 22  (14 to 53) | 2.67  (1.75 to 6.45) | 119  (61 to 163) | 10.44  (5.33 to 14.22) | 4.45  (0.35 to 8.97) | 5.28  (4.65 to 5.91) |
| Cuba | 54  (46 to 71) | 4.26  (3.6 to 5.58) | 120  (93 to 156) | 5.3  (4.13 to 6.88) | 1.22  (0.72 to 1.81) | 1.11  (0.95 to 1.28) |
| Cyprus | 9  (7 to 12) | 8.47  (6.4 to 10.84) | 34  (16 to 46) | 13.58  (6.28 to 18.28) | 2.77  (0.88 to 4.58) | 2.24  (1.94 to 2.54) |
| Czechia | 166  (146 to 205) | 9.15  (8.05 to 11.3) | 299  (222 to 378) | 10.96  (8.15 to 13.88) | 0.8  (0.23 to 1.34) | 1.2  (0.96 to 1.43) |
| Democratic People's Republic of Korea | 24  (15 to 37) | 1.35  (0.84 to 2.11) | 59  (34 to 88) | 1.55  (0.89 to 2.3) | 1.48  (0.54 to 3.01) | 0.67  (0.58 to 0.76) |
| Democratic Republic of the Congo | 20  (12 to 31) | 1.11  (0.68 to 1.73) | 45  (23 to 78) | 1.2  (0.61 to 2.1) | 1.27  (0.16 to 2.94) | 0.22  (-0.01 to 0.45) |
| Denmark | 180  (151 to 198) | 17.6  (14.72 to 19.28) | 204  (153 to 261) | 14.27  (10.69 to 18.2) | 0.13  (-0.12 to 0.43) | -0.44  (-0.67 to -0.2) |
| Djibouti | 0  (0 to 0) | 1.04  (0.53 to 2.02) | 1  (1 to 2) | 1.62  (0.95 to 2.74) | 5.93  (3.23 to 11.46) | 1.65  (1.57 to 1.72) |
| Dominica | 0  (0 to 0) | 3.06  (2.16 to 4.33) | 0  (0 to 1) | 4.07  (2.79 to 5.57) | 0.58  (0.06 to 1.44) | 1.19  (1.06 to 1.33) |
| Dominican Republic | 4  (3 to 8) | 1.02  (0.69 to 1.82) | 28  (18 to 40) | 2.56  (1.62 to 3.68) | 5.37  (2.14 to 10.48) | 3.97  (3.73 to 4.21) |
| Ecuador | 19  (14 to 28) | 3.02  (2.27 to 4.57) | 120  (75 to 160) | 6.59  (4.11 to 8.79) | 5.46  (2.07 to 9.02) | 3.58  (3.22 to 3.94) |
| Egypt | 77  (47 to 130) | 2.34  (1.44 to 3.96) | 217  (122 to 374) | 2.99  (1.68 to 5.16) | 1.83  (0.72 to 3.37) | 0.79  (0.62 to 0.96) |
| El Salvador | 2  (2 to 4) | 0.67  (0.5 to 1.09) | 17  (8 to 24) | 2.39  (1.06 to 3.43) | 6.35  (1.72 to 11.45) | 5.47  (4.82 to 6.12) |
| Equatorial Guinea | 0  (0 to 0) | 1.01  (0.56 to 1.69) | 1  (1 to 2) | 1.98  (1.01 to 3.51) | 3.55  (1.1 to 8.92) | 3.05  (2.77 to 3.32) |
| Eritrea | 1  (0 to 1) | 0.69  (0.39 to 1.14) | 3  (2 to 5) | 1.19  (0.76 to 1.76) | 3.88  (1.89 to 7.63) | 1.76  (1.5 to 2.01) |
| Estonia | 16  (11 to 33) | 6.04  (4.19 to 12.43) | 38  (29 to 50) | 11.46  (8.67 to 14.85) | 1.41  (0.15 to 2.84) | 3.1  (2.45 to 3.76) |
| Eswatini | 0  (0 to 0) | 0.69  (0.4 to 1.11) | 1  (0 to 1) | 0.83  (0.38 to 1.33) | 1.52  (0.46 to 3.44) | 0.62  (0.36 to 0.87) |
| Ethiopia | 102  (44 to 181) | 4.65  (2 to 8.29) | 249  (109 to 384) | 5.53  (2.43 to 8.53) | 1.44  (0.58 to 2.95) | 0.63  (0.58 to 0.68) |
| Fiji | 3  (2 to 4) | 9.12  (6.73 to 12.04) | 9  (7 to 12) | 10.41  (7.67 to 13.68) | 1.68  (0.81 to 3) | 0.59  (0.47 to 0.71) |
| Finland | 109  (96 to 123) | 11.89  (10.5 to 13.36) | 177  (134 to 229) | 11.51  (8.72 to 14.88) | 0.62  (0.23 to 1.14) | -0.13  (-0.19 to -0.06) |
| France | 790  (707 to 974) | 7.44  (6.65 to 9.18) | 2080  (1410 to 2729) | 12.65  (8.58 to 16.6) | 1.63  (0.66 to 2.59) | 2.22  (2 to 2.45) |
| Gabon | 1  (1 to 2) | 1.84  (0.96 to 3.52) | 2  (1 to 4) | 2.15  (1.19 to 3.4) | 1  (0.09 to 2.89) | 0.44  (0.38 to 0.5) |
| Gambia | 0  (0 to 0) | 0.56  (0.33 to 0.86) | 1  (1 to 1) | 0.77  (0.5 to 1.14) | 2.8  (1.16 to 6.05) | 1.03  (0.82 to 1.25) |
| Georgia | 17  (12 to 30) | 2.19  (1.53 to 3.78) | 30  (17 to 40) | 3.84  (2.26 to 5.16) | 0.7  (-0.35 to 1.78) | 2.99  (1.72 to 4.28) |
| Germany | 2074  (1759 to 2412) | 12.99  (11.01 to 15.1) | 5818  (3745 to 7703) | 25.29  (16.28 to 33.49) | 1.8  (0.87 to 2.75) | 3  (2.78 to 3.23) |
| Ghana | 14  (9 to 21) | 2.06  (1.33 to 3.06) | 45  (32 to 62) | 2.53  (1.82 to 3.51) | 2.17  (1.01 to 4.3) | 0.58  (0.5 to 0.67) |
| Greece | 157  (138 to 178) | 7.93  (6.98 to 8.99) | 460  (284 to 605) | 16.32  (10.09 to 21.45) | 1.93  (0.82 to 2.85) | 2.66  (2.51 to 2.81) |
| Greenland | 0  (0 to 0) | 4.72  (3.57 to 6.03) | 0  (0 to 1) | 4.73  (3.43 to 6.81) | 1.33  (0.61 to 2.19) | 0.32  (0.16 to 0.48) |
| Grenada | 0  (0 to 0) | 1.6  (1.2 to 2.05) | 0  (0 to 0) | 2.37  (1.84 to 3.27) | 1.13  (0.51 to 2.06) | 1.61  (1.44 to 1.77) |
| Guam | 0  (0 to 1) | 5.6  (3.83 to 7.21) | 1  (0 to 1) | 2.55  (1.55 to 5.07) | 0.2  (-0.33 to 2.11) | -3.07  (-3.36 to -2.78) |
| Guatemala | 2  (2 to 4) | 0.53  (0.37 to 0.88) | 28  (20 to 36) | 2.15  (1.49 to 2.78) | 11.7  (5.39 to 19.87) | 5.98  (5.52 to 6.43) |
| Guinea | 3  (2 to 5) | 0.83  (0.54 to 1.25) | 6  (4 to 9) | 1  (0.65 to 1.53) | 0.86  (0.12 to 2.19) | 0.47  (0.37 to 0.57) |
| Guinea-Bissau | 1  (0 to 1) | 1.43  (0.81 to 2.15) | 2  (1 to 2) | 2.04  (0.88 to 3.14) | 1.36  (0.36 to 2.92) | 1.42  (1.33 to 1.52) |
| Guyana | 0  (0 to 0) | 0.83  (0.57 to 1.1) | 1  (0 to 1) | 0.83  (0.55 to 1.22) | 0.68  (0.01 to 1.95) | -0.06  (-0.24 to 0.11) |
| Haiti | 8  (4 to 13) | 2.02  (1.06 to 3.52) | 19  (9 to 33) | 2.47  (1.19 to 4.28) | 1.55  (0.6 to 3.02) | 0.81  (0.75 to 0.87) |
| Honduras | 8  (5 to 11) | 3.22  (2.21 to 4.5) | 38  (26 to 54) | 5.44  (3.65 to 7.58) | 4.11  (2.22 to 6.94) | 1.98  (1.88 to 2.09) |
| Hungary | 158  (140 to 179) | 8.1  (7.21 to 9.21) | 309  (230 to 386) | 12.13  (9 to 15.12) | 0.96  (0.45 to 1.44) | 1.46  (1.06 to 1.85) |
| Iceland | 3  (3 to 4) | 9.22  (7.76 to 10.67) | 7  (6 to 9) | 10.43  (8.16 to 13.68) | 1.16  (0.65 to 1.91) | 0.43  (0.3 to 0.57) |
| India | 1234  (943 to 1570) | 2.48  (1.89 to 3.15) | 4462  (3675 to 5462) | 3.26  (2.69 to 3.99) | 2.62  (1.73 to 3.85) | 0.82  (0.72 to 0.92) |
| Indonesia | 224  (171 to 378) | 2.08  (1.59 to 3.51) | 804  (551 to 1327) | 3.29  (2.25 to 5.43) | 2.59  (1.68 to 3.84) | 1.53  (1.44 to 1.63) |
| Iran (Islamic Republic of) | 154  (120 to 206) | 5.04  (3.93 to 6.74) | 471  (382 to 672) | 5.6  (4.55 to 8) | 2.05  (1.37 to 2.96) | 0.46  (0.27 to 0.65) |
| Iraq | 26  (16 to 45) | 2.98  (1.88 to 5.1) | 88  (57 to 141) | 3.53  (2.28 to 5.68) | 2.37  (1.2 to 4.4) | 0.55  (0.41 to 0.69) |
| Ireland | 50  (43 to 62) | 9.43  (8.18 to 11.7) | 117  (85 to 150) | 12.51  (9.12 to 16.13) | 1.33  (0.61 to 2.16) | 1.08  (0.94 to 1.21) |
| Israel | 81  (65 to 93) | 12.81  (10.37 to 14.77) | 242  (164 to 318) | 16.73  (11.32 to 21.92) | 2.01  (1.21 to 3.05) | 0.82  (0.58 to 1.06) |
| Italy | 774  (684 to 1172) | 6.65  (5.88 to 10.07) | 2294  (1687 to 2766) | 13.62  (10.02 to 16.42) | 1.96  (0.72 to 2.85) | 2.87  (2.52 to 3.23) |
| Jamaica | 3  (2 to 4) | 1.24  (0.95 to 1.7) | 12  (8 to 15) | 3.25  (2.34 to 4.29) | 3.13  (1.61 to 5.06) | 3.55  (3.02 to 4.08) |
| Japan | 1430  (1306 to 1621) | 6.65  (6.07 to 7.54) | 4322  (3308 to 5207) | 10.44  (7.99 to 12.58) | 2.02  (1.36 to 2.62) | 1.73  (1.51 to 1.95) |
| Jordan | 1  (1 to 2) | 0.86  (0.58 to 1.22) | 9  (5 to 12) | 1.26  (0.75 to 1.79) | 6.32  (3.34 to 11.34) | 1.47  (1.34 to 1.6) |
| Kazakhstan | 51  (40 to 71) | 3.38  (2.64 to 4.69) | 87  (66 to 117) | 4.26  (3.21 to 5.69) | 0.7  (0.29 to 1.28) | 1.8  (1.45 to 2.16) |
| Kenya | 10  (6 to 17) | 1.12  (0.68 to 1.84) | 36  (26 to 47) | 1.52  (1.08 to 1.99) | 2.44  (1.42 to 3.89) | 1.13  (1.09 to 1.16) |
| Kiribati | 0  (0 to 0) | 1.28  (0.68 to 1.94) | 0  (0 to 0) | 1.34  (0.65 to 2.1) | 0.88  (0.13 to 2.13) | -0.03  (-0.09 to 0.03) |
| Kuwait | 1  (1 to 2) | 2.24  (1.59 to 3.16) | 8  (6 to 11) | 3.42  (2.46 to 4.49) | 5.26  (3.17 to 8.43) | 2.31  (1.59 to 3.05) |
| Kyrgyzstan | 7  (6 to 10) | 1.93  (1.52 to 2.79) | 16  (12 to 19) | 3.09  (2.28 to 3.8) | 1.18  (0.31 to 1.99) | 2.45  (1.94 to 2.96) |
| Lao People's Democratic Republic | 5  (3 to 9) | 2.12  (1.29 to 3.63) | 14  (9 to 24) | 2.96  (1.84 to 4.97) | 1.81  (0.58 to 3.7) | 1.02  (0.97 to 1.07) |
| Latvia | 29  (20 to 36) | 6.27  (4.32 to 7.82) | 35  (20 to 46) | 6.85  (3.94 to 9) | 0.2  (-0.15 to 0.63) | 0.7  (0.42 to 0.98) |
| Lebanon | 19  (13 to 25) | 6.72  (4.57 to 9.1) | 45  (31 to 61) | 7.1  (4.9 to 9.76) | 1.4  (0.7 to 2.54) | 0.13  (0.07 to 0.19) |
| Lesotho | 1  (0 to 1) | 0.46  (0.26 to 0.76) | 1  (0 to 2) | 0.65  (0.33 to 1.07) | 0.76  (0.05 to 2.2) | 1.23  (1.17 to 1.29) |
| Liberia | 2  (1 to 3) | 1.34  (0.73 to 1.94) | 3  (1 to 5) | 1.62  (0.68 to 2.57) | 0.84  (0.04 to 2.36) | 1.25  (0.93 to 1.57) |
| Libya | 13  (8 to 17) | 6.03  (4.05 to 8.33) | 32  (20 to 46) | 6.17  (3.75 to 8.7) | 1.58  (0.54 to 3.14) | 0.15  (0.02 to 0.28) |
| Lithuania | 40  (32 to 63) | 7.03  (5.49 to 10.92) | 81  (44 to 106) | 11.22  (6.16 to 14.69) | 1  (-0.21 to 2.03) | 2.39  (2.09 to 2.7) |
| Luxembourg | 6  (5 to 8) | 8.74  (7.69 to 10.88) | 13  (10 to 17) | 11.03  (8.25 to 13.92) | 1.15  (0.46 to 1.85) | 1.02  (0.67 to 1.38) |
| Madagascar | 5  (3 to 8) | 0.83  (0.52 to 1.39) | 12  (7 to 17) | 1.03  (0.65 to 1.56) | 1.38  (0.49 to 3.23) | 0.83  (0.8 to 0.87) |
| Malawi | 7  (5 to 10) | 1.57  (1.08 to 2.21) | 15  (10 to 21) | 1.85  (1.18 to 2.61) | 1.15  (0.47 to 2.27) | 0.62  (0.52 to 0.72) |
| Malaysia | 65  (50 to 84) | 6.31  (4.88 to 8.17) | 262  (184 to 349) | 8.1  (5.7 to 10.81) | 3.06  (1.8 to 4.83) | 0.88  (0.66 to 1.09) |
| Maldives | 1  (0 to 1) | 5.57  (3.25 to 8.63) | 2  (1 to 2) | 6.22  (4.59 to 7.98) | 2.56  (1.21 to 5.3) | 0.34  (0.25 to 0.44) |
| Mali | 3  (2 to 5) | 0.67  (0.44 to 0.99) | 8  (5 to 12) | 0.87  (0.56 to 1.28) | 1.54  (0.51 to 3.21) | 0.82  (0.78 to 0.87) |
| Malta | 6  (5 to 7) | 11.61  (9.94 to 13.78) | 15  (12 to 19) | 12.83  (10.19 to 15.98) | 1.46  (0.9 to 2.2) | 0.22  (-0.01 to 0.45) |
| Marshall Islands | 0  (0 to 0) | 3.3  (1.86 to 5.47) | 0  (0 to 0) | 3.22  (1.88 to 5.13) | 0.97  (0.25 to 2.14) | -0.21  (-0.31 to -0.11) |
| Mauritania | 2  (1 to 3) | 1.94  (1.06 to 2.93) | 5  (2 to 7) | 2.01  (0.88 to 3.01) | 1.17  (0.32 to 2.54) | 0.66  (0.42 to 0.9) |
| Mauritius | 1  (1 to 1) | 1.04  (0.89 to 1.26) | 5  (3 to 6) | 2.05  (1.54 to 2.58) | 3.93  (2.61 to 5.48) | 2.29  (1.78 to 2.79) |
| Mexico | 135  (125 to 171) | 2.76  (2.55 to 3.5) | 600  (505 to 718) | 4.32  (3.63 to 5.17) | 3.44  (2.69 to 4.2) | 1.51  (1.38 to 1.63) |
| Micronesia (Federated States of) | 0  (0 to 0) | 3.15  (1.8 to 5.06) | 0  (0 to 0) | 3.36  (1.76 to 5.42) | 0.52  (-0.12 to 1.48) | 0.09  (-0.04 to 0.22) |
| Monaco | 1  (1 to 2) | 13.36  (8.58 to 21.01) | 2  (2 to 4) | 20.95  (13.06 to 31.27) | 1.01  (0.25 to 2.1) | 1.67  (1.34 to 2.01) |
| Mongolia | 5  (3 to 7) | 4.32  (2.68 to 5.69) | 12  (6 to 17) | 5.27  (2.69 to 7.32) | 1.4  (0.56 to 2.54) | 0.64  (0.45 to 0.83) |
| Montenegro | 5  (3 to 7) | 6.1  (4.44 to 9.07) | 9  (7 to 12) | 7.03  (5.18 to 9.07) | 0.99  (0.26 to 2.06) | 0.53  (0.43 to 0.62) |
| Morocco | 37  (25 to 51) | 2.33  (1.54 to 3.15) | 118  (73 to 164) | 3.2  (1.99 to 4.46) | 2.14  (1.1 to 3.65) | 0.96  (0.88 to 1.04) |
| Mozambique | 6  (4 to 10) | 0.86  (0.53 to 1.47) | 16  (10 to 24) | 1.31  (0.87 to 2) | 1.69  (0.66 to 3.53) | 1.62  (1.57 to 1.67) |
| Myanmar | 51  (28 to 87) | 1.9  (1.04 to 3.21) | 168  (112 to 270) | 3.12  (2.07 to 5.01) | 2.29  (0.97 to 4.65) | 1.89  (1.77 to 2.02) |
| Namibia | 0  (0 to 1) | 0.48  (0.3 to 0.75) | 1  (1 to 2) | 0.62  (0.36 to 0.97) | 1.32  (0.42 to 3.05) | 0.96  (0.93 to 1) |
| Nauru | 0  (0 to 0) | 4.15  (2.52 to 6.47) | 0  (0 to 0) | 3.53  (2.24 to 5.27) | -0.07  (-0.42 to 0.5) | -1  (-1.2 to -0.81) |
| Nepal | 23  (13 to 36) | 2.2  (1.29 to 3.47) | 85  (56 to 122) | 3.17  (2.07 to 4.53) | 2.73  (1.39 to 5.06) | 1.27  (1.22 to 1.32) |
| Netherlands | 215  (193 to 263) | 8.5  (7.64 to 10.39) | 599  (454 to 770) | 14.01  (10.62 to 18.01) | 1.79  (1.06 to 2.74) | 2.11  (1.89 to 2.32) |
| New Zealand | 76  (68 to 90) | 14.92  (13.32 to 17.66) | 157  (126 to 194) | 16.01  (12.81 to 19.82) | 1.08  (0.63 to 1.61) | 0.03  (-0.21 to 0.26) |
| Nicaragua | 3  (2 to 5) | 1.83  (1.43 to 2.65) | 17  (12 to 21) | 3.26  (2.41 to 4.17) | 4.31  (2.46 to 6.54) | 2.07  (1.94 to 2.2) |
| Niger | 4  (2 to 5) | 1.15  (0.62 to 1.77) | 13  (6 to 20) | 1.55  (0.7 to 2.36) | 2.78  (1.33 to 5.32) | 1.19  (1.04 to 1.33) |
| Nigeria | 35  (25 to 57) | 0.69  (0.49 to 1.12) | 82  (58 to 132) | 0.9  (0.64 to 1.45) | 1.34  (0.68 to 2.27) | 1.39  (1.15 to 1.63) |
| Niue | 0  (0 to 0) | 3.97  (2.57 to 5.61) | 0  (0 to 0) | 4.11  (2.88 to 5.52) | 0.04  (-0.28 to 0.57) | 0.22  (0.18 to 0.26) |
| North Macedonia | 8  (6 to 12) | 3.75  (2.45 to 5.48) | 14  (10 to 20) | 3.38  (2.34 to 4.67) | 0.7  (0.12 to 1.52) | -0.49  (-0.57 to -0.41) |
| Northern Mariana Islands | 0  (0 to 0) | 7.77  (4.32 to 10.91) | 0  (0 to 0) | 3.49  (2.21 to 6.26) | 0.89  (0.03 to 4) | -3.29  (-3.72 to -2.87) |
| Norway | 71  (64 to 82) | 8.17  (7.31 to 9.36) | 98  (80 to 115) | 8.35  (6.8 to 9.77) | 0.38  (0.14 to 0.63) | 0.18  (0.12 to 0.23) |
| Oman | 3  (2 to 5) | 4.4  (2.72 to 6.59) | 8  (6 to 11) | 5.14  (3.68 to 6.95) | 1.7  (0.75 to 3.39) | 0.66  (0.35 to 0.97) |
| Pakistan | 153  (99 to 273) | 2.36  (1.53 to 4.21) | 340  (241 to 488) | 2.86  (2.02 to 4.1) | 1.22  (0.53 to 2.41) | 0.62  (0.54 to 0.69) |
| Palau | 0  (0 to 0) | 1.88  (1.27 to 2.74) | 0  (0 to 0) | 2.02  (1.36 to 2.89) | 1.23  (0.42 to 2.61) | 0.19  (0.12 to 0.26) |
| Palestine | 1  (0 to 2) | 0.85  (0.48 to 1.47) | 3  (2 to 5) | 1.38  (0.93 to 2.06) | 3  (1.38 to 6.17) | 1.56  (1.4 to 1.72) |
| Panama | 7  (5 to 9) | 4.07  (3.07 to 4.97) | 27  (19 to 37) | 5.55  (3.86 to 7.43) | 2.87  (1.81 to 4.44) | 1.14  (1.06 to 1.21) |
| Papua New Guinea | 4  (2 to 7) | 1.89  (0.96 to 3.41) | 11  (6 to 19) | 2.26  (1.19 to 3.91) | 1.76  (0.84 to 3.35) | 0.4  (0.25 to 0.55) |
| Paraguay | 6  (4 to 9) | 2.25  (1.74 to 3.42) | 34  (22 to 46) | 5.22  (3.41 to 6.99) | 4.86  (2.29 to 7.94) | 3.7  (3.37 to 4.03) |
| Peru | 38  (28 to 53) | 2.78  (2.06 to 3.89) | 162  (109 to 223) | 4.29  (2.89 to 5.9) | 3.3  (1.58 to 5.88) | 1.89  (1.59 to 2.18) |
| Philippines | 148  (84 to 179) | 4.49  (2.55 to 5.45) | 357  (276 to 451) | 4.01  (3.1 to 5.07) | 1.42  (0.83 to 3.26) | -1.17  (-1.52 to -0.82) |
| Poland | 326  (267 to 612) | 5.76  (4.72 to 10.8) | 1005  (532 to 1269) | 10.77  (5.7 to 13.58) | 2.09  (-0.06 to 3.51) | 2.56  (2.23 to 2.88) |
| Portugal | 103  (88 to 143) | 5.58  (4.78 to 7.76) | 327  (237 to 422) | 11.17  (8.1 to 14.42) | 2.17  (0.92 to 3.38) | 2.27  (1.93 to 2.61) |
| Puerto Rico | 23  (14 to 27) | 4.94  (3.14 to 5.98) | 53  (32 to 72) | 5.84  (3.59 to 8.02) | 1.32  (0.68 to 2.15) | 0.49  (0.28 to 0.7) |
| Qatar | 1  (0 to 1) | 8.03  (4.65 to 12.12) | 6  (4 to 8) | 6.62  (4.12 to 9.85) | 7.19  (3.75 to 13.05) | -0.68  (-0.97 to -0.39) |
| Republic of Korea | 72  (48 to 134) | 2.16  (1.44 to 4.03) | 615  (398 to 796) | 5.41  (3.5 to 7) | 7.59  (2.07 to 13.08) | 4.28  (3.73 to 4.83) |
| Republic of Moldova | 21  (18 to 29) | 3.79  (3.17 to 5.11) | 27  (22 to 31) | 3.54  (2.86 to 4.17) | 0.26  (-0.12 to 0.64) | 0.33  (-0.14 to 0.81) |
| Romania | 111  (92 to 163) | 3.06  (2.54 to 4.5) | 328  (260 to 407) | 6.66  (5.28 to 8.25) | 1.97  (1.01 to 2.95) | 3.29  (3.11 to 3.47) |
| Russian Federation | 754  (603 to 889) | 3.23  (2.58 to 3.81) | 869  (741 to 1179) | 2.79  (2.38 to 3.78) | 0.15  (-0.09 to 0.63) | -1.03  (-1.35 to -0.7) |
| Rwanda | 3  (2 to 5) | 0.8  (0.53 to 1.38) | 7  (5 to 12) | 1.09  (0.68 to 1.7) | 1.78  (0.56 to 3.56) | 1.21  (1.08 to 1.34) |
| Saint Kitts and Nevis | 0  (0 to 0) | 0.63  (0.51 to 0.83) | 0  (0 to 0) | 0.88  (0.69 to 1.12) | 1.17  (0.63 to 1.85) | 0.86  (0.6 to 1.11) |
| Saint Lucia | 0  (0 to 0) | 2.01  (1.63 to 2.77) | 1  (1 to 1) | 2.71  (2.15 to 3.34) | 2.09  (1.29 to 3.13) | 0.89  (0.73 to 1.05) |
| Saint Vincent and the Grenadines | 0  (0 to 0) | 0.98  (0.77 to 1.59) | 0  (0 to 0) | 2.31  (1.88 to 2.77) | 3.32  (1.87 to 4.88) | 2.8  (2.49 to 3.12) |
| Samoa | 0  (0 to 1) | 3.46  (2.32 to 4.89) | 1  (0 to 1) | 3.52  (2.45 to 5.1) | 0.61  (0.07 to 1.4) | 0.09  (0.04 to 0.13) |
| San Marino | 0  (0 to 1) | 9.85  (7.54 to 12.56) | 1  (1 to 1) | 12.9  (8.8 to 18.67) | 1.29  (0.55 to 2.55) | 1.22  (1.08 to 1.36) |
| Sao Tome and Principe | 0  (0 to 0) | 1.28  (0.68 to 1.86) | 0  (0 to 0) | 1.63  (0.74 to 2.39) | 0.78  (0.15 to 1.85) | 0.99  (0.92 to 1.05) |
| Saudi Arabia | 22  (9 to 33) | 3.64  (1.5 to 5.48) | 60  (32 to 85) | 3.74  (1.97 to 5.26) | 1.72  (0.61 to 3.72) | -0.36  (-0.6 to -0.11) |
| Senegal | 5  (3 to 8) | 1.37  (0.78 to 2.05) | 17  (8 to 25) | 2.02  (0.91 to 2.97) | 2.37  (1.06 to 4.45) | 1.73  (1.51 to 1.96) |
| Serbia | 44  (30 to 67) | 2.96  (2.02 to 4.55) | 101  (74 to 141) | 4.62  (3.39 to 6.42) | 1.32  (0.54 to 2.62) | 2.14  (1.82 to 2.46) |
| Seychelles | 0  (0 to 0) | 2.66  (1.43 to 3.88) | 0  (0 to 1) | 3.14  (1.56 to 4.63) | 1.14  (0.41 to 2.29) | 0.38  (0.27 to 0.48) |
| Sierra Leone | 2  (1 to 4) | 1.09  (0.61 to 1.63) | 6  (3 to 9) | 1.61  (0.67 to 2.41) | 1.56  (0.51 to 3.31) | 1.74  (1.58 to 1.89) |
| Singapore | 23  (20 to 26) | 8.91  (7.9 to 10.39) | 89  (70 to 110) | 8.99  (7.07 to 11.21) | 2.93  (2.01 to 4.06) | -0.02  (-0.33 to 0.29) |
| Slovakia | 79  (53 to 97) | 10.11  (6.77 to 12.49) | 128  (70 to 174) | 10.43  (5.7 to 14.19) | 0.62  (0.14 to 1.22) | 0.22  (0.03 to 0.4) |
| Slovenia | 17  (13 to 23) | 5.64  (4.27 to 7.34) | 73  (40 to 99) | 13.43  (7.34 to 18.15) | 3.19  (1.21 to 5.32) | 3.9  (3.41 to 4.4) |
| Solomon Islands | 0  (0 to 1) | 2.19  (1.11 to 4.17) | 1  (0 to 1) | 2.54  (1.43 to 4.47) | 1.41  (0.53 to 3.25) | 0.32  (0.19 to 0.46) |
| Somalia | 2  (1 to 3) | 0.68  (0.36 to 1.24) | 6  (2 to 11) | 0.83  (0.33 to 1.55) | 2.59  (0.8 to 5.41) | 1  (0.89 to 1.12) |
| South Africa | 8  (6 to 16) | 0.35  (0.23 to 0.67) | 25  (18 to 33) | 0.47  (0.35 to 0.63) | 1.95  (0.86 to 3.51) | 1.2  (1.07 to 1.34) |
| South Sudan | 4  (2 to 8) | 1.31  (0.6 to 2.87) | 6  (3 to 12) | 1.63  (0.83 to 2.95) | 0.82  (0.09 to 2.15) | 0.9  (0.83 to 0.97) |
| Spain | 317  (266 to 499) | 4.42  (3.7 to 6.95) | 1184  (863 to 1531) | 10.55  (7.69 to 13.63) | 2.73  (1.01 to 4.42) | 3.39  (3.1 to 3.67) |
| Sri Lanka | 37  (27 to 51) | 2.96  (2.18 to 4.13) | 134  (90 to 197) | 4.07  (2.75 to 5.98) | 2.65  (1.32 to 4.68) | 1.13  (1.03 to 1.23) |
| Sudan | 43  (21 to 75) | 4.01  (1.93 to 6.99) | 109  (57 to 176) | 5.49  (2.88 to 8.87) | 1.55  (0.69 to 3.15) | 1.13  (1.08 to 1.17) |
| Suriname | 0  (0 to 1) | 1.51  (1.1 to 2.19) | 2  (1 to 2) | 2.13  (1.55 to 2.89) | 2.32  (1.3 to 3.9) | 1.32  (1.21 to 1.44) |
| Sweden | 186  (167 to 222) | 9.72  (8.72 to 11.61) | 334  (271 to 400) | 13.2  (10.71 to 15.8) | 0.8  (0.45 to 1.18) | 0.88  (0.64 to 1.12) |
| Switzerland | 132  (110 to 156) | 10.21  (8.55 to 12.04) | 226  (162 to 301) | 10.97  (7.84 to 14.6) | 0.72  (0.18 to 1.33) | 0.77  (0.52 to 1.01) |
| Syrian Arab Republic | 53  (33 to 82) | 9.11  (5.54 to 14.01) | 134  (78 to 214) | 9.29  (5.41 to 14.84) | 1.5  (0.53 to 3.16) | -0.27  (-0.55 to 0.01) |
| Taiwan (Province of China) | 31  (25 to 45) | 1.57  (1.28 to 2.26) | 343  (135 to 485) | 6.71  (2.63 to 9.5) | 9.92  (2.49 to 16.2) | 8.14  (6.98 to 9.32) |
| Tajikistan | 6  (4 to 10) | 1.95  (1.32 to 2.97) | 16  (11 to 22) | 3.03  (2.08 to 4.15) | 1.47  (0.49 to 3.19) | 2.1  (1.76 to 2.43) |
| Thailand | 38  (27 to 54) | 0.95  (0.68 to 1.36) | 356  (136 to 533) | 2.8  (1.07 to 4.19) | 8.45  (2.86 to 15.49) | 4.83  (4.29 to 5.37) |
| Timor-Leste | 1  (0 to 1) | 1.93  (1.06 to 3.63) | 3  (2 to 5) | 2.91  (1.71 to 5.06) | 4.79  (2.63 to 8.44) | 1.71  (1.54 to 1.87) |
| Togo | 2  (1 to 2) | 1.28  (0.7 to 1.85) | 7  (3 to 11) | 1.91  (0.81 to 2.98) | 3.3  (1.52 to 6.45) | 1.54  (1.44 to 1.63) |
| Tokelau | 0  (0 to 0) | 3.05  (1.72 to 4.69) | 0  (0 to 0) | 3.15  (2.06 to 4.36) | -0.09  (-0.4 to 0.41) | -0.08  (-0.32 to 0.16) |
| Tonga | 0  (0 to 0) | 2.33  (1.47 to 3.55) | 0  (0 to 0) | 2.96  (2.03 to 4.11) | 0.78  (0.2 to 1.75) | 0.66  (0.44 to 0.88) |
| Trinidad and Tobago | 4  (3 to 4) | 3.72  (2.97 to 4.35) | 9  (7 to 12) | 3.97  (2.98 to 5.21) | 1.47  (0.82 to 2.33) | 0.25  (0.15 to 0.34) |
| Tunisia | 16  (11 to 23) | 2.58  (1.83 to 3.81) | 49  (33 to 76) | 3.17  (2.12 to 4.9) | 2.13  (0.96 to 4.03) | 0.7  (0.59 to 0.81) |
| Turkey | 350  (262 to 451) | 8.48  (6.36 to 10.93) | 1017  (769 to 1288) | 9.53  (7.21 to 12.07) | 1.91  (1.02 to 3.22) | 0.49  (0.43 to 0.55) |
| Turkmenistan | 4  (3 to 5) | 1.58  (1.18 to 2.3) | 11  (7 to 15) | 2.49  (1.74 to 3.56) | 2.04  (1.15 to 3.56) | 1.89  (1.68 to 2.1) |
| Tuvalu | 0  (0 to 0) | 2.9  (1.53 to 5.04) | 0  (0 to 0) | 3.17  (1.93 to 4.84) | 0.62  (0.04 to 1.54) | 0.09  (-0.07 to 0.25) |
| Uganda | 5  (3 to 7) | 0.69  (0.45 to 0.99) | 13  (9 to 18) | 0.87  (0.59 to 1.23) | 1.54  (0.56 to 2.97) | 0.9  (0.82 to 0.98) |
| Ukraine | 460  (368 to 596) | 4.84  (3.88 to 6.28) | 448  (351 to 569) | 4.46  (3.49 to 5.66) | -0.03  (-0.28 to 0.3) | -0.61  (-0.72 to -0.49) |
| United Arab Emirates | 3  (1 to 5) | 10.17  (4.59 to 16.69) | 25  (11 to 42) | 9.5  (4.18 to 15.75) | 7  (3.55 to 14.06) | -0.56  (-0.95 to -0.17) |
| United Kingdom | 1443  (1295 to 1589) | 12.3  (11.03 to 13.54) | 2340  (1858 to 2792) | 15.34  (12.18 to 18.31) | 0.62  (0.31 to 0.93) | 0.68  (0.54 to 0.81) |
| United Republic of Tanzania | 12  (7 to 21) | 0.96  (0.57 to 1.68) | 37  (24 to 57) | 1.4  (0.91 to 2.15) | 2.09  (0.99 to 4.23) | 1.57  (1.45 to 1.69) |
| United States of America | 5327  (4886 to 6123) | 13  (11.93 to 14.95) | 12147  (9810 to 14138) | 17.01  (13.74 to 19.8) | 1.28  (0.86 to 1.68) | 0.89  (0.69 to 1.08) |
| United States Virgin Islands | 0  (0 to 1) | 3.95  (2.75 to 5.23) | 1  (1 to 2) | 5.04  (3.72 to 6.42) | 2.31  (1.31 to 3.67) | 0.98  (0.87 to 1.09) |
| Uruguay | 31  (25 to 37) | 6.16  (4.93 to 7.27) | 48  (36 to 63) | 7.22  (5.33 to 9.48) | 0.54  (0.12 to 1.06) | 0.31  (0.13 to 0.5) |
| Uzbekistan | 38  (29 to 50) | 2.87  (2.17 to 3.75) | 78  (58 to 105) | 3.51  (2.62 to 4.72) | 1.05  (0.43 to 1.99) | 0.86  (0.68 to 1.05) |
| Vanuatu | 0  (0 to 0) | 2.51  (1.41 to 4.41) | 1  (0 to 1) | 2.92  (1.72 to 4.68) | 2.2  (1.06 to 4.07) | 0.29  (0.15 to 0.43) |
| Venezuela (Bolivarian Republic of) | 40  (35 to 49) | 3.64  (3.16 to 4.44) | 194  (147 to 255) | 5.5  (4.15 to 7.21) | 3.84  (2.52 to 5.67) | 1.46  (1.11 to 1.81) |
| Viet Nam | 214  (146 to 281) | 4.36  (2.98 to 5.72) | 618  (403 to 858) | 5.78  (3.77 to 8.03) | 1.89  (0.85 to 3.39) | 1.2  (1.02 to 1.37) |
| Yemen | 18  (10 to 29) | 3.33  (1.8 to 5.29) | 63  (29 to 101) | 4.36  (2.02 to 6.98) | 2.47  (1.12 to 4.46) | 1.18  (1.04 to 1.32) |
| Zambia | 4  (2 to 6) | 1.15  (0.72 to 1.97) | 10  (7 to 16) | 1.52  (1.02 to 2.28) | 1.94  (0.86 to 4.02) | 1.09  (1.01 to 1.17) |
| Zimbabwe | 4  (2 to 6) | 0.83  (0.52 to 1.35) | 6  (4 to 11) | 0.78  (0.47 to 1.35) | 0.57  (-0.08 to 1.6) | -0.42  (-0.59 to -0.24) |

| location | Death cases in 1990 | Death rates in 1990 | Death cases in 2019 | Death rate in  2019 | Cases change | EAPC |
| --- | --- | --- | --- | --- | --- | --- |
| Afghanistan | 45  (23 to 78) | 5.17  (2.71 to 9.01) | 60  (30 to 100) | 5.59  (2.74 to 9.28) | 0.35  (-0.17 to 1.14) | 0.35  (0.24 to 0.46) |
| Albania | 13  (10 to 21) | 5.51  (4.07 to 8.6) | 31  (20 to 52) | 5.53  (3.48 to 9.25) | 1.38  (0.61 to 2.56) | 0.12  (0.01 to 0.22) |
| Algeria | 48  (32 to 67) | 3.3  (2.21 to 4.56) | 141  (92 to 194) | 3.58  (2.34 to 4.93) | 1.91  (0.94 to 3.58) | 0.35  (0.26 to 0.44) |
| American Samoa | 0  (0 to 0) | 2.93  (2.1 to 3.79) | 0  (0 to 0) | 2.29  (1.69 to 3.09) | 0.75  (0.16 to 1.74) | -1.05  (-1.26 to -0.84) |
| Andorra | 1  (1 to 2) | 17.21  (11.68 to 24.14) | 3  (2 to 4) | 18.23  (12.18 to 24.29) | 1.48  (0.61 to 2.71) | 0.21  (0.1 to 0.32) |
| Angola | 5  (3 to 8) | 1.15  (0.69 to 1.86) | 17  (10 to 26) | 1.47  (0.88 to 2.24) | 2.62  (1.16 to 5.12) | 0.98  (0.89 to 1.07) |
| Antigua and Barbuda | 0  (0 to 0) | 2.44  (1.91 to 3.13) | 0  (0 to 1) | 3.96  (3.01 to 5.12) | 1.96  (1.12 to 3.21) | 1.92  (1.73 to 2.11) |
| Argentina | 194  (160 to 233) | 4.72  (3.91 to 5.66) | 463  (347 to 602) | 6.85  (5.13 to 8.9) | 1.39  (0.76 to 2.24) | 1.12  (0.83 to 1.41) |
| Armenia | 6  (4 to 8) | 1.79  (1.32 to 2.43) | 18  (11 to 24) | 3.25  (1.94 to 4.37) | 2.04  (0.77 to 3.59) | 2  (1.72 to 2.28) |
| Australia | 346  (295 to 378) | 13.65  (11.64 to 14.93) | 829  (591 to 1085) | 15.96  (11.37 to 20.9) | 1.4  (0.81 to 2.12) | 0.34  (0.23 to 0.45) |
| Austria | 111  (99 to 138) | 7.28  (6.46 to 8.99) | 311  (202 to 400) | 14.63  (9.49 to 18.79) | 1.79  (0.68 to 2.67) | 2.94  (2.64 to 3.25) |
| Azerbaijan | 11  (7 to 16) | 1.88  (1.29 to 2.87) | 29  (19 to 45) | 2.74  (1.83 to 4.19) | 1.76  (0.87 to 3.05) | 1.61  (1.45 to 1.76) |
| Bahamas | 1  (0 to 1) | 3.21  (2.74 to 3.99) | 2  (1 to 2) | 3.71  (2.95 to 4.66) | 2.04  (1.32 to 2.95) | 0.76  (0.66 to 0.86) |
| Bahrain | 2  (1 to 2) | 9.41  (5.72 to 13.77) | 8  (5 to 11) | 7.49  (4.97 to 10.34) | 3.64  (1.95 to 6.62) | -1.62  (-2.03 to -1.21) |
| Bangladesh | 147  (93 to 214) | 2.81  (1.78 to 4.1) | 472  (286 to 743) | 3.02  (1.83 to 4.75) | 2.22  (1.01 to 4.03) | 0.1  (0 to 0.21) |
| Barbados | 2  (2 to 2) | 4.96  (4.25 to 6.11) | 4  (4 to 5) | 7.04  (5.68 to 8.48) | 1.41  (0.88 to 2.04) | 1.42  (1.17 to 1.67) |
| Belarus | 145  (108 to 173) | 8.65  (6.43 to 10.31) | 177  (122 to 236) | 8.53  (5.88 to 11.38) | 0.22  (-0.11 to 0.67) | -0.07  (-0.22 to 0.08) |
| Belgium | 172  (147 to 225) | 8.58  (7.35 to 11.27) | 395  (286 to 510) | 14.29  (10.35 to 18.47) | 1.3  (0.48 to 2.15) | 1.67  (1.42 to 1.93) |
| Belize | 0  (0 to 0) | 0.45  (0.33 to 0.64) | 0  (0 to 0) | 0.7  (0.53 to 0.92) | 3.25  (1.95 to 5.33) | 1.47  (1.27 to 1.67) |
| Benin | 3  (2 to 4) | 1.3  (0.75 to 1.86) | 10  (5 to 14) | 1.86  (0.89 to 2.73) | 2.22  (0.98 to 4.26) | 1.39  (1.26 to 1.53) |
| Bermuda | 0  (0 to 1) | 5.61  (4.4 to 7.08) | 1  (1 to 1) | 5.31  (4.14 to 6.68) | 1.03  (0.5 to 1.76) | -0.19  (-0.27 to -0.11) |
| Bhutan | 1  (0 to 1) | 2.58  (1.51 to 4.1) | 3  (2 to 5) | 4.04  (2.43 to 6.84) | 2.91  (1.43 to 5.42) | 1.63  (1.49 to 1.77) |
| Bolivia (Plurinational State of) | 12  (8 to 18) | 3.35  (2.2 to 4.97) | 62  (41 to 88) | 5.85  (3.83 to 8.28) | 4.03  (2.26 to 6.79) | 1.99  (1.86 to 2.13) |
| Bosnia and Herzegovina | 20  (13 to 35) | 4.25  (2.82 to 7.34) | 55  (38 to 73) | 6.89  (4.72 to 9.13) | 1.7  (0.38 to 3.56) | 2.03  (1.77 to 2.29) |
| Botswana | 0  (0 to 1) | 0.66  (0.37 to 1.11) | 1  (1 to 2) | 0.87  (0.42 to 1.51) | 1.99  (0.64 to 4.21) | 0.62  (0.39 to 0.86) |
| Brazil | 466  (417 to 533) | 4.48  (4.02 to 5.13) | 1809  (1452 to 1982) | 6.28  (5.04 to 6.88) | 2.88  (2.22 to 3.25) | 1.33  (1.18 to 1.48) |
| Brunei Darussalam | 0  (0 to 1) | 3.51  (2.15 to 5.08) | 1  (1 to 1) | 3.26  (2.42 to 4.43) | 2.07  (0.95 to 4.28) | -0.35  (-0.62 to -0.07) |
| Bulgaria | 80  (63 to 97) | 4.79  (3.74 to 5.79) | 141  (107 to 182) | 7.29  (5.56 to 9.43) | 0.75  (0.28 to 1.39) | 2.12  (1.83 to 2.42) |
| Burkina Faso | 6  (3 to 10) | 1.2  (0.65 to 1.86) | 17  (8 to 27) | 1.76  (0.79 to 2.69) | 1.83  (0.74 to 3.72) | 1.42  (1.23 to 1.62) |
| Burundi | 2  (1 to 3) | 0.76  (0.47 to 1.28) | 6  (3 to 9) | 1.13  (0.62 to 1.88) | 1.71  (0.32 to 3.79) | 1.55  (1.46 to 1.64) |
| Cabo Verde | 0  (0 to 1) | 1.23  (0.65 to 1.78) | 1  (1 to 2) | 2.59  (1.04 to 3.89) | 2.52  (1.07 to 4.64) | 2.66  (2.09 to 3.23) |
| Cambodia | 10  (6 to 17) | 2.04  (1.2 to 3.43) | 42  (27 to 68) | 3.03  (1.94 to 4.97) | 3.07  (1.63 to 5.32) | 1.44  (1.39 to 1.49) |
| Cameroon | 9  (5 to 13) | 1.77  (1.02 to 2.7) | 30  (13 to 46) | 2.37  (1.04 to 3.61) | 2.43  (0.98 to 4.93) | 1.26  (1.15 to 1.37) |
| Canada | 349  (309 to 456) | 8.41  (7.45 to 10.99) | 1073  (812 to 1400) | 12.28  (9.28 to 16.02) | 2.08  (1.18 to 3.17) | 1.78  (1.55 to 2) |
| Central African Republic | 1  (1 to 2) | 1.11  (0.66 to 1.79) | 2  (1 to 4) | 1.1  (0.58 to 1.95) | 0.7  (-0.06 to 2.08) | 0.05  (-0.01 to 0.11) |
| Chad | 4  (2 to 6) | 1.15  (0.63 to 1.75) | 10  (5 to 15) | 1.69  (0.79 to 2.48) | 1.7  (0.7 to 3.39) | 1.65  (1.52 to 1.79) |
| Chile | 46  (39 to 62) | 3.85  (3.21 to 5.16) | 192  (145 to 250) | 6.42  (4.85 to 8.35) | 3.15  (1.9 to 4.7) | 2.09  (1.87 to 2.31) |
| China | 980  (725 to 1362) | 0.97  (0.72 to 1.35) | 3975  (3180 to 4950) | 1.56  (1.25 to 1.95) | 3.05  (1.8 to 4.95) | 1.9  (1.75 to 2.05) |
| Colombia | 56  (47 to 77) | 2.79  (2.38 to 3.85) | 303  (218 to 399) | 4.74  (3.42 to 6.25) | 4.46  (2.53 to 6.75) | 1.95  (1.75 to 2.14) |
| Comoros | 0  (0 to 0) | 1.03  (0.55 to 1.92) | 1  (0 to 1) | 1.34  (0.84 to 2.08) | 1.78  (0.55 to 4.02) | 0.95  (0.89 to 1) |
| Congo | 2  (1 to 3) | 1.52  (0.96 to 2.44) | 5  (3 to 7) | 1.72  (1.07 to 2.49) | 1.48  (0.49 to 3.4) | 0.36  (0.25 to 0.48) |
| Cook Islands | 0  (0 to 0) | 2.99  (2.19 to 3.93) | 0  (0 to 0) | 3.11  (2.33 to 4.07) | 1.18  (0.51 to 2.21) | -0.01  (-0.07 to 0.05) |
| Costa Rica | 12  (10 to 15) | 6.01  (4.79 to 7.26) | 48  (35 to 65) | 7.74  (5.68 to 10.36) | 2.93  (1.8 to 4.45) | 0.99  (0.85 to 1.12) |
| C么te d'Ivoire | 6  (3 to 9) | 1.4  (0.81 to 2.16) | 20  (10 to 31) | 1.85  (0.88 to 2.82) | 2.48  (1.14 to 4.95) | 1.06  (1 to 1.12) |
| Croatia | 22  (14 to 53) | 2.67  (1.75 to 6.45) | 119  (61 to 163) | 10.44  (5.33 to 14.22) | 4.45  (0.35 to 8.97) | 5.28  (4.65 to 5.91) |
| Cuba | 54  (46 to 71) | 4.26  (3.6 to 5.58) | 120  (93 to 156) | 5.3  (4.13 to 6.88) | 1.22  (0.72 to 1.81) | 1.11  (0.95 to 1.28) |
| Cyprus | 9  (7 to 12) | 8.47  (6.4 to 10.84) | 34  (16 to 46) | 13.58  (6.28 to 18.28) | 2.77  (0.88 to 4.58) | 2.24  (1.94 to 2.54) |
| Czechia | 166  (146 to 205) | 9.15  (8.05 to 11.3) | 299  (222 to 378) | 10.96  (8.15 to 13.88) | 0.8  (0.23 to 1.34) | 1.2  (0.96 to 1.43) |
| Democratic People's Republic of Korea | 24  (15 to 37) | 1.35  (0.84 to 2.11) | 59  (34 to 88) | 1.55  (0.89 to 2.3) | 1.48  (0.54 to 3.01) | 0.67  (0.58 to 0.76) |
| Democratic Republic of the Congo | 20  (12 to 31) | 1.11  (0.68 to 1.73) | 45  (23 to 78) | 1.2  (0.61 to 2.1) | 1.27  (0.16 to 2.94) | 0.22  (-0.01 to 0.45) |
| Denmark | 180  (151 to 198) | 17.6  (14.72 to 19.28) | 204  (153 to 261) | 14.27  (10.69 to 18.2) | 0.13  (-0.12 to 0.43) | -0.44  (-0.67 to -0.2) |
| Djibouti | 0  (0 to 0) | 1.04  (0.53 to 2.02) | 1  (1 to 2) | 1.62  (0.95 to 2.74) | 5.93  (3.23 to 11.46) | 1.65  (1.57 to 1.72) |
| Dominica | 0  (0 to 0) | 3.06  (2.16 to 4.33) | 0  (0 to 1) | 4.07  (2.79 to 5.57) | 0.58  (0.06 to 1.44) | 1.19  (1.06 to 1.33) |
| Dominican Republic | 4  (3 to 8) | 1.02  (0.69 to 1.82) | 28  (18 to 40) | 2.56  (1.62 to 3.68) | 5.37  (2.14 to 10.48) | 3.97  (3.73 to 4.21) |
| Ecuador | 19  (14 to 28) | 3.02  (2.27 to 4.57) | 120  (75 to 160) | 6.59  (4.11 to 8.79) | 5.46  (2.07 to 9.02) | 3.58  (3.22 to 3.94) |
| Egypt | 77  (47 to 130) | 2.34  (1.44 to 3.96) | 217  (122 to 374) | 2.99  (1.68 to 5.16) | 1.83  (0.72 to 3.37) | 0.79  (0.62 to 0.96) |
| El Salvador | 2  (2 to 4) | 0.67  (0.5 to 1.09) | 17  (8 to 24) | 2.39  (1.06 to 3.43) | 6.35  (1.72 to 11.45) | 5.47  (4.82 to 6.12) |
| Equatorial Guinea | 0  (0 to 0) | 1.01  (0.56 to 1.69) | 1  (1 to 2) | 1.98  (1.01 to 3.51) | 3.55  (1.1 to 8.92) | 3.05  (2.77 to 3.32) |
| Eritrea | 1  (0 to 1) | 0.69  (0.39 to 1.14) | 3  (2 to 5) | 1.19  (0.76 to 1.76) | 3.88  (1.89 to 7.63) | 1.76  (1.5 to 2.01) |
| Estonia | 16  (11 to 33) | 6.04  (4.19 to 12.43) | 38  (29 to 50) | 11.46  (8.67 to 14.85) | 1.41  (0.15 to 2.84) | 3.1  (2.45 to 3.76) |
| Eswatini | 0  (0 to 0) | 0.69  (0.4 to 1.11) | 1  (0 to 1) | 0.83  (0.38 to 1.33) | 1.52  (0.46 to 3.44) | 0.62  (0.36 to 0.87) |
| Ethiopia | 102  (44 to 181) | 4.65  (2 to 8.29) | 249  (109 to 384) | 5.53  (2.43 to 8.53) | 1.44  (0.58 to 2.95) | 0.63  (0.58 to 0.68) |
| Fiji | 3  (2 to 4) | 9.12  (6.73 to 12.04) | 9  (7 to 12) | 10.41  (7.67 to 13.68) | 1.68  (0.81 to 3) | 0.59  (0.47 to 0.71) |
| Finland | 109  (96 to 123) | 11.89  (10.5 to 13.36) | 177  (134 to 229) | 11.51  (8.72 to 14.88) | 0.62  (0.23 to 1.14) | -0.13  (-0.19 to -0.06) |
| France | 790  (707 to 974) | 7.44  (6.65 to 9.18) | 2080  (1410 to 2729) | 12.65  (8.58 to 16.6) | 1.63  (0.66 to 2.59) | 2.22  (2 to 2.45) |
| Gabon | 1  (1 to 2) | 1.84  (0.96 to 3.52) | 2  (1 to 4) | 2.15  (1.19 to 3.4) | 1  (0.09 to 2.89) | 0.44  (0.38 to 0.5) |
| Gambia | 0  (0 to 0) | 0.56  (0.33 to 0.86) | 1  (1 to 1) | 0.77  (0.5 to 1.14) | 2.8  (1.16 to 6.05) | 1.03  (0.82 to 1.25) |
| Georgia | 17  (12 to 30) | 2.19  (1.53 to 3.78) | 30  (17 to 40) | 3.84  (2.26 to 5.16) | 0.7  (-0.35 to 1.78) | 2.99  (1.72 to 4.28) |
| Germany | 2074  (1759 to 2412) | 12.99  (11.01 to 15.1) | 5818  (3745 to 7703) | 25.29  (16.28 to 33.49) | 1.8  (0.87 to 2.75) | 3  (2.78 to 3.23) |
| Ghana | 14  (9 to 21) | 2.06  (1.33 to 3.06) | 45  (32 to 62) | 2.53  (1.82 to 3.51) | 2.17  (1.01 to 4.3) | 0.58  (0.5 to 0.67) |
| Greece | 157  (138 to 178) | 7.93  (6.98 to 8.99) | 460  (284 to 605) | 16.32  (10.09 to 21.45) | 1.93  (0.82 to 2.85) | 2.66  (2.51 to 2.81) |
| Greenland | 0  (0 to 0) | 4.72  (3.57 to 6.03) | 0  (0 to 1) | 4.73  (3.43 to 6.81) | 1.33  (0.61 to 2.19) | 0.32  (0.16 to 0.48) |
| Grenada | 0  (0 to 0) | 1.6  (1.2 to 2.05) | 0  (0 to 0) | 2.37  (1.84 to 3.27) | 1.13  (0.51 to 2.06) | 1.61  (1.44 to 1.77) |
| Guam | 0  (0 to 1) | 5.6  (3.83 to 7.21) | 1  (0 to 1) | 2.55  (1.55 to 5.07) | 0.2  (-0.33 to 2.11) | -3.07  (-3.36 to -2.78) |
| Guatemala | 2  (2 to 4) | 0.53  (0.37 to 0.88) | 28  (20 to 36) | 2.15  (1.49 to 2.78) | 11.7  (5.39 to 19.87) | 5.98  (5.52 to 6.43) |
| Guinea | 3  (2 to 5) | 0.83  (0.54 to 1.25) | 6  (4 to 9) | 1  (0.65 to 1.53) | 0.86  (0.12 to 2.19) | 0.47  (0.37 to 0.57) |
| Guinea-Bissau | 1  (0 to 1) | 1.43  (0.81 to 2.15) | 2  (1 to 2) | 2.04  (0.88 to 3.14) | 1.36  (0.36 to 2.92) | 1.42  (1.33 to 1.52) |
| Guyana | 0  (0 to 0) | 0.83  (0.57 to 1.1) | 1  (0 to 1) | 0.83  (0.55 to 1.22) | 0.68  (0.01 to 1.95) | -0.06  (-0.24 to 0.11) |
| Haiti | 8  (4 to 13) | 2.02  (1.06 to 3.52) | 19  (9 to 33) | 2.47  (1.19 to 4.28) | 1.55  (0.6 to 3.02) | 0.81  (0.75 to 0.87) |
| Honduras | 8  (5 to 11) | 3.22  (2.21 to 4.5) | 38  (26 to 54) | 5.44  (3.65 to 7.58) | 4.11  (2.22 to 6.94) | 1.98  (1.88 to 2.09) |
| Hungary | 158  (140 to 179) | 8.1  (7.21 to 9.21) | 309  (230 to 386) | 12.13  (9 to 15.12) | 0.96  (0.45 to 1.44) | 1.46  (1.06 to 1.85) |
| Iceland | 3  (3 to 4) | 9.22  (7.76 to 10.67) | 7  (6 to 9) | 10.43  (8.16 to 13.68) | 1.16  (0.65 to 1.91) | 0.43  (0.3 to 0.57) |
| India | 1234  (943 to 1570) | 2.48  (1.89 to 3.15) | 4462  (3675 to 5462) | 3.26  (2.69 to 3.99) | 2.62  (1.73 to 3.85) | 0.82  (0.72 to 0.92) |
| Indonesia | 224  (171 to 378) | 2.08  (1.59 to 3.51) | 804  (551 to 1327) | 3.29  (2.25 to 5.43) | 2.59  (1.68 to 3.84) | 1.53  (1.44 to 1.63) |
| Iran (Islamic Republic of) | 154  (120 to 206) | 5.04  (3.93 to 6.74) | 471  (382 to 672) | 5.6  (4.55 to 8) | 2.05  (1.37 to 2.96) | 0.46  (0.27 to 0.65) |
| Iraq | 26  (16 to 45) | 2.98  (1.88 to 5.1) | 88  (57 to 141) | 3.53  (2.28 to 5.68) | 2.37  (1.2 to 4.4) | 0.55  (0.41 to 0.69) |
| Ireland | 50  (43 to 62) | 9.43  (8.18 to 11.7) | 117  (85 to 150) | 12.51  (9.12 to 16.13) | 1.33  (0.61 to 2.16) | 1.08  (0.94 to 1.21) |
| Israel | 81  (65 to 93) | 12.81  (10.37 to 14.77) | 242  (164 to 318) | 16.73  (11.32 to 21.92) | 2.01  (1.21 to 3.05) | 0.82  (0.58 to 1.06) |
| Italy | 774  (684 to 1172) | 6.65  (5.88 to 10.07) | 2294  (1687 to 2766) | 13.62  (10.02 to 16.42) | 1.96  (0.72 to 2.85) | 2.87  (2.52 to 3.23) |
| Jamaica | 3  (2 to 4) | 1.24  (0.95 to 1.7) | 12  (8 to 15) | 3.25  (2.34 to 4.29) | 3.13  (1.61 to 5.06) | 3.55  (3.02 to 4.08) |
| Japan | 1430  (1306 to 1621) | 6.65  (6.07 to 7.54) | 4322  (3308 to 5207) | 10.44  (7.99 to 12.58) | 2.02  (1.36 to 2.62) | 1.73  (1.51 to 1.95) |
| Jordan | 1  (1 to 2) | 0.86  (0.58 to 1.22) | 9  (5 to 12) | 1.26  (0.75 to 1.79) | 6.32  (3.34 to 11.34) | 1.47  (1.34 to 1.6) |
| Kazakhstan | 51  (40 to 71) | 3.38  (2.64 to 4.69) | 87  (66 to 117) | 4.26  (3.21 to 5.69) | 0.7  (0.29 to 1.28) | 1.8  (1.45 to 2.16) |
| Kenya | 10  (6 to 17) | 1.12  (0.68 to 1.84) | 36  (26 to 47) | 1.52  (1.08 to 1.99) | 2.44  (1.42 to 3.89) | 1.13  (1.09 to 1.16) |
| Kiribati | 0  (0 to 0) | 1.28  (0.68 to 1.94) | 0  (0 to 0) | 1.34  (0.65 to 2.1) | 0.88  (0.13 to 2.13) | -0.03  (-0.09 to 0.03) |
| Kuwait | 1  (1 to 2) | 2.24  (1.59 to 3.16) | 8  (6 to 11) | 3.42  (2.46 to 4.49) | 5.26  (3.17 to 8.43) | 2.31  (1.59 to 3.05) |
| Kyrgyzstan | 7  (6 to 10) | 1.93  (1.52 to 2.79) | 16  (12 to 19) | 3.09  (2.28 to 3.8) | 1.18  (0.31 to 1.99) | 2.45  (1.94 to 2.96) |
| Lao People's Democratic Republic | 5  (3 to 9) | 2.12  (1.29 to 3.63) | 14  (9 to 24) | 2.96  (1.84 to 4.97) | 1.81  (0.58 to 3.7) | 1.02  (0.97 to 1.07) |
| Latvia | 29  (20 to 36) | 6.27  (4.32 to 7.82) | 35  (20 to 46) | 6.85  (3.94 to 9) | 0.2  (-0.15 to 0.63) | 0.7  (0.42 to 0.98) |
| Lebanon | 19  (13 to 25) | 6.72  (4.57 to 9.1) | 45  (31 to 61) | 7.1  (4.9 to 9.76) | 1.4  (0.7 to 2.54) | 0.13  (0.07 to 0.19) |
| Lesotho | 1  (0 to 1) | 0.46  (0.26 to 0.76) | 1  (0 to 2) | 0.65  (0.33 to 1.07) | 0.76  (0.05 to 2.2) | 1.23  (1.17 to 1.29) |
| Liberia | 2  (1 to 3) | 1.34  (0.73 to 1.94) | 3  (1 to 5) | 1.62  (0.68 to 2.57) | 0.84  (0.04 to 2.36) | 1.25  (0.93 to 1.57) |
| Libya | 13  (8 to 17) | 6.03  (4.05 to 8.33) | 32  (20 to 46) | 6.17  (3.75 to 8.7) | 1.58  (0.54 to 3.14) | 0.15  (0.02 to 0.28) |
| Lithuania | 40  (32 to 63) | 7.03  (5.49 to 10.92) | 81  (44 to 106) | 11.22  (6.16 to 14.69) | 1  (-0.21 to 2.03) | 2.39  (2.09 to 2.7) |
| Luxembourg | 6  (5 to 8) | 8.74  (7.69 to 10.88) | 13  (10 to 17) | 11.03  (8.25 to 13.92) | 1.15  (0.46 to 1.85) | 1.02  (0.67 to 1.38) |
| Madagascar | 5  (3 to 8) | 0.83  (0.52 to 1.39) | 12  (7 to 17) | 1.03  (0.65 to 1.56) | 1.38  (0.49 to 3.23) | 0.83  (0.8 to 0.87) |
| Malawi | 7  (5 to 10) | 1.57  (1.08 to 2.21) | 15  (10 to 21) | 1.85  (1.18 to 2.61) | 1.15  (0.47 to 2.27) | 0.62  (0.52 to 0.72) |
| Malaysia | 65  (50 to 84) | 6.31  (4.88 to 8.17) | 262  (184 to 349) | 8.1  (5.7 to 10.81) | 3.06  (1.8 to 4.83) | 0.88  (0.66 to 1.09) |
| Maldives | 1  (0 to 1) | 5.57  (3.25 to 8.63) | 2  (1 to 2) | 6.22  (4.59 to 7.98) | 2.56  (1.21 to 5.3) | 0.34  (0.25 to 0.44) |
| Mali | 3  (2 to 5) | 0.67  (0.44 to 0.99) | 8  (5 to 12) | 0.87  (0.56 to 1.28) | 1.54  (0.51 to 3.21) | 0.82  (0.78 to 0.87) |
| Malta | 6  (5 to 7) | 11.61  (9.94 to 13.78) | 15  (12 to 19) | 12.83  (10.19 to 15.98) | 1.46  (0.9 to 2.2) | 0.22  (-0.01 to 0.45) |
| Marshall Islands | 0  (0 to 0) | 3.3  (1.86 to 5.47) | 0  (0 to 0) | 3.22  (1.88 to 5.13) | 0.97  (0.25 to 2.14) | -0.21  (-0.31 to -0.11) |
| Mauritania | 2  (1 to 3) | 1.94  (1.06 to 2.93) | 5  (2 to 7) | 2.01  (0.88 to 3.01) | 1.17  (0.32 to 2.54) | 0.66  (0.42 to 0.9) |
| Mauritius | 1  (1 to 1) | 1.04  (0.89 to 1.26) | 5  (3 to 6) | 2.05  (1.54 to 2.58) | 3.93  (2.61 to 5.48) | 2.29  (1.78 to 2.79) |
| Mexico | 135  (125 to 171) | 2.76  (2.55 to 3.5) | 600  (505 to 718) | 4.32  (3.63 to 5.17) | 3.44  (2.69 to 4.2) | 1.51  (1.38 to 1.63) |
| Micronesia (Federated States of) | 0  (0 to 0) | 3.15  (1.8 to 5.06) | 0  (0 to 0) | 3.36  (1.76 to 5.42) | 0.52  (-0.12 to 1.48) | 0.09  (-0.04 to 0.22) |
| Monaco | 1  (1 to 2) | 13.36  (8.58 to 21.01) | 2  (2 to 4) | 20.95  (13.06 to 31.27) | 1.01  (0.25 to 2.1) | 1.67  (1.34 to 2.01) |
| Mongolia | 5  (3 to 7) | 4.32  (2.68 to 5.69) | 12  (6 to 17) | 5.27  (2.69 to 7.32) | 1.4  (0.56 to 2.54) | 0.64  (0.45 to 0.83) |
| Montenegro | 5  (3 to 7) | 6.1  (4.44 to 9.07) | 9  (7 to 12) | 7.03  (5.18 to 9.07) | 0.99  (0.26 to 2.06) | 0.53  (0.43 to 0.62) |
| Morocco | 37  (25 to 51) | 2.33  (1.54 to 3.15) | 118  (73 to 164) | 3.2  (1.99 to 4.46) | 2.14  (1.1 to 3.65) | 0.96  (0.88 to 1.04) |
| Mozambique | 6  (4 to 10) | 0.86  (0.53 to 1.47) | 16  (10 to 24) | 1.31  (0.87 to 2) | 1.69  (0.66 to 3.53) | 1.62  (1.57 to 1.67) |
| Myanmar | 51  (28 to 87) | 1.9  (1.04 to 3.21) | 168  (112 to 270) | 3.12  (2.07 to 5.01) | 2.29  (0.97 to 4.65) | 1.89  (1.77 to 2.02) |
| Namibia | 0  (0 to 1) | 0.48  (0.3 to 0.75) | 1  (1 to 2) | 0.62  (0.36 to 0.97) | 1.32  (0.42 to 3.05) | 0.96  (0.93 to 1) |
| Nauru | 0  (0 to 0) | 4.15  (2.52 to 6.47) | 0  (0 to 0) | 3.53  (2.24 to 5.27) | -0.07  (-0.42 to 0.5) | -1  (-1.2 to -0.81) |
| Nepal | 23  (13 to 36) | 2.2  (1.29 to 3.47) | 85  (56 to 122) | 3.17  (2.07 to 4.53) | 2.73  (1.39 to 5.06) | 1.27  (1.22 to 1.32) |
| Netherlands | 215  (193 to 263) | 8.5  (7.64 to 10.39) | 599  (454 to 770) | 14.01  (10.62 to 18.01) | 1.79  (1.06 to 2.74) | 2.11  (1.89 to 2.32) |
| New Zealand | 76  (68 to 90) | 14.92  (13.32 to 17.66) | 157  (126 to 194) | 16.01  (12.81 to 19.82) | 1.08  (0.63 to 1.61) | 0.03  (-0.21 to 0.26) |
| Nicaragua | 3  (2 to 5) | 1.83  (1.43 to 2.65) | 17  (12 to 21) | 3.26  (2.41 to 4.17) | 4.31  (2.46 to 6.54) | 2.07  (1.94 to 2.2) |
| Niger | 4  (2 to 5) | 1.15  (0.62 to 1.77) | 13  (6 to 20) | 1.55  (0.7 to 2.36) | 2.78  (1.33 to 5.32) | 1.19  (1.04 to 1.33) |
| Nigeria | 35  (25 to 57) | 0.69  (0.49 to 1.12) | 82  (58 to 132) | 0.9  (0.64 to 1.45) | 1.34  (0.68 to 2.27) | 1.39  (1.15 to 1.63) |
| Niue | 0  (0 to 0) | 3.97  (2.57 to 5.61) | 0  (0 to 0) | 4.11  (2.88 to 5.52) | 0.04  (-0.28 to 0.57) | 0.22  (0.18 to 0.26) |
| North Macedonia | 8  (6 to 12) | 3.75  (2.45 to 5.48) | 14  (10 to 20) | 3.38  (2.34 to 4.67) | 0.7  (0.12 to 1.52) | -0.49  (-0.57 to -0.41) |
| Northern Mariana Islands | 0  (0 to 0) | 7.77  (4.32 to 10.91) | 0  (0 to 0) | 3.49  (2.21 to 6.26) | 0.89  (0.03 to 4) | -3.29  (-3.72 to -2.87) |
| Norway | 71  (64 to 82) | 8.17  (7.31 to 9.36) | 98  (80 to 115) | 8.35  (6.8 to 9.77) | 0.38  (0.14 to 0.63) | 0.18  (0.12 to 0.23) |
| Oman | 3  (2 to 5) | 4.4  (2.72 to 6.59) | 8  (6 to 11) | 5.14  (3.68 to 6.95) | 1.7  (0.75 to 3.39) | 0.66  (0.35 to 0.97) |
| Pakistan | 153  (99 to 273) | 2.36  (1.53 to 4.21) | 340  (241 to 488) | 2.86  (2.02 to 4.1) | 1.22  (0.53 to 2.41) | 0.62  (0.54 to 0.69) |
| Palau | 0  (0 to 0) | 1.88  (1.27 to 2.74) | 0  (0 to 0) | 2.02  (1.36 to 2.89) | 1.23  (0.42 to 2.61) | 0.19  (0.12 to 0.26) |
| Palestine | 1  (0 to 2) | 0.85  (0.48 to 1.47) | 3  (2 to 5) | 1.38  (0.93 to 2.06) | 3  (1.38 to 6.17) | 1.56  (1.4 to 1.72) |
| Panama | 7  (5 to 9) | 4.07  (3.07 to 4.97) | 27  (19 to 37) | 5.55  (3.86 to 7.43) | 2.87  (1.81 to 4.44) | 1.14  (1.06 to 1.21) |
| Papua New Guinea | 4  (2 to 7) | 1.89  (0.96 to 3.41) | 11  (6 to 19) | 2.26  (1.19 to 3.91) | 1.76  (0.84 to 3.35) | 0.4  (0.25 to 0.55) |
| Paraguay | 6  (4 to 9) | 2.25  (1.74 to 3.42) | 34  (22 to 46) | 5.22  (3.41 to 6.99) | 4.86  (2.29 to 7.94) | 3.7  (3.37 to 4.03) |
| Peru | 38  (28 to 53) | 2.78  (2.06 to 3.89) | 162  (109 to 223) | 4.29  (2.89 to 5.9) | 3.3  (1.58 to 5.88) | 1.89  (1.59 to 2.18) |
| Philippines | 148  (84 to 179) | 4.49  (2.55 to 5.45) | 357  (276 to 451) | 4.01  (3.1 to 5.07) | 1.42  (0.83 to 3.26) | -1.17  (-1.52 to -0.82) |
| Poland | 326  (267 to 612) | 5.76  (4.72 to 10.8) | 1005  (532 to 1269) | 10.77  (5.7 to 13.58) | 2.09  (-0.06 to 3.51) | 2.56  (2.23 to 2.88) |
| Portugal | 103  (88 to 143) | 5.58  (4.78 to 7.76) | 327  (237 to 422) | 11.17  (8.1 to 14.42) | 2.17  (0.92 to 3.38) | 2.27  (1.93 to 2.61) |
| Puerto Rico | 23  (14 to 27) | 4.94  (3.14 to 5.98) | 53  (32 to 72) | 5.84  (3.59 to 8.02) | 1.32  (0.68 to 2.15) | 0.49  (0.28 to 0.7) |
| Qatar | 1  (0 to 1) | 8.03  (4.65 to 12.12) | 6  (4 to 8) | 6.62  (4.12 to 9.85) | 7.19  (3.75 to 13.05) | -0.68  (-0.97 to -0.39) |
| Republic of Korea | 72  (48 to 134) | 2.16  (1.44 to 4.03) | 615  (398 to 796) | 5.41  (3.5 to 7) | 7.59  (2.07 to 13.08) | 4.28  (3.73 to 4.83) |
| Republic of Moldova | 21  (18 to 29) | 3.79  (3.17 to 5.11) | 27  (22 to 31) | 3.54  (2.86 to 4.17) | 0.26  (-0.12 to 0.64) | 0.33  (-0.14 to 0.81) |
| Romania | 111  (92 to 163) | 3.06  (2.54 to 4.5) | 328  (260 to 407) | 6.66  (5.28 to 8.25) | 1.97  (1.01 to 2.95) | 3.29  (3.11 to 3.47) |
| Russian Federation | 754  (603 to 889) | 3.23  (2.58 to 3.81) | 869  (741 to 1179) | 2.79  (2.38 to 3.78) | 0.15  (-0.09 to 0.63) | -1.03  (-1.35 to -0.7) |
| Rwanda | 3  (2 to 5) | 0.8  (0.53 to 1.38) | 7  (5 to 12) | 1.09  (0.68 to 1.7) | 1.78  (0.56 to 3.56) | 1.21  (1.08 to 1.34) |
| Saint Kitts and Nevis | 0  (0 to 0) | 0.63  (0.51 to 0.83) | 0  (0 to 0) | 0.88  (0.69 to 1.12) | 1.17  (0.63 to 1.85) | 0.86  (0.6 to 1.11) |
| Saint Lucia | 0  (0 to 0) | 2.01  (1.63 to 2.77) | 1  (1 to 1) | 2.71  (2.15 to 3.34) | 2.09  (1.29 to 3.13) | 0.89  (0.73 to 1.05) |
| Saint Vincent and the Grenadines | 0  (0 to 0) | 0.98  (0.77 to 1.59) | 0  (0 to 0) | 2.31  (1.88 to 2.77) | 3.32  (1.87 to 4.88) | 2.8  (2.49 to 3.12) |
| Samoa | 0  (0 to 1) | 3.46  (2.32 to 4.89) | 1  (0 to 1) | 3.52  (2.45 to 5.1) | 0.61  (0.07 to 1.4) | 0.09  (0.04 to 0.13) |
| San Marino | 0  (0 to 1) | 9.85  (7.54 to 12.56) | 1  (1 to 1) | 12.9  (8.8 to 18.67) | 1.29  (0.55 to 2.55) | 1.22  (1.08 to 1.36) |
| Sao Tome and Principe | 0  (0 to 0) | 1.28  (0.68 to 1.86) | 0  (0 to 0) | 1.63  (0.74 to 2.39) | 0.78  (0.15 to 1.85) | 0.99  (0.92 to 1.05) |
| Saudi Arabia | 22  (9 to 33) | 3.64  (1.5 to 5.48) | 60  (32 to 85) | 3.74  (1.97 to 5.26) | 1.72  (0.61 to 3.72) | -0.36  (-0.6 to -0.11) |
| Senegal | 5  (3 to 8) | 1.37  (0.78 to 2.05) | 17  (8 to 25) | 2.02  (0.91 to 2.97) | 2.37  (1.06 to 4.45) | 1.73  (1.51 to 1.96) |
| Serbia | 44  (30 to 67) | 2.96  (2.02 to 4.55) | 101  (74 to 141) | 4.62  (3.39 to 6.42) | 1.32  (0.54 to 2.62) | 2.14  (1.82 to 2.46) |
| Seychelles | 0  (0 to 0) | 2.66  (1.43 to 3.88) | 0  (0 to 1) | 3.14  (1.56 to 4.63) | 1.14  (0.41 to 2.29) | 0.38  (0.27 to 0.48) |
| Sierra Leone | 2  (1 to 4) | 1.09  (0.61 to 1.63) | 6  (3 to 9) | 1.61  (0.67 to 2.41) | 1.56  (0.51 to 3.31) | 1.74  (1.58 to 1.89) |
| Singapore | 23  (20 to 26) | 8.91  (7.9 to 10.39) | 89  (70 to 110) | 8.99  (7.07 to 11.21) | 2.93  (2.01 to 4.06) | -0.02  (-0.33 to 0.29) |
| Slovakia | 79  (53 to 97) | 10.11  (6.77 to 12.49) | 128  (70 to 174) | 10.43  (5.7 to 14.19) | 0.62  (0.14 to 1.22) | 0.22  (0.03 to 0.4) |
| Slovenia | 17  (13 to 23) | 5.64  (4.27 to 7.34) | 73  (40 to 99) | 13.43  (7.34 to 18.15) | 3.19  (1.21 to 5.32) | 3.9  (3.41 to 4.4) |
| Solomon Islands | 0  (0 to 1) | 2.19  (1.11 to 4.17) | 1  (0 to 1) | 2.54  (1.43 to 4.47) | 1.41  (0.53 to 3.25) | 0.32  (0.19 to 0.46) |
| Somalia | 2  (1 to 3) | 0.68  (0.36 to 1.24) | 6  (2 to 11) | 0.83  (0.33 to 1.55) | 2.59  (0.8 to 5.41) | 1  (0.89 to 1.12) |
| South Africa | 8  (6 to 16) | 0.35  (0.23 to 0.67) | 25  (18 to 33) | 0.47  (0.35 to 0.63) | 1.95  (0.86 to 3.51) | 1.2  (1.07 to 1.34) |
| South Sudan | 4  (2 to 8) | 1.31  (0.6 to 2.87) | 6  (3 to 12) | 1.63  (0.83 to 2.95) | 0.82  (0.09 to 2.15) | 0.9  (0.83 to 0.97) |
| Spain | 317  (266 to 499) | 4.42  (3.7 to 6.95) | 1184  (863 to 1531) | 10.55  (7.69 to 13.63) | 2.73  (1.01 to 4.42) | 3.39  (3.1 to 3.67) |
| Sri Lanka | 37  (27 to 51) | 2.96  (2.18 to 4.13) | 134  (90 to 197) | 4.07  (2.75 to 5.98) | 2.65  (1.32 to 4.68) | 1.13  (1.03 to 1.23) |
| Sudan | 43  (21 to 75) | 4.01  (1.93 to 6.99) | 109  (57 to 176) | 5.49  (2.88 to 8.87) | 1.55  (0.69 to 3.15) | 1.13  (1.08 to 1.17) |
| Suriname | 0  (0 to 1) | 1.51  (1.1 to 2.19) | 2  (1 to 2) | 2.13  (1.55 to 2.89) | 2.32  (1.3 to 3.9) | 1.32  (1.21 to 1.44) |
| Sweden | 186  (167 to 222) | 9.72  (8.72 to 11.61) | 334  (271 to 400) | 13.2  (10.71 to 15.8) | 0.8  (0.45 to 1.18) | 0.88  (0.64 to 1.12) |
| Switzerland | 132  (110 to 156) | 10.21  (8.55 to 12.04) | 226  (162 to 301) | 10.97  (7.84 to 14.6) | 0.72  (0.18 to 1.33) | 0.77  (0.52 to 1.01) |
| Syrian Arab Republic | 53  (33 to 82) | 9.11  (5.54 to 14.01) | 134  (78 to 214) | 9.29  (5.41 to 14.84) | 1.5  (0.53 to 3.16) | -0.27  (-0.55 to 0.01) |
| Taiwan (Province of China) | 31  (25 to 45) | 1.57  (1.28 to 2.26) | 343  (135 to 485) | 6.71  (2.63 to 9.5) | 9.92  (2.49 to 16.2) | 8.14  (6.98 to 9.32) |
| Tajikistan | 6  (4 to 10) | 1.95  (1.32 to 2.97) | 16  (11 to 22) | 3.03  (2.08 to 4.15) | 1.47  (0.49 to 3.19) | 2.1  (1.76 to 2.43) |
| Thailand | 38  (27 to 54) | 0.95  (0.68 to 1.36) | 356  (136 to 533) | 2.8  (1.07 to 4.19) | 8.45  (2.86 to 15.49) | 4.83  (4.29 to 5.37) |
| Timor-Leste | 1  (0 to 1) | 1.93  (1.06 to 3.63) | 3  (2 to 5) | 2.91  (1.71 to 5.06) | 4.79  (2.63 to 8.44) | 1.71  (1.54 to 1.87) |
| Togo | 2  (1 to 2) | 1.28  (0.7 to 1.85) | 7  (3 to 11) | 1.91  (0.81 to 2.98) | 3.3  (1.52 to 6.45) | 1.54  (1.44 to 1.63) |
| Tokelau | 0  (0 to 0) | 3.05  (1.72 to 4.69) | 0  (0 to 0) | 3.15  (2.06 to 4.36) | -0.09  (-0.4 to 0.41) | -0.08  (-0.32 to 0.16) |
| Tonga | 0  (0 to 0) | 2.33  (1.47 to 3.55) | 0  (0 to 0) | 2.96  (2.03 to 4.11) | 0.78  (0.2 to 1.75) | 0.66  (0.44 to 0.88) |
| Trinidad and Tobago | 4  (3 to 4) | 3.72  (2.97 to 4.35) | 9  (7 to 12) | 3.97  (2.98 to 5.21) | 1.47  (0.82 to 2.33) | 0.25  (0.15 to 0.34) |
| Tunisia | 16  (11 to 23) | 2.58  (1.83 to 3.81) | 49  (33 to 76) | 3.17  (2.12 to 4.9) | 2.13  (0.96 to 4.03) | 0.7  (0.59 to 0.81) |
| Turkey | 350  (262 to 451) | 8.48  (6.36 to 10.93) | 1017  (769 to 1288) | 9.53  (7.21 to 12.07) | 1.91  (1.02 to 3.22) | 0.49  (0.43 to 0.55) |
| Turkmenistan | 4  (3 to 5) | 1.58  (1.18 to 2.3) | 11  (7 to 15) | 2.49  (1.74 to 3.56) | 2.04  (1.15 to 3.56) | 1.89  (1.68 to 2.1) |
| Tuvalu | 0  (0 to 0) | 2.9  (1.53 to 5.04) | 0  (0 to 0) | 3.17  (1.93 to 4.84) | 0.62  (0.04 to 1.54) | 0.09  (-0.07 to 0.25) |
| Uganda | 5  (3 to 7) | 0.69  (0.45 to 0.99) | 13  (9 to 18) | 0.87  (0.59 to 1.23) | 1.54  (0.56 to 2.97) | 0.9  (0.82 to 0.98) |
| Ukraine | 460  (368 to 596) | 4.84  (3.88 to 6.28) | 448  (351 to 569) | 4.46  (3.49 to 5.66) | -0.03  (-0.28 to 0.3) | -0.61  (-0.72 to -0.49) |
| United Arab Emirates | 3  (1 to 5) | 10.17  (4.59 to 16.69) | 25  (11 to 42) | 9.5  (4.18 to 15.75) | 7  (3.55 to 14.06) | -0.56  (-0.95 to -0.17) |
| United Kingdom | 1443  (1295 to 1589) | 12.3  (11.03 to 13.54) | 2340  (1858 to 2792) | 15.34  (12.18 to 18.31) | 0.62  (0.31 to 0.93) | 0.68  (0.54 to 0.81) |
| United Republic of Tanzania | 12  (7 to 21) | 0.96  (0.57 to 1.68) | 37  (24 to 57) | 1.4  (0.91 to 2.15) | 2.09  (0.99 to 4.23) | 1.57  (1.45 to 1.69) |
| United States of America | 5327  (4886 to 6123) | 13  (11.93 to 14.95) | 12147  (9810 to 14138) | 17.01  (13.74 to 19.8) | 1.28  (0.86 to 1.68) | 0.89  (0.69 to 1.08) |
| United States Virgin Islands | 0  (0 to 1) | 3.95  (2.75 to 5.23) | 1  (1 to 2) | 5.04  (3.72 to 6.42) | 2.31  (1.31 to 3.67) | 0.98  (0.87 to 1.09) |
| Uruguay | 31  (25 to 37) | 6.16  (4.93 to 7.27) | 48  (36 to 63) | 7.22  (5.33 to 9.48) | 0.54  (0.12 to 1.06) | 0.31  (0.13 to 0.5) |
| Uzbekistan | 38  (29 to 50) | 2.87  (2.17 to 3.75) | 78  (58 to 105) | 3.51  (2.62 to 4.72) | 1.05  (0.43 to 1.99) | 0.86  (0.68 to 1.05) |
| Vanuatu | 0  (0 to 0) | 2.51  (1.41 to 4.41) | 1  (0 to 1) | 2.92  (1.72 to 4.68) | 2.2  (1.06 to 4.07) | 0.29  (0.15 to 0.43) |
| Venezuela (Bolivarian Republic of) | 40  (35 to 49) | 3.64  (3.16 to 4.44) | 194  (147 to 255) | 5.5  (4.15 to 7.21) | 3.84  (2.52 to 5.67) | 1.46  (1.11 to 1.81) |
| Viet Nam | 214  (146 to 281) | 4.36  (2.98 to 5.72) | 618  (403 to 858) | 5.78  (3.77 to 8.03) | 1.89  (0.85 to 3.39) | 1.2  (1.02 to 1.37) |
| Yemen | 18  (10 to 29) | 3.33  (1.8 to 5.29) | 63  (29 to 101) | 4.36  (2.02 to 6.98) | 2.47  (1.12 to 4.46) | 1.18  (1.04 to 1.32) |
| Zambia | 4  (2 to 6) | 1.15  (0.72 to 1.97) | 10  (7 to 16) | 1.52  (1.02 to 2.28) | 1.94  (0.86 to 4.02) | 1.09  (1.01 to 1.17) |
| Zimbabwe | 4  (2 to 6) | 0.83  (0.52 to 1.35) | 6  (4 to 11) | 0.78  (0.47 to 1.35) | 0.57  (-0.08 to 1.6) | -0.42  (-0.59 to -0.24) |

| location | DALYs cases in 1990 | DALYs rate in 1990 | DALYs cases in 2019 | DALYs rate in 2019 | Cases change | EAPC |
| --- | --- | --- | --- | --- | --- | --- |
| Afghanistan | 923  (483 to 1588) | 107.13  (56.08 to 184.28) | 1138  (561 to 1863) | 105.58  (52.05 to 172.85) | 0.23  (-0.26 to 0.99) | 0.03  (-0.1 to 0.16) |
| Albania | 266  (193 to 409) | 111.4  (81.13 to 171.42) | 570  (362 to 953) | 100.88  (64.04 to 168.76) | 1.15  (0.43 to 2.23) | -0.29  (-0.41 to -0.17) |
| Algeria | 954  (640 to 1329) | 65.02  (43.63 to 90.55) | 2567  (1666 to 3586) | 65.32  (42.4 to 91.25) | 1.69  (0.77 to 3.17) | 0.06  (-0.01 to 0.12) |
| American Samoa | 1  (1 to 2) | 57.19  (40.86 to 74.85) | 2  (2 to 3) | 41.78  (30.54 to 55.88) | 0.63  (0.08 to 1.51) | -1.29  (-1.45 to -1.13) |
| Andorra | 23  (16 to 32) | 319.13  (218.33 to 451.84) | 51  (35 to 68) | 305.24  (207.77 to 411.69) | 1.24  (0.46 to 2.34) | -0.21  (-0.23 to -0.19) |
| Angola | 99  (60 to 161) | 24.52  (14.71 to 39.88) | 335  (199 to 523) | 29.19  (17.38 to 45.64) | 2.37  (1.03 to 4.76) | 0.74  (0.62 to 0.85) |
| Antigua and Barbuda | 3  (2 to 4) | 45.35  (35.46 to 58.27) | 9  (7 to 11) | 71.74  (54.2 to 92.79) | 1.88  (1.03 to 3.16) | 1.88  (1.66 to 2.09) |
| Argentina | 3740  (3063 to 4449) | 91.02  (74.54 to 108.27) | 8036  (6634 to 9519) | 118.81  (98.08 to 140.74) | 1.15  (0.71 to 1.73) | 0.78  (0.56 to 1.01) |
| Armenia | 129  (93 to 178) | 39.41  (28.43 to 54.28) | 361  (217 to 478) | 65.92  (39.66 to 87.23) | 1.8  (0.6 to 3.33) | 1.63  (1.22 to 2.03) |
| Australia | 6331  (5433 to 6860) | 250.06  (214.61 to 270.95) | 13603  (10646 to 15627) | 261.95  (204.99 to 300.9) | 1.15  (0.79 to 1.46) | -0.01  (-0.14 to 0.13) |
| Austria | 2041  (1813 to 2528) | 133.36  (118.5 to 165.19) | 4949  (3266 to 5763) | 232.61  (153.53 to 270.89) | 1.42  (0.55 to 1.99) | 2.41  (2.14 to 2.69) |
| Azerbaijan | 226  (153 to 348) | 40.33  (27.33 to 62) | 589  (386 to 914) | 55.38  (36.25 to 85.89) | 1.6  (0.72 to 2.86) | 1.2  (1.05 to 1.35) |
| Bahamas | 11  (9 to 13) | 62.82  (53.5 to 77.76) | 31  (25 to 40) | 68.82  (54.49 to 87.42) | 1.88  (1.17 to 2.77) | 0.59  (0.49 to 0.69) |
| Bahrain | 34  (21 to 50) | 186.8  (116.27 to 275.97) | 151  (101 to 209) | 144.52  (96.04 to 200.11) | 3.51  (1.77 to 6.65) | -1.74  (-2.08 to -1.4) |
| Bangladesh | 2891  (1826 to 4224) | 55.4  (34.99 to 80.95) | 8634  (5257 to 13862) | 55.21  (33.61 to 88.64) | 1.99  (0.84 to 3.84) | -0.13  (-0.23 to -0.03) |
| Barbados | 34  (29 to 41) | 89.35  (76.89 to 109.94) | 79  (63 to 94) | 123.22  (99.03 to 147.97) | 1.34  (0.81 to 1.96) | 1.35  (1.14 to 1.57) |
| Belarus | 2979  (2232 to 3547) | 177.29  (132.86 to 211.1) | 3223  (2242 to 4334) | 155.13  (107.92 to 208.61) | 0.08  (-0.23 to 0.5) | -0.64  (-0.74 to -0.55) |
| Belgium | 3065  (2634 to 3959) | 153.26  (131.73 to 197.98) | 6170  (4851 to 7139) | 223.31  (175.58 to 258.39) | 1.01  (0.38 to 1.49) | 1.09  (0.85 to 1.33) |
| Belize | 1  (1 to 1) | 9.04  (6.46 to 12.61) | 4  (3 to 5) | 13.3  (9.96 to 17.47) | 3.06  (1.76 to 5.15) | 1.29  (1.12 to 1.46) |
| Benin | 60  (35 to 87) | 25.94  (15.08 to 37.73) | 179  (87 to 259) | 34.18  (16.61 to 49.68) | 1.97  (0.82 to 3.84) | 1.1  (1.01 to 1.19) |
| Bermuda | 8  (6 to 10) | 106.21  (82.82 to 135.35) | 15  (12 to 19) | 88.69  (69.81 to 112.13) | 0.79  (0.31 to 1.47) | -0.64  (-0.75 to -0.53) |
| Bhutan | 14  (8 to 23) | 53.48  (31.21 to 87.03) | 49  (29 to 84) | 74.14  (43.92 to 126.71) | 2.46  (1.09 to 4.68) | 1.18  (1.09 to 1.28) |
| Bolivia (Plurinational State of) | 244  (157 to 364) | 66.03  (42.52 to 98.56) | 1124  (739 to 1614) | 105.78  (69.54 to 151.88) | 3.61  (2.01 to 6.49) | 1.67  (1.58 to 1.76) |
| Bosnia and Herzegovina | 420  (283 to 702) | 87.78  (59.23 to 146.78) | 997  (689 to 1331) | 125.11  (86.45 to 167.07) | 1.37  (0.24 to 3.03) | 1.42  (1.22 to 1.62) |
| Botswana | 10  (5 to 16) | 14.28  (7.85 to 24.51) | 27  (13 to 47) | 17.99  (8.71 to 31.24) | 1.84  (0.54 to 4.06) | 0.42  (0.19 to 0.66) |
| Brazil | 9114  (8145 to 10323) | 87.69  (78.37 to 99.33) | 31661  (25840 to 34533) | 109.89  (89.69 to 119.86) | 2.47  (1.92 to 2.81) | 0.93  (0.83 to 1.02) |
| Brunei Darussalam | 7  (4 to 10) | 71.67  (44.92 to 104.05) | 21  (15 to 29) | 64.29  (46.82 to 88.87) | 1.96  (0.88 to 4.12) | -0.45  (-0.68 to -0.23) |
| Bulgaria | 1624  (1261 to 1971) | 96.71  (75.12 to 117.38) | 2561  (1928 to 3348) | 132.56  (99.83 to 173.34) | 0.58  (0.14 to 1.16) | 1.77  (1.51 to 2.04) |
| Burkina Faso | 124  (68 to 190) | 24.14  (13.3 to 37.04) | 321  (145 to 476) | 32.49  (14.69 to 48.14) | 1.59  (0.57 to 3.3) | 1.13  (0.88 to 1.38) |
| Burundi | 42  (26 to 72) | 15.86  (9.58 to 26.95) | 113  (61 to 194) | 23.04  (12.36 to 39.57) | 1.67  (0.29 to 3.87) | 1.47  (1.37 to 1.57) |
| Cabo Verde | 7  (4 to 10) | 23.29  (12.54 to 34.06) | 23  (9 to 33) | 46.61  (19.58 to 68.8) | 2.33  (1 to 4.31) | 2.3  (1.78 to 2.83) |
| Cambodia | 207  (119 to 353) | 41.43  (23.81 to 70.64) | 767  (475 to 1276) | 56.02  (34.69 to 93.23) | 2.71  (1.32 to 5) | 1.09  (0.99 to 1.18) |
| Cameroon | 179  (104 to 274) | 35.89  (20.79 to 54.96) | 563  (252 to 868) | 44.11  (19.71 to 67.98) | 2.15  (0.83 to 4.45) | 0.93  (0.84 to 1.02) |
| Canada | 6382  (5706 to 8249) | 153.91  (137.62 to 198.94) | 17456  (14413 to 20080) | 199.66  (164.86 to 229.67) | 1.74  (1.05 to 2.31) | 1.32  (1.11 to 1.53) |
| Central African Republic | 31  (18 to 51) | 24.81  (14.71 to 40.84) | 49  (26 to 89) | 23.01  (12.06 to 41.72) | 0.59  (-0.13 to 1.99) | -0.19  (-0.27 to -0.11) |
| Chad | 75  (41 to 115) | 22.76  (12.51 to 34.71) | 191  (88 to 278) | 31.53  (14.47 to 45.95) | 1.54  (0.6 to 3.16) | 1.44  (1.32 to 1.57) |
| Chile | 883  (735 to 1185) | 73.2  (60.95 to 98.25) | 3313  (2811 to 3873) | 110.57  (93.81 to 129.26) | 2.75  (1.85 to 3.7) | 1.66  (1.49 to 1.83) |
| China | 19534  (14293 to 27358) | 19.42  (14.21 to 27.19) | 71247  (57106 to 88543) | 28.03  (22.46 to 34.83) | 2.65  (1.47 to 4.37) | 1.56  (1.44 to 1.68) |
| Colombia | 1085  (927 to 1486) | 54.52  (46.59 to 74.69) | 5270  (3764 to 6961) | 82.55  (58.96 to 109.05) | 3.86  (2.18 to 6) | 1.53  (1.37 to 1.69) |
| Comoros | 5  (3 to 10) | 21  (10.69 to 39.61) | 14  (9 to 23) | 25.62  (15.94 to 41.71) | 1.61  (0.46 to 3.96) | 0.73  (0.65 to 0.8) |
| Congo | 39  (25 to 64) | 32.27  (20.45 to 52.15) | 90  (56 to 132) | 33.62  (20.88 to 49.14) | 1.29  (0.37 to 3.18) | 0.08  (-0.08 to 0.24) |
| Cook Islands | 1  (1 to 1) | 58.14  (41.8 to 77.65) | 2  (1 to 2) | 53.53  (39.46 to 71.26) | 0.93  (0.31 to 1.88) | -0.46  (-0.53 to -0.39) |
| Costa Rica | 230  (185 to 276) | 112.33  (90.34 to 135.01) | 836  (609 to 1125) | 134.08  (97.69 to 180.27) | 2.64  (1.53 to 4.06) | 0.71  (0.55 to 0.88) |
| C么te d'Ivoire | 121  (69 to 192) | 29.16  (16.57 to 46.29) | 379  (181 to 570) | 34.62  (16.55 to 51.97) | 2.14  (0.92 to 4.37) | 0.66  (0.6 to 0.72) |
| Croatia | 431  (282 to 1024) | 52.35  (34.24 to 124.48) | 1967  (1015 to 2712) | 171.93  (88.71 to 237.01) | 3.57  (0.17 to 7.44) | 4.49  (3.91 to 5.08) |
| Cuba | 1017  (860 to 1309) | 80.24  (67.84 to 103.25) | 2109  (1632 to 2747) | 93.23  (72.15 to 121.44) | 1.07  (0.6 to 1.66) | 0.87  (0.71 to 1.04) |
| Cyprus | 169  (128 to 215) | 158.02  (119.66 to 200.52) | 555  (266 to 732) | 220.1  (105.78 to 290.39) | 2.28  (0.7 to 3.82) | 1.79  (1.48 to 2.1) |
| Czechia | 3161  (2776 to 3882) | 174.06  (152.86 to 213.72) | 5090  (3848 to 6415) | 186.9  (141.31 to 235.56) | 0.61  (0.11 to 1.12) | 0.87  (0.63 to 1.11) |
| Democratic People's Republic of Korea | 489  (302 to 766) | 27.65  (17.09 to 43.33) | 1079  (620 to 1650) | 28.21  (16.21 to 43.15) | 1.21  (0.37 to 2.65) | 0.21  (0.15 to 0.28) |
| Democratic Republic of the Congo | 417  (255 to 653) | 23.34  (14.28 to 36.51) | 884  (449 to 1581) | 23.64  (12.02 to 42.3) | 1.12  (0.07 to 2.78) | -0.01  (-0.31 to 0.29) |
| Denmark | 3448  (2877 to 3776) | 336.23  (280.55 to 368.22) | 3657  (3020 to 4220) | 255.37  (210.87 to 294.7) | 0.06  (-0.09 to 0.24) | -0.6  (-0.87 to -0.32) |
| Djibouti | 3  (2 to 6) | 22.66  (11.23 to 44.52) | 20  (12 to 35) | 32.93  (18.9 to 56.54) | 5.5  (2.91 to 10.82) | 1.45  (1.36 to 1.54) |
| Dominica | 5  (4 to 8) | 56.48  (39.49 to 80.14) | 8  (5 to 11) | 69.68  (48.25 to 97.32) | 0.46  (-0.04 to 1.29) | 0.99  (0.89 to 1.09) |
| Dominican Republic | 86  (57 to 153) | 19.99  (13.31 to 35.55) | 511  (318 to 754) | 46.78  (29.14 to 69.08) | 4.94  (1.91 to 9.81) | 3.72  (3.5 to 3.95) |
| Ecuador | 343  (253 to 518) | 55.64  (41.11 to 84.21) | 2037  (1304 to 2722) | 111.77  (71.52 to 149.33) | 4.95  (1.84 to 8.29) | 3.22  (2.86 to 3.58) |
| Egypt | 1596  (987 to 2556) | 48.68  (30.1 to 77.97) | 4316  (2431 to 7237) | 59.55  (33.53 to 99.84) | 1.7  (0.62 to 3.26) | 0.67  (0.54 to 0.79) |
| El Salvador | 45  (34 to 72) | 13.03  (9.79 to 20.93) | 296  (134 to 426) | 41.8  (18.98 to 60.2) | 5.61  (1.48 to 10.16) | 5.05  (4.39 to 5.72) |
| Equatorial Guinea | 5  (3 to 8) | 21.51  (11.94 to 36.64) | 19  (10 to 34) | 37.46  (19.22 to 68) | 3.02  (0.82 to 7.89) | 2.59  (2.35 to 2.82) |
| Eritrea | 15  (8 to 25) | 15.47  (8.49 to 26.19) | 65  (41 to 99) | 24.34  (15.29 to 36.86) | 3.47  (1.56 to 7.28) | 1.39  (1.15 to 1.63) |
| Estonia | 322  (219 to 657) | 122.08  (82.81 to 249.02) | 629  (469 to 826) | 187.88  (140.22 to 246.64) | 0.95  (-0.02 to 2.14) | 2.2  (1.64 to 2.76) |
| Eswatini | 5  (3 to 8) | 14.86  (8.39 to 24.46) | 11  (5 to 18) | 17.25  (7.68 to 27.91) | 1.42  (0.36 to 3.48) | 0.51  (0.22 to 0.79) |
| Ethiopia | 2134  (923 to 3773) | 97.54  (42.17 to 172.45) | 4685  (2058 to 7253) | 104.11  (45.74 to 161.19) | 1.2  (0.41 to 2.69) | 0.26  (0.18 to 0.33) |
| Fiji | 65  (47 to 87) | 175.98  (127.36 to 235.33) | 165  (120 to 222) | 191.31  (139.24 to 256.72) | 1.55  (0.69 to 2.92) | 0.46  (0.33 to 0.6) |
| Finland | 1792  (1602 to 2006) | 195.29  (174.64 to 218.64) | 2688  (2318 to 3138) | 174.94  (150.89 to 204.27) | 0.5  (0.28 to 0.77) | -0.41  (-0.51 to -0.31) |
| France | 13992  (12563 to 17241) | 131.77  (118.31 to 162.37) | 32200  (23013 to 37646) | 195.88  (140 to 229.01) | 1.3  (0.6 to 1.8) | 1.65  (1.5 to 1.8) |
| Gabon | 26  (13 to 49) | 37.89  (19.39 to 72.44) | 48  (27 to 78) | 41.89  (23.15 to 67.6) | 0.89  (0.02 to 2.65) | 0.24  (0.17 to 0.3) |
| Gambia | 5  (3 to 7) | 11.67  (6.77 to 18.2) | 16  (10 to 24) | 15.05  (9.63 to 23.01) | 2.53  (1 to 5.71) | 0.75  (0.54 to 0.95) |
| Georgia | 372  (254 to 645) | 46.8  (31.95 to 81.15) | 567  (337 to 768) | 73.36  (43.58 to 99.4) | 0.52  (-0.41 to 1.58) | 2.53  (1.18 to 3.89) |
| Germany | 21256  (18001 to 24125) | 133.07  (112.69 to 151.04) | 53345  (36352 to 62153) | 231.9  (158.03 to 270.19) | 1.51  (0.86 to 2.01) | 2.55  (2.37 to 2.74) |
| Ghana | 284  (185 to 424) | 41.27  (26.85 to 61.6) | 845  (605 to 1176) | 47.48  (34 to 66.14) | 1.97  (0.88 to 3.98) | 0.36  (0.3 to 0.42) |
| Greece | 2871  (2516 to 3238) | 145.13  (127.2 to 163.68) | 7082  (4549 to 8174) | 251.17  (161.34 to 289.93) | 1.47  (0.67 to 1.88) | 2  (1.85 to 2.14) |
| Greenland | 3  (3 to 4) | 94.1  (71.04 to 118.68) | 7  (5 to 11) | 87.98  (62.77 to 126.59) | 1.17  (0.46 to 2.01) | 0.08  (-0.09 to 0.25) |
| Grenada | 3  (2 to 4) | 30.07  (22.59 to 38.9) | 6  (5 to 8) | 44.66  (34.63 to 61.01) | 1.14  (0.5 to 2.05) | 1.73  (1.56 to 1.89) |
| Guam | 10  (7 to 13) | 111.07  (76.76 to 145.04) | 11  (7 to 21) | 46.48  (28.23 to 91.13) | 0.1  (-0.38 to 1.7) | -3.34  (-3.55 to -3.13) |
| Guatemala | 45  (32 to 72) | 10.85  (7.71 to 17.3) | 491  (339 to 644) | 37.49  (25.86 to 49.15) | 9.81  (4.56 to 16.3) | 5.35  (4.93 to 5.78) |
| Guinea | 68  (43 to 100) | 16.83  (10.81 to 24.92) | 120  (76 to 186) | 19.23  (12.2 to 29.83) | 0.77  (0.04 to 2.08) | 0.32  (0.24 to 0.4) |
| Guinea-Bissau | 13  (8 to 20) | 29.17  (16.46 to 44.06) | 29  (12 to 44) | 37.87  (16.51 to 58.1) | 1.15  (0.25 to 2.63) | 1.1  (1.02 to 1.18) |
| Guyana | 7  (5 to 10) | 16.73  (11.38 to 22.58) | 12  (8 to 17) | 16.14  (10.64 to 23.79) | 0.62  (-0.04 to 1.85) | -0.19  (-0.41 to 0.03) |
| Haiti | 156  (83 to 273) | 41.7  (22.14 to 72.81) | 368  (180 to 645) | 47.28  (23.17 to 82.83) | 1.36  (0.44 to 2.77) | 0.53  (0.49 to 0.58) |
| Honduras | 147  (100 to 204) | 62.95  (42.79 to 87.39) | 704  (477 to 984) | 99.55  (67.36 to 139.06) | 3.79  (2 to 6.37) | 1.79  (1.7 to 1.88) |
| Hungary | 3006  (2674 to 3429) | 154.5  (137.42 to 176.23) | 5310  (3980 to 6622) | 208.05  (155.96 to 259.49) | 0.77  (0.31 to 1.2) | 1.08  (0.74 to 1.42) |
| Iceland | 59  (50 to 68) | 164.79  (139.56 to 190.04) | 113  (91 to 147) | 164.86  (132.88 to 214.24) | 0.91  (0.51 to 1.49) | 0.02  (-0.1 to 0.13) |
| India | 25434  (19393 to 32366) | 51.05  (38.92 to 64.96) | 83117  (68562 to 104207) | 60.78  (50.14 to 76.21) | 2.27  (1.44 to 3.41) | 0.45  (0.37 to 0.53) |
| Indonesia | 4520  (3381 to 7612) | 41.94  (31.37 to 70.62) | 14883  (10257 to 24352) | 60.86  (41.94 to 99.58) | 2.29  (1.44 to 3.44) | 1.21  (1.15 to 1.26) |
| Iran (Islamic Republic of) | 3294  (2572 to 4257) | 107.68  (84.06 to 139.16) | 8819  (7283 to 12219) | 104.98  (86.69 to 145.46) | 1.68  (1.11 to 2.37) | -0.06  (-0.15 to 0.04) |
| Iraq | 516  (315 to 873) | 59.06  (36.14 to 99.99) | 1662  (1069 to 2655) | 66.97  (43.05 to 106.98) | 2.22  (1.08 to 4.32) | 0.41  (0.25 to 0.57) |
| Ireland | 902  (790 to 1124) | 170.12  (148.96 to 211.89) | 1887  (1475 to 2192) | 202.51  (158.38 to 235.31) | 1.09  (0.5 to 1.54) | 0.67  (0.56 to 0.79) |
| Israel | 1478  (1203 to 1689) | 234.6  (190.97 to 268.14) | 3970  (2934 to 4591) | 273.83  (202.39 to 316.66) | 1.69  (1.2 to 2.25) | 0.48  (0.32 to 0.65) |
| Italy | 14013  (12584 to 21040) | 120.4  (108.12 to 180.77) | 35419  (27552 to 38564) | 210.22  (163.53 to 228.89) | 1.53  (0.54 to 1.95) | 2.29  (1.97 to 2.6) |
| Jamaica | 52  (40 to 71) | 23.33  (17.9 to 31.56) | 205  (146 to 277) | 57.98  (41.29 to 78.25) | 2.93  (1.45 to 4.84) | 3.35  (2.86 to 3.84) |
| Japan | 26772  (24322 to 29675) | 124.45  (113.06 to 137.94) | 66743  (53948 to 72293) | 161.22  (130.31 to 174.62) | 1.49  (1.03 to 1.68) | 1.04  (0.84 to 1.23) |
| Jordan | 24  (16 to 35) | 17.47  (11.65 to 24.98) | 160  (97 to 226) | 23.21  (14.05 to 32.78) | 5.6  (2.95 to 10.44) | 1.02  (0.9 to 1.14) |
| Kazakhstan | 1103  (855 to 1556) | 72.66  (56.32 to 102.55) | 1725  (1304 to 2314) | 84.11  (63.57 to 112.83) | 0.56  (0.17 to 1.11) | 1.45  (1.1 to 1.8) |
| Kenya | 214  (131 to 359) | 23.12  (14.16 to 38.7) | 759  (538 to 989) | 32.16  (22.78 to 41.92) | 2.54  (1.51 to 4.03) | 1.33  (1.26 to 1.41) |
| Kiribati | 1  (1 to 2) | 27.6  (14.36 to 42.96) | 2  (1 to 3) | 26.6  (13.2 to 42.05) | 0.72  (0.02 to 1.95) | -0.31  (-0.4 to -0.22) |
| Kuwait | 27  (19 to 38) | 47.02  (33.48 to 66.24) | 157  (112 to 207) | 65.85  (47.19 to 87.02) | 4.74  (2.81 to 7.82) | 1.9  (1.26 to 2.55) |
| Kyrgyzstan | 154  (120 to 221) | 41.51  (32.32 to 59.41) | 306  (224 to 376) | 60.53  (44.36 to 74.41) | 0.99  (0.19 to 1.76) | 2  (1.41 to 2.59) |
| Lao People's Democratic Republic | 103  (61 to 176) | 43.43  (25.75 to 74.13) | 264  (159 to 452) | 55.1  (33.08 to 94.2) | 1.56  (0.4 to 3.35) | 0.69  (0.62 to 0.75) |
| Latvia | 576  (403 to 722) | 124.94  (87.48 to 156.62) | 597  (358 to 791) | 118.04  (70.79 to 156.37) | 0.04  (-0.26 to 0.43) | 0.08  (-0.2 to 0.36) |
| Lebanon | 370  (251 to 505) | 133.97  (90.97 to 182.69) | 776  (534 to 1080) | 123.54  (85.06 to 171.82) | 1.1  (0.47 to 2.13) | -0.4  (-0.48 to -0.32) |
| Lesotho | 12  (6 to 20) | 10.05  (5.49 to 16.75) | 20  (10 to 34) | 13.81  (6.98 to 23.2) | 0.72  (-0.01 to 2.21) | 1.19  (1.14 to 1.23) |
| Liberia | 37  (20 to 53) | 26.84  (14.7 to 38.94) | 62  (26 to 99) | 29.82  (12.58 to 47.64) | 0.69  (-0.03 to 2.07) | 0.95  (0.6 to 1.3) |
| Libya | 248  (166 to 350) | 119.64  (80.03 to 168.37) | 596  (367 to 842) | 113.83  (70.07 to 160.69) | 1.4  (0.41 to 2.88) | -0.14  (-0.24 to -0.05) |
| Lithuania | 809  (625 to 1258) | 140.73  (108.73 to 218.91) | 1367  (781 to 1808) | 189.74  (108.37 to 250.9) | 0.69  (-0.31 to 1.55) | 1.67  (1.37 to 1.98) |
| Luxembourg | 112  (98 to 141) | 159.21  (139.84 to 199.86) | 221  (171 to 263) | 185.18  (142.61 to 219.51) | 0.98  (0.38 to 1.46) | 0.64  (0.35 to 0.93) |
| Madagascar | 100  (63 to 170) | 17.13  (10.67 to 28.98) | 234  (147 to 364) | 20.98  (13.16 to 32.61) | 1.33  (0.4 to 3.14) | 0.77  (0.7 to 0.84) |
| Malawi | 146  (99 to 205) | 33.03  (22.45 to 46.22) | 299  (190 to 425) | 36.99  (23.48 to 52.6) | 1.04  (0.35 to 2.23) | 0.46  (0.4 to 0.53) |
| Malaysia | 1260  (966 to 1633) | 123.21  (94.44 to 159.72) | 4842  (3371 to 6478) | 149.79  (104.26 to 200.36) | 2.84  (1.66 to 4.62) | 0.72  (0.53 to 0.91) |
| Maldives | 11  (6 to 17) | 112.64  (63.71 to 177.89) | 33  (25 to 43) | 106.93  (79.27 to 137.84) | 2.02  (0.86 to 4.44) | -0.4  (-0.53 to -0.27) |
| Mali | 67  (43 to 101) | 13.99  (8.98 to 20.92) | 160  (101 to 239) | 16.76  (10.58 to 25.05) | 1.36  (0.33 to 2.98) | 0.59  (0.53 to 0.64) |
| Malta | 116  (99 to 135) | 213  (183.02 to 249.08) | 245  (203 to 293) | 203.34  (167.79 to 242.3) | 1.12  (0.7 to 1.66) | -0.19  (-0.38 to 0) |
| Marshall Islands | 1  (1 to 2) | 66.42  (37.16 to 112.31) | 2  (1 to 4) | 63.28  (36.37 to 101.29) | 0.92  (0.18 to 2.23) | -0.11  (-0.19 to -0.04) |
| Mauritania | 44  (24 to 66) | 38.34  (21.17 to 57.58) | 87  (39 to 130) | 36.37  (16.03 to 54.02) | 0.98  (0.21 to 2.26) | 0.33  (0.1 to 0.56) |
| Mauritius | 18  (16 to 22) | 20.7  (17.45 to 24.92) | 83  (61 to 106) | 37.28  (27.28 to 47.38) | 3.52  (2.26 to 5.01) | 2.01  (1.47 to 2.56) |
| Mexico | 2623  (2441 to 3295) | 53.56  (49.86 to 67.28) | 10694  (8896 to 12805) | 76.96  (64.03 to 92.16) | 3.08  (2.34 to 3.83) | 1.18  (1.09 to 1.27) |
| Micronesia (Federated States of) | 4  (2 to 6) | 63.97  (36.08 to 105.61) | 5  (3 to 9) | 66.48  (33.39 to 111.38) | 0.48  (-0.19 to 1.46) | 0.04  (0 to 0.08) |
| Monaco | 21  (13 to 33) | 230.07  (146.35 to 362.03) | 38  (24 to 56) | 327.67  (206.48 to 483.6) | 0.82  (0.15 to 1.81) | 1.35  (1.1 to 1.6) |
| Mongolia | 110  (67 to 146) | 92.06  (56.46 to 122.7) | 251  (127 to 352) | 107.23  (54.33 to 150.2) | 1.29  (0.49 to 2.41) | 0.4  (0.24 to 0.56) |
| Montenegro | 90  (65 to 133) | 118.69  (86.45 to 176.11) | 167  (122 to 215) | 127.26  (93.05 to 163.96) | 0.85  (0.18 to 1.87) | 0.16  (0.07 to 0.25) |
| Morocco | 752  (488 to 1036) | 46.8  (30.35 to 64.51) | 2287  (1388 to 3188) | 62.22  (37.77 to 86.73) | 2.04  (1 to 3.57) | 0.81  (0.67 to 0.96) |
| Mozambique | 120  (71 to 212) | 17.88  (10.66 to 31.62) | 307  (200 to 479) | 25.96  (16.89 to 40.47) | 1.56  (0.6 to 3.39) | 1.5  (1.41 to 1.58) |
| Myanmar | 1040  (553 to 1773) | 38.47  (20.47 to 65.62) | 3084  (1983 to 4942) | 57.18  (36.77 to 91.64) | 1.97  (0.75 to 4.21) | 1.48  (1.42 to 1.53) |
| Namibia | 9  (6 to 15) | 10.25  (6.4 to 16.42) | 20  (11 to 32) | 12.58  (7.09 to 19.93) | 1.19  (0.29 to 2.92) | 0.78  (0.73 to 0.83) |
| Nauru | 0  (0 to 0) | 83.88  (48.95 to 130.98) | 0  (0 to 0) | 71.65  (44.41 to 109.04) | -0.07  (-0.43 to 0.56) | -0.91  (-1.07 to -0.75) |
| Nepal | 473  (274 to 739) | 45.54  (26.37 to 71.11) | 1607  (1037 to 2275) | 59.78  (38.57 to 84.63) | 2.39  (1.13 to 4.56) | 0.98  (0.89 to 1.07) |
| Netherlands | 3929  (3532 to 4763) | 155.48  (139.77 to 188.45) | 9612  (8102 to 11026) | 224.74  (189.43 to 257.79) | 1.45  (0.97 to 1.88) | 1.63  (1.43 to 1.84) |
| New Zealand | 1221  (1101 to 1430) | 240.73  (217.07 to 282.01) | 2444  (2106 to 2762) | 249.35  (214.87 to 281.78) | 1  (0.69 to 1.32) | -0.12  (-0.32 to 0.09) |
| Nicaragua | 62  (48 to 88) | 36.33  (28.29 to 51.76) | 306  (224 to 394) | 59.93  (43.81 to 77.15) | 3.93  (2.17 to 6.01) | 1.82  (1.72 to 1.92) |
| Niger | 73  (39 to 113) | 23.68  (12.75 to 36.92) | 248  (112 to 380) | 28.9  (13.11 to 44.3) | 2.42  (1.07 to 4.72) | 0.85  (0.68 to 1.02) |
| Nigeria | 713  (498 to 1153) | 14.13  (9.87 to 22.85) | 1545  (1097 to 2435) | 16.98  (12.06 to 26.74) | 1.17  (0.52 to 2.12) | 1.1  (0.85 to 1.35) |
| Niue | 0  (0 to 0) | 70.16  (44.96 to 101.19) | 0  (0 to 0) | 70.83  (48.98 to 96.91) | 0.02  (-0.31 to 0.6) | 0.02  (-0.01 to 0.04) |
| North Macedonia | 170  (112 to 241) | 75.29  (49.75 to 106.47) | 270  (184 to 381) | 63.63  (43.37 to 89.66) | 0.59  (0.06 to 1.37) | -0.77  (-0.85 to -0.69) |
| Northern Mariana Islands | 2  (1 to 3) | 153.9  (86.08 to 222.4) | 4  (3 to 8) | 69.05  (43.56 to 123.67) | 0.88  (0.01 to 3.85) | -3.2  (-3.51 to -2.89) |
| Norway | 1250  (1122 to 1417) | 143.05  (128.46 to 162.15) | 1784  (1520 to 1927) | 151.4  (128.99 to 163.52) | 0.43  (0.24 to 0.55) | 0.43  (0.3 to 0.55) |
| Oman | 65  (39 to 99) | 91.58  (55.28 to 140.19) | 161  (115 to 218) | 99.21  (70.56 to 134.21) | 1.5  (0.63 to 3.1) | 0.41  (0.16 to 0.66) |
| Pakistan | 2981  (1945 to 5284) | 45.94  (29.97 to 81.41) | 6509  (4601 to 9630) | 54.65  (38.63 to 80.85) | 1.18  (0.52 to 2.25) | 0.58  (0.52 to 0.63) |
| Palau | 0  (0 to 1) | 38.86  (25.95 to 56.91) | 1  (1 to 1) | 40.23  (26.35 to 58.47) | 1.15  (0.31 to 2.55) | 0.11  (0.08 to 0.15) |
| Palestine | 18  (10 to 31) | 17.61  (9.72 to 30.57) | 69  (46 to 103) | 27.29  (18.14 to 41.17) | 2.81  (1.17 to 6.02) | 1.36  (1.17 to 1.56) |
| Panama | 131  (103 to 160) | 75.66  (59.22 to 92.27) | 463  (326 to 625) | 93.74  (66.08 to 126.67) | 2.52  (1.52 to 3.92) | 0.85  (0.78 to 0.93) |
| Papua New Guinea | 80  (40 to 145) | 38.98  (19.43 to 70.74) | 206  (109 to 364) | 43.59  (22.97 to 76.86) | 1.59  (0.66 to 3.24) | 0.26  (0.19 to 0.32) |
| Paraguay | 112  (87 to 166) | 43.25  (33.55 to 64.49) | 608  (396 to 825) | 93.03  (60.55 to 126.28) | 4.45  (2.07 to 7.35) | 3.42  (3.12 to 3.72) |
| Peru | 723  (536 to 1007) | 53.39  (39.54 to 74.35) | 2778  (1861 to 3823) | 73.65  (49.34 to 101.37) | 2.84  (1.28 to 5.17) | 1.48  (1.19 to 1.77) |
| Philippines | 2776  (1641 to 3407) | 84.51  (49.94 to 103.69) | 6551  (5067 to 8231) | 73.58  (56.92 to 92.45) | 1.36  (0.82 to 2.94) | -1.24  (-1.61 to -0.87) |
| Poland | 6404  (5297 to 11816) | 113.11  (93.57 to 208.7) | 17592  (9350 to 21996) | 188.37  (100.11 to 235.52) | 1.75  (-0.14 to 2.98) | 2.06  (1.82 to 2.3) |
| Portugal | 1912  (1639 to 2589) | 103.35  (88.58 to 139.95) | 5193  (4086 to 6030) | 177.53  (139.7 to 206.15) | 1.72  (0.77 to 2.39) | 1.71  (1.39 to 2.04) |
| Puerto Rico | 414  (273 to 504) | 90.38  (59.61 to 109.91) | 886  (556 to 1212) | 98.41  (61.72 to 134.7) | 1.14  (0.54 to 1.9) | 0.26  (0.04 to 0.48) |
| Qatar | 14  (8 to 21) | 161.25  (97.24 to 241.51) | 111  (70 to 167) | 130.41  (82.09 to 196.14) | 7.03  (3.43 to 13.15) | -0.8  (-1.02 to -0.59) |
| Republic of Korea | 1473  (985 to 2688) | 44.34  (29.66 to 80.93) | 10785  (6661 to 13301) | 94.78  (58.54 to 116.9) | 6.32  (1.68 to 10.57) | 3.65  (3.13 to 4.18) |
| Republic of Moldova | 443  (369 to 593) | 79.51  (66.12 to 106.31) | 507  (396 to 603) | 67.31  (52.61 to 80.06) | 0.14  (-0.2 to 0.48) | -0.04  (-0.58 to 0.51) |
| Romania | 2281  (1889 to 3269) | 63.15  (52.29 to 90.5) | 5829  (4577 to 7232) | 118.21  (92.81 to 146.66) | 1.56  (0.71 to 2.39) | 2.67  (2.52 to 2.82) |
| Russian Federation | 15764  (12202 to 18585) | 67.51  (52.25 to 79.58) | 16217  (13647 to 21514) | 52.04  (43.79 to 69.04) | 0.03  (-0.18 to 0.44) | -1.52  (-1.87 to -1.17) |
| Rwanda | 56  (37 to 98) | 16.95  (11.12 to 29.38) | 148  (90 to 235) | 21.94  (13.26 to 34.69) | 1.63  (0.43 to 3.43) | 0.97  (0.8 to 1.14) |
| Saint Kitts and Nevis | 1  (1 to 1) | 12.09  (9.74 to 15.77) | 1  (1 to 2) | 17.27  (13.47 to 21.94) | 1.22  (0.65 to 1.96) | 0.97  (0.74 to 1.2) |
| Saint Lucia | 4  (4 to 6) | 38.85  (31.44 to 52.53) | 12  (10 to 15) | 48.39  (38.2 to 60.06) | 1.85  (1.09 to 2.85) | 0.61  (0.42 to 0.8) |
| Saint Vincent and the Grenadines | 2  (1 to 3) | 18.89  (15 to 30.52) | 7  (6 to 8) | 41.43  (34.02 to 50.28) | 3.03  (1.71 to 4.47) | 2.49  (2.22 to 2.76) |
| Samoa | 7  (5 to 10) | 67.23  (43.77 to 97.47) | 10  (7 to 15) | 62.56  (42.63 to 91.44) | 0.47  (-0.02 to 1.19) | -0.28  (-0.34 to -0.22) |
| San Marino | 7  (6 to 9) | 168.11  (128.07 to 216.12) | 15  (9 to 24) | 203.4  (121.47 to 313.34) | 1.11  (0.26 to 2.57) | 1.02  (0.86 to 1.18) |
| Sao Tome and Principe | 2  (1 to 3) | 25.79  (13.73 to 37.46) | 3  (2 to 5) | 30.94  (13.93 to 45.57) | 0.68  (0.07 to 1.69) | 0.75  (0.69 to 0.8) |
| Saudi Arabia | 494  (206 to 750) | 80.92  (33.75 to 122.84) | 1190  (648 to 1695) | 73.72  (40.11 to 105.01) | 1.41  (0.41 to 3.28) | -0.97  (-1.25 to -0.68) |
| Senegal | 102  (58 to 152) | 27.48  (15.58 to 40.67) | 315  (147 to 461) | 37.07  (17.24 to 54.29) | 2.08  (0.85 to 4.07) | 1.4  (1.18 to 1.63) |
| Serbia | 888  (596 to 1355) | 60.22  (40.4 to 91.92) | 1836  (1320 to 2533) | 83.72  (60.19 to 115.55) | 1.07  (0.36 to 2.27) | 1.65  (1.35 to 1.96) |
| Seychelles | 4  (2 to 5) | 51.48  (28.02 to 76.37) | 8  (4 to 11) | 59.77  (30.45 to 88.71) | 1.1  (0.38 to 2.35) | 0.27  (0.17 to 0.38) |
| Sierra Leone | 49  (27 to 73) | 21.56  (11.93 to 32.51) | 115  (49 to 171) | 29.54  (12.63 to 43.75) | 1.37  (0.39 to 2.96) | 1.47  (1.29 to 1.65) |
| Singapore | 437  (388 to 508) | 172.97  (153.71 to 201.11) | 1528  (1324 to 1728) | 155.19  (134.45 to 175.56) | 2.5  (1.85 to 3.13) | -0.41  (-0.67 to -0.14) |
| Slovakia | 1526  (1040 to 1893) | 196.32  (133.86 to 243.57) | 2302  (1255 to 3169) | 188.17  (102.54 to 259.02) | 0.51  (0.06 to 1.1) | -0.03  (-0.15 to 0.09) |
| Slovenia | 328  (246 to 426) | 106.1  (79.69 to 137.9) | 1174  (664 to 1581) | 215.99  (122.23 to 290.9) | 2.58  (0.95 to 4.41) | 3.12  (2.69 to 3.55) |
| Solomon Islands | 7  (3 to 13) | 46.87  (23.72 to 90.99) | 15  (8 to 27) | 49.48  (27.61 to 87.11) | 1.2  (0.32 to 3.03) | -0.04  (-0.18 to 0.1) |
| Somalia | 36  (18 to 65) | 14.63  (7.59 to 26.75) | 123  (48 to 230) | 17.3  (6.71 to 32.25) | 2.46  (0.7 to 5.33) | 0.88  (0.76 to 1) |
| South Africa | 173  (114 to 331) | 7.15  (4.73 to 13.69) | 500  (366 to 665) | 9.47  (6.92 to 12.59) | 1.89  (0.82 to 3.49) | 1.2  (1.07 to 1.32) |
| South Sudan | 74  (33 to 163) | 27.35  (12.22 to 59.97) | 126  (63 to 233) | 31.93  (15.83 to 58.75) | 0.7  (0 to 1.99) | 0.67  (0.59 to 0.76) |
| Spain | 5848  (4918 to 9057) | 81.41  (68.47 to 126.09) | 18455  (14633 to 21404) | 164.4  (130.36 to 190.67) | 2.16  (0.88 to 3.04) | 2.73  (2.5 to 2.96) |
| Sri Lanka | 727  (533 to 1011) | 58.59  (43 to 81.52) | 2456  (1652 to 3636) | 74.69  (50.23 to 110.56) | 2.38  (1.15 to 4.35) | 0.88  (0.77 to 0.98) |
| Sudan | 864  (420 to 1510) | 81.01  (39.38 to 141.49) | 2076  (1081 to 3366) | 104.77  (54.53 to 169.86) | 1.4  (0.56 to 2.9) | 0.96  (0.86 to 1.06) |
| Suriname | 9  (6 to 13) | 29.94  (21.6 to 43.29) | 28  (20 to 38) | 39.58  (27.96 to 53.49) | 2.11  (1.12 to 3.69) | 1.02  (0.9 to 1.13) |
| Sweden | 3245  (2936 to 3915) | 169.79  (153.64 to 204.84) | 5134  (4435 to 5672) | 202.73  (175.15 to 223.98) | 0.58  (0.33 to 0.78) | 0.49  (0.32 to 0.66) |
| Switzerland | 2363  (1983 to 2802) | 182.95  (153.52 to 216.94) | 3613  (2729 to 4193) | 175.17  (132.32 to 203.29) | 0.53  (0.12 to 0.88) | 0.42  (0.14 to 0.7) |
| Syrian Arab Republic | 1086  (658 to 1688) | 185.01  (112.04 to 287.37) | 2564  (1455 to 4127) | 177.96  (100.98 to 286.51) | 1.36  (0.45 to 2.99) | -0.52  (-0.77 to -0.28) |
| Taiwan (Province of China) | 646  (522 to 921) | 32.41  (26.22 to 46.2) | 5940  (2453 to 8417) | 116.23  (47.99 to 164.71) | 8.2  (2.13 to 13.52) | 7.31  (6.23 to 8.4) |
| Tajikistan | 137  (92 to 205) | 41.85  (28.34 to 62.84) | 322  (222 to 443) | 61.94  (42.6 to 85.11) | 1.36  (0.43 to 3.04) | 1.85  (1.46 to 2.25) |
| Thailand | 721  (496 to 1034) | 18.22  (12.53 to 26.14) | 6163  (2454 to 9276) | 48.5  (19.31 to 73) | 7.55  (2.55 to 14.43) | 4.42  (3.9 to 4.94) |
| Timor-Leste | 11  (6 to 21) | 39.59  (20.9 to 76.95) | 56  (33 to 99) | 54.38  (31.53 to 95.51) | 4.27  (2.22 to 7.74) | 1.36  (1.16 to 1.55) |
| Togo | 34  (19 to 49) | 25.63  (14.12 to 36.75) | 136  (60 to 212) | 35.44  (15.61 to 55.35) | 2.99  (1.37 to 5.83) | 1.25  (1.15 to 1.35) |
| Tokelau | 0  (0 to 0) | 56.67  (31.75 to 89.19) | 0  (0 to 0) | 55.87  (36.26 to 77.67) | -0.13  (-0.44 to 0.37) | -0.12  (-0.24 to 0) |
| Tonga | 3  (2 to 5) | 45.4  (27.54 to 70.68) | 5  (3 to 7) | 51.36  (34.65 to 72.49) | 0.59  (0.04 to 1.48) | 0.26  (0.08 to 0.44) |
| Trinidad and Tobago | 69  (57 to 81) | 67.44  (55.39 to 78.57) | 165  (124 to 218) | 69.32  (52.08 to 91.52) | 1.38  (0.74 to 2.23) | 0.18  (0.06 to 0.31) |
| Tunisia | 312  (220 to 456) | 51.21  (36.17 to 74.98) | 897  (588 to 1378) | 57.97  (37.99 to 89.1) | 1.88  (0.81 to 3.65) | 0.37  (0.31 to 0.43) |
| Turkey | 7039  (5233 to 9316) | 170.54  (126.8 to 225.72) | 18221  (13620 to 23346) | 170.75  (127.64 to 218.78) | 1.59  (0.77 to 2.73) | 0  (-0.09 to 0.08) |
| Turkmenistan | 75  (56 to 110) | 33.9  (25 to 49.39) | 214  (149 to 305) | 49.65  (34.68 to 70.84) | 1.83  (0.98 to 3.28) | 1.55  (1.31 to 1.79) |
| Tuvalu | 1  (0 to 1) | 59.44  (31.37 to 102.86) | 1  (0 to 1) | 57.75  (34.85 to 88.65) | 0.44  (-0.12 to 1.35) | -0.3  (-0.37 to -0.22) |
| Uganda | 107  (67 to 156) | 14.57  (9.12 to 21.21) | 255  (170 to 366) | 17.21  (11.45 to 24.71) | 1.39  (0.44 to 2.8) | 0.68  (0.58 to 0.78) |
| Ukraine | 9685  (7742 to 12508) | 101.99  (81.52 to 131.71) | 8557  (6662 to 11082) | 85.15  (66.29 to 110.27) | -0.12  (-0.35 to 0.21) | -1.04  (-1.2 to -0.88) |
| United Arab Emirates | 68  (30 to 114) | 216.46  (96.42 to 363.06) | 547  (235 to 919) | 204.46  (87.79 to 343.49) | 7.08  (3.5 to 14.48) | -0.47  (-0.79 to -0.14) |
| United Kingdom | 25959  (23381 to 28693) | 221.24  (199.27 to 244.54) | 40545  (33599 to 43022) | 265.82  (220.27 to 282.05) | 0.56  (0.36 to 0.66) | 0.55  (0.43 to 0.67) |
| United Republic of Tanzania | 253  (149 to 448) | 20.04  (11.81 to 35.5) | 730  (460 to 1155) | 27.48  (17.32 to 43.47) | 1.89  (0.84 to 4.05) | 1.35  (1.2 to 1.51) |
| United States of America | 86243  (79084 to 99111) | 210.51  (193.04 to 241.92) | 186339  (157815 to 199751) | 260.96  (221.02 to 279.75) | 1.16  (0.86 to 1.33) | 0.67  (0.53 to 0.82) |
| United States Virgin Islands | 8  (5 to 10) | 77.2  (53.49 to 102.71) | 23  (17 to 29) | 89.27  (66.39 to 113.75) | 1.99  (1.09 to 3.27) | 0.73  (0.59 to 0.87) |
| Uruguay | 578  (463 to 688) | 113.59  (91.11 to 135.23) | 808  (657 to 979) | 121.02  (98.45 to 146.65) | 0.4  (0.1 to 0.76) | -0.1  (-0.26 to 0.06) |
| Uzbekistan | 834  (629 to 1101) | 62.89  (47.46 to 83) | 1618  (1195 to 2192) | 72.65  (53.66 to 98.41) | 0.94  (0.33 to 1.82) | 0.58  (0.33 to 0.82) |
| Vanuatu | 4  (2 to 6) | 49.42  (27.42 to 88.68) | 11  (6 to 18) | 54.19  (31.75 to 89.22) | 2.01  (0.89 to 3.89) | 0.13  (-0.01 to 0.28) |
| Venezuela (Bolivarian Republic of) | 786  (683 to 966) | 71.16  (61.85 to 87.45) | 3510  (2643 to 4619) | 99.3  (74.78 to 130.69) | 3.47  (2.29 to 5.2) | 1.18  (0.87 to 1.48) |
| Viet Nam | 4155  (2849 to 5560) | 84.6  (58.02 to 113.22) | 11091  (7385 to 15638) | 103.8  (69.12 to 146.36) | 1.67  (0.68 to 3.16) | 0.81  (0.74 to 0.88) |
| Yemen | 382  (203 to 609) | 69.71  (37.07 to 111.15) | 1228  (571 to 1990) | 84.49  (39.33 to 136.96) | 2.21  (0.93 to 4.16) | 0.93  (0.82 to 1.04) |
| Zambia | 74  (46 to 130) | 24.09  (14.9 to 42.43) | 205  (136 to 308) | 29.94  (19.85 to 44.92) | 1.77  (0.74 to 3.95) | 0.88  (0.81 to 0.94) |
| Zimbabwe | 84  (52 to 137) | 17.89  (11.06 to 29.27) | 125  (74 to 214) | 16.01  (9.48 to 27.41) | 0.5  (-0.15 to 1.55) | -0.52  (-0.68 to -0.37) |
