# Supplementary figures and images for: Global, national, and regional burden of acute myeloid leukemia among 60–89 years-old individuals: insights from a study covering the period 1990 to 2019
Source: Front Public Health. 2024 Jan 11;11:1329529. doi: 10.3389/fpubh.2023.1329529 (PMC10808630; doi:10.3389/fpubh.2023.1329529)

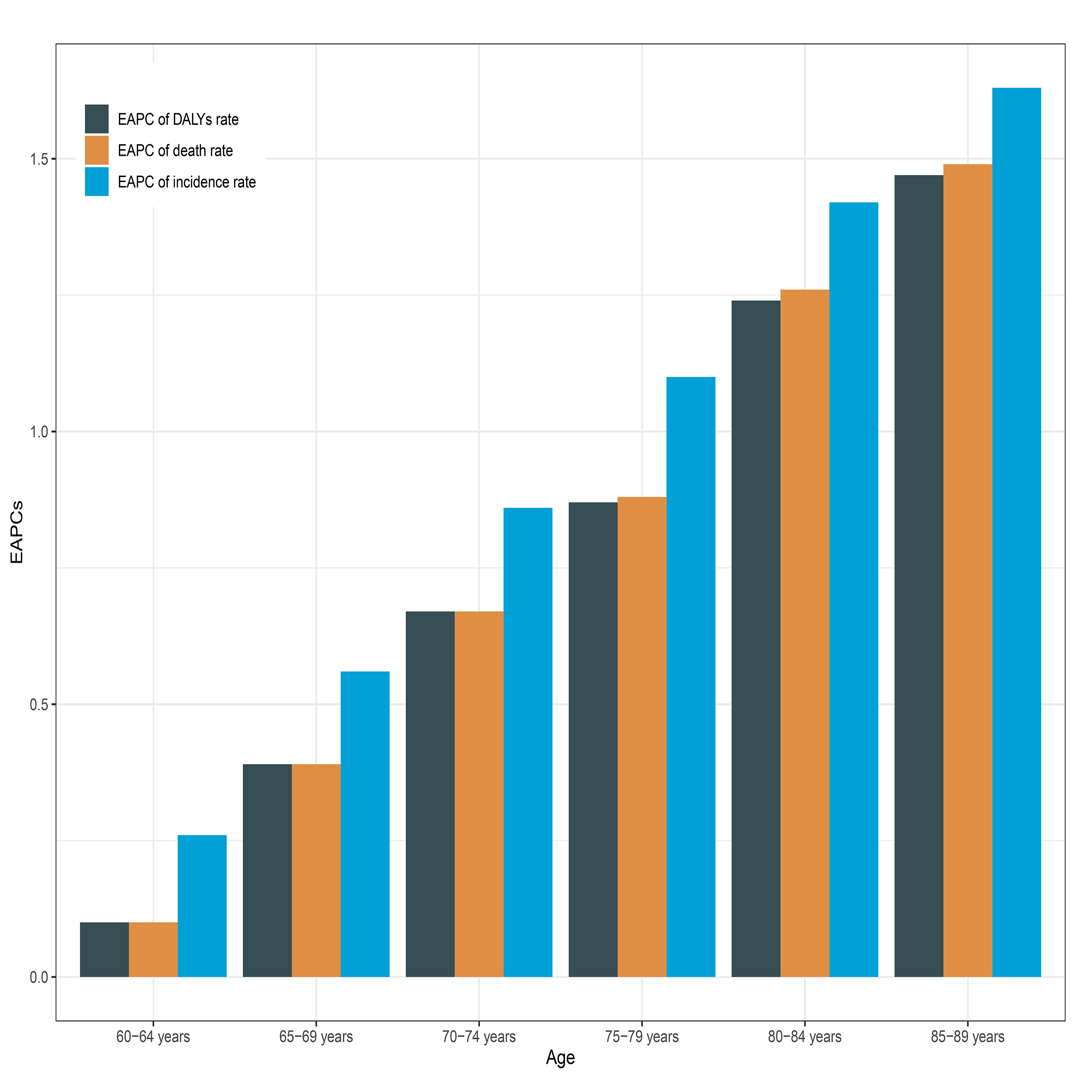

Supplement: Supplementary file 2 [file Image_1.TIF]

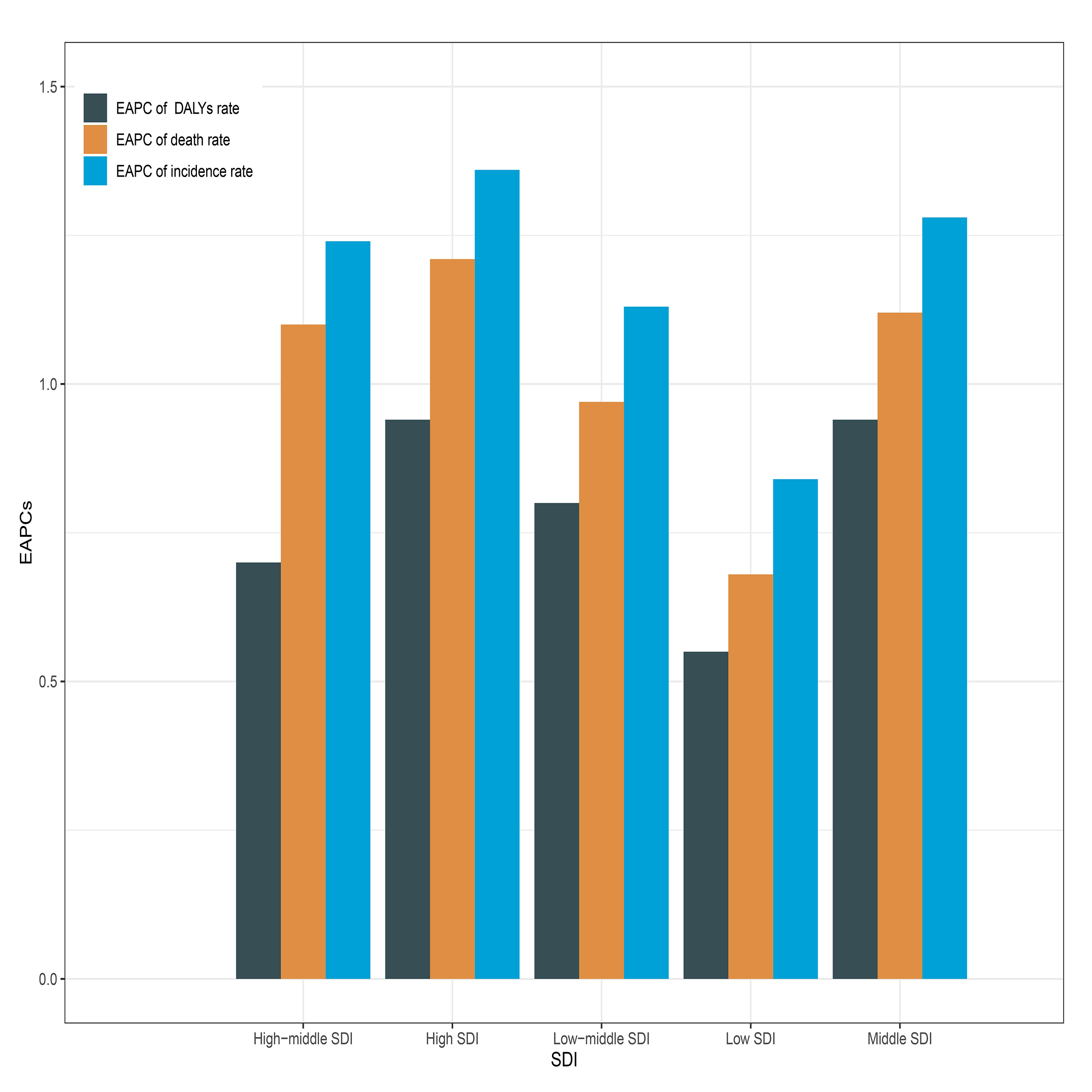

Supplement: Supplementary file 3 [file Image_2.TIF]

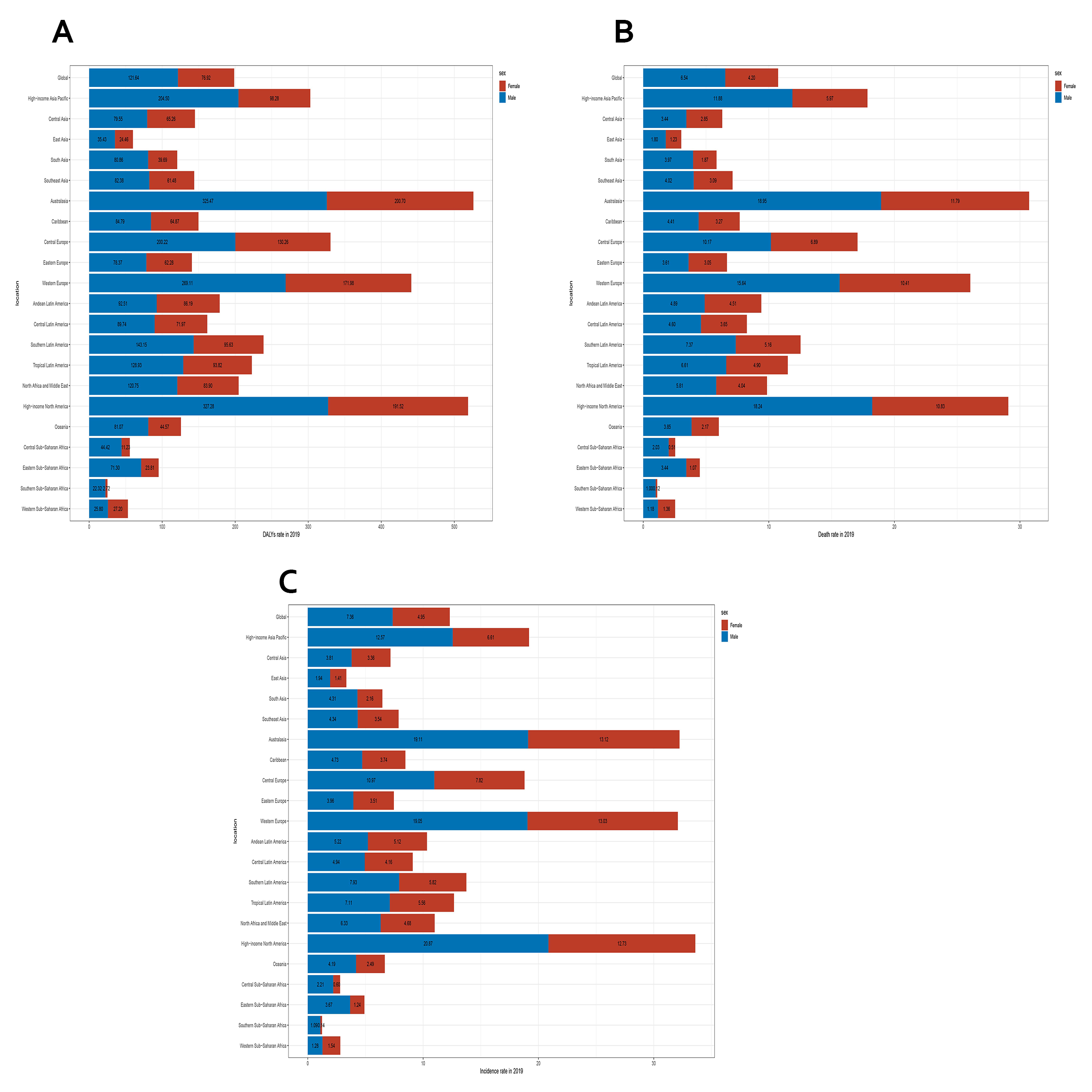

Supplement: Supplementary file 4 [file Image_3.TIF]

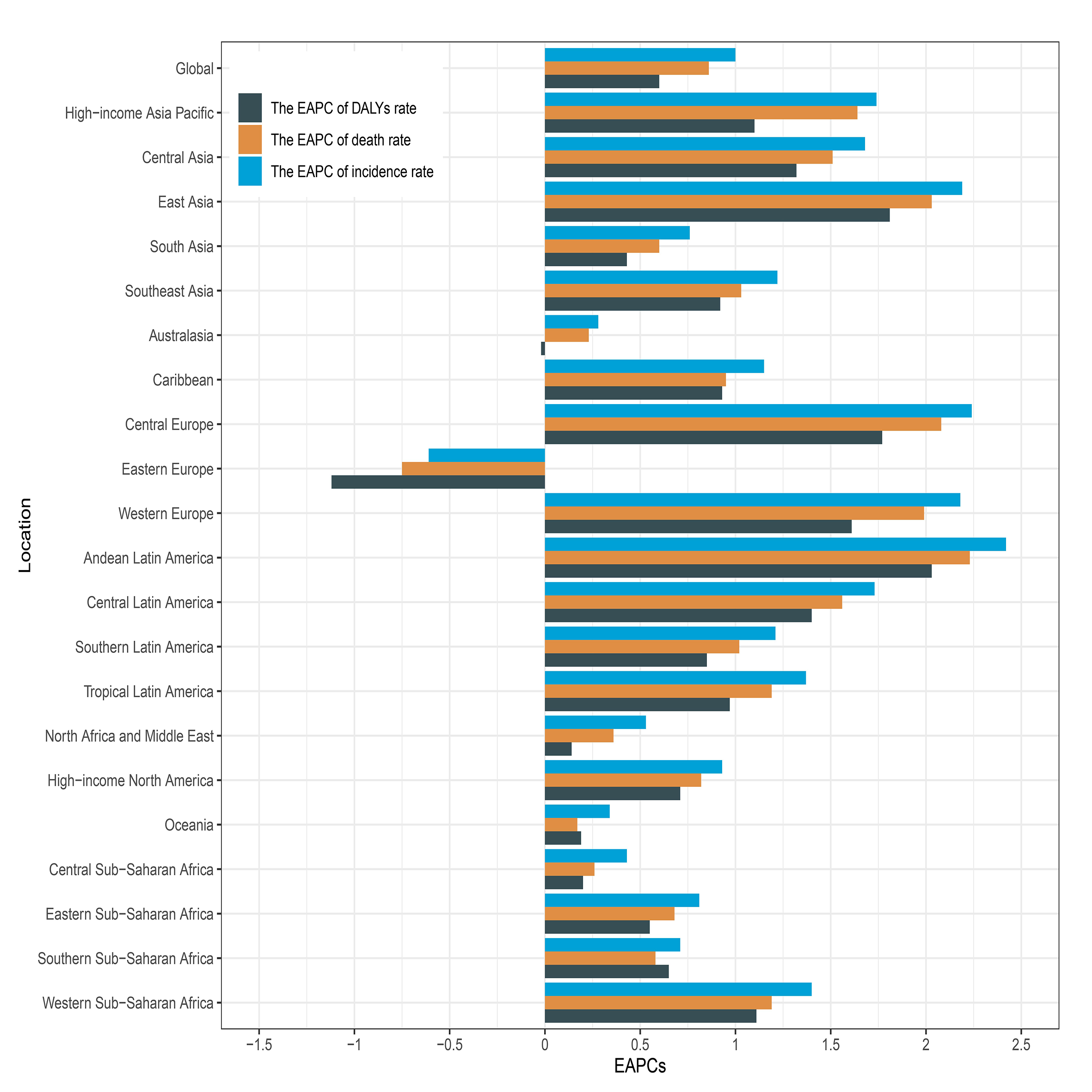

Supplement: Supplementary file 5 [file Image_4.TIF]
